# *FUT2*–*ABO* epistasis increases the risk of early childhood asthma and *Streptococcus pneumoniae* respiratory illnesses

**DOI:** 10.1038/s41467-020-19814-6

**Published:** 2020-12-16

**Authors:** Tarunveer S. Ahluwalia, Anders U. Eliasen, Astrid Sevelsted, Casper-Emil T. Pedersen, Jakob Stokholm, Bo Chawes, Jette Bork-Jensen, Niels Grarup, Oluf Pedersen, Torben Hansen, Allan Linneberg, Amitabh Sharma, Scott T. Weiss, Michael D. Evans, Daniel J. Jackson, Andreanne Morin, Karen A. Krogfelt, Susanne Schjørring, Preben B. Mortensen, David M. Hougaard, Jonas Bybjerg-Grauholm, Marie Bækvad-Hansen, Ole Mors, Merete Nordentoft, Anders D. Børglum, Thomas Werge, Esben Agerbo, James E. Gern, Robert F. Lemanske, Carole Ober, Anders G. Pedersen, Hans Bisgaard, Klaus Bønnelykke

**Affiliations:** 1 COPSAC, Copenhagen Prospective Studies on Asthma in Childhood, Herlev and Gentofte Hospital, University of Copenhagen, Copenhagen, Denmark; 2 Steno Diabetes Center Copenhagen, Gentofte, Denmark; 3 Department of Biology, The Bioinformatics Center, University of Copenhagen, Copenhagen, Denmark; 4 Section for Bioinformatics, Department of Health Technology, Technical University of Denmark, Copenhagen, Denmark; 5 Novo Nordisk Foundation Center for Basic Metabolic Research, Faculty of Health and Medical Sciences, University of Copenhagen, Copenhagen, Denmark; 6 Center for Clinical Research and Disease Prevention, Bispebjerg and Frederiksberg Hospital, The Capital Region, Copenhagen, Denmark; 7 Department of Clinical Medicine, University of Copenhagen, Copenhagen, Denmark; 8 Channing Division of Network Medicine, Department of Medicine, Brigham and Women’s Hospital, Harvard Medical School, Boston, MA USA; 9 Clinical and Translational Science Institute, University of Minnesota, Minneapolis, MN USA; 10 Department of Pediatrics, University of Wisconsin, Madison, WI USA; 11 Department of Human Genetics, University of Chicago, Chicago, IL USA; 12 Department of Bacteria, Parasites and Fungi, Statens Serum Institut, Copenhagen, Denmark; 13 Department of Science and Environment, Roskilde University, Roskilde, Denmark; 14 iPSYCH, The Lundbeck Foundation Initiative for Integrated Psychiatric Research, Copenhagen, Denmark; 15 NCRR, The National Center for Register-based research, Business and Social Sciences, Aarhus University, Aarhus C, Denmark; 16 CIRRAU—Center for Integrated Register-Based Research at Aarhus University, Aarhus C, Denmark; 17 Department for Congenital Disorders, Statens Serum Institut, Copenhagen, Denmark; 18 Den Neonatale Screenings Biobank, SSI, Copenhagen, Denmark; 19 Psychosis Research Unit, Aarhus University Hospital—Psychiatry, Risskov, Denmark; 20 Mental Health Center Copenhagen, Capital Region of Denmark, Copenhagen University Hospital, Copenhagen, Denmark; 21 Department of Biomedicine and iSEQ, Center for Integrative Sequencing, Aarhus University, Aarhus C, Denmark; 22 Center for Genomics and Personalized Medicine, Central Region Denmark, Aarhus C, Denmark; 23 Institute of Biological Psychiatry, Copenhagen Mental Health Services, Copenhagen, Denmark; 24 Center for GeoGenetics, GLOBE Institute, University of Copenhagen, Copenhagen, Denmark

**Keywords:** Genetics, Genetic association study, Genetic interaction, Respiratory tract diseases

## Abstract

Asthma with severe exacerbation is the most common cause of hospitalization among young children. We aim to increase the understanding of this clinically important disease entity through a genome-wide association study. The discovery analysis comprises 2866 children experiencing severe asthma exacerbation between ages 2 and 6 years, and 65,415 non-asthmatic controls, and we replicate findings in 918 children from the Copenhagen Prospective Studies on Asthma in Childhood (COPSAC) birth cohorts. We identify rs281379 near *FUT2*/*MAMSTR* on chromosome 19 as a novel risk locus (OR = 1.18 (95% CI = 1.11–1.25), *P*_discovery_ = 2.6 × 10^−9^) as well as a biologically plausible interaction between functional variants in *FUT2* and *ABO*. We further discover and replicate a potential causal mechanism behind this interaction related to *S. pneumoniae* respiratory illnesses. These results suggest a novel mechanism of early childhood asthma and demonstrates the importance of phenotype-specificity for discovery of asthma genes and epistasis.

## Abstract (web summary)

Genetic variants discovered through genome-wide association studies for asthma together account for a small portion of the heritability. Here, the authors identify a possible epistatic relationship between coding variants in *FUT2* and *ABO*, especially pronounced in severe and early onset asthma.

## Introduction

Asthma with severe exacerbations is the most common cause of hospitalization among young children and has a severe impact on the quality of life and health care costs^1,2^. Exacerbations are typically triggered by respiratory infections, but the host factors causing recurrent infections and exacerbations in some children remain incompletely understood^3^. As a result, current treatment options and preventive measures are inadequate^4^ and there is a need for improved understanding of the underlying pathobiology.

Asthma heritability is reported to be >50%^5^ with higher estimates reported for boys compared to girls^6^ and for disease with early onset^7^. Even though genome-wide association studies (GWAS) have identified several common^8–10^ and low-frequency susceptibility variants^11,12^, these only explain a small proportion of the disease variance observed between individuals, a phenomenon referred to as “missing heritability”^13^. One potential cause for the missing heritability is the heterogeneous nature of asthma, which is a syndrome thought to represent several functional subtypes of disease (endotypes) with different clinical characteristics^14^, such as age at onset and severity. One specific subtype is likely to be closely linked to a specific disease mechanism and might therefore allow detection of subtype-specific susceptibility loci as previously demonstrated in a GWAS of early childhood asthma with severe exacerbations^10^. Another potential explanation for the missing heritability is the interaction between genetic variants, so-called epistasis, although only a few examples of this have been demonstrated in human studies^15,16^.

Here, we aim to improve the understanding of early childhood asthma with severe exacerbations through a large-scale genome-wide association study with a focus on coding gene variants. We utilize genetic data from two Danish case–controls studies (COPSAC_severe_ (COpehangen Prospective Studies on Asthma in Childhood-Severe) and iPSYCH (Integrative Psychiatric Research)), with a total of 68,281 individuals, including 2866 cases, defined as having at least one acute hospitalization due to asthma during the first 6 years of life, and 65,415 non-asthmatic controls. We replicate the findings and further elaborate on the biologically plausible interaction between functional variants of the *FUT2* and *ABO* genes. We also explore potential underlying mechanisms using prospective clinical studies with information on infectious triggers of acute respiratory illnesses.

## Results

### Identification of the *FUT2* region as a susceptibility locus for early childhood asthma

**Fig. 1:**
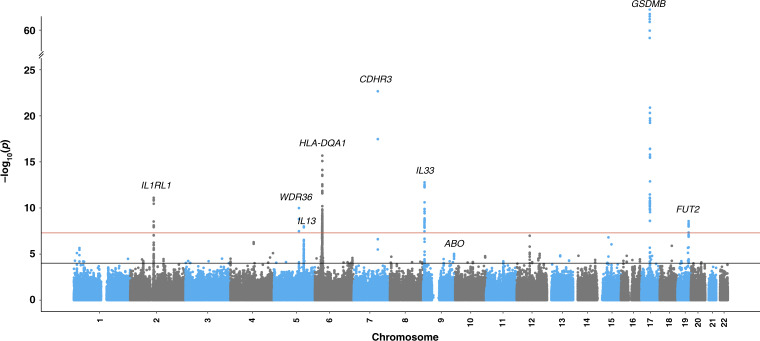
Manhattan plot depicting loci associated with childhood asthma with severe exacerbations in the discovery genome-wide meta-analysis. Association *P* value (suggestive threshold line in black): 1 × 10^−4^, (genome-wide threshold line in red): 5.0 × 10^−8^. Associations are from fixed-effect meta-analyses based on logistic regression models adjusted for sex and the first ten principal components. *N*_discovery_ = 68,281 individuals ($N_{{\mathrm{COPSAC}}_{\mathrm{severe}}}$ = 6532 and *N*_iPSYCH_ = 61,749).

The COPSAC_severe_ study included 1204 children with more than two hospitalizations for asthma and 5328 controls, while the iPSYCH study included 1662 children with at least one hospitalization for asthma and 60,087 controls (Supplementary Table 1). In order to improve genomic resolution in the human leukocyte antigen (HLA) on chromosome 6 and increase the share of functional variants, we imputed HLA types and HLA amino acid polymorphisms, and performed a single single-nucleotide polymorphism (SNP) exome-wide analysis comprising common (minor allele frequency (MAF) ≥ 5%) and low frequency (1% ≤ MAF < 5%) SNPs for each of the two discovery studies^17^. The summary statistics were meta-analyzed using fixed effects with the inverse-variance weighting method. All SNPs reaching genome-wide significance after the discovery meta-analysis and their association summary statistics are listed in Supplementary Data File 1. Nine loci surpassed the genome-wide significance threshold (Fig. 1, Table 1 and Supplementary Fig. 1), of which the *FUT2/MAMSTR* locus on chromosome 19, with the top SNP rs281379 (odds ratio (OR) = 1.18 (95% confidence interval (CI) = 1.11–1.25), *P*_discovery_ = 2.6 × 10^−9^) (Supplementary Fig. 2), emerged as a novel locus associated with asthma. The remaining eight loci were known asthma loci, including rs7219923 in *GSDMB* (OR = 1.65 (95% CI = 1.56–1.75), *P* = 1.6 × 10^−68^)^18^, rs6967330 in *CDHR3* (OR = 1.41 (95% CI = 1.32–1.51), *P* = 2.1 × 10^−23^)^10^, rs1071630 in *HLA-DQA1* (OR = 1.25 (95% CI = 1.18–1.32), *P* = 8.0 × 10^−14^)^19^, rs340933 and rs1342326 in *IL33* (OR = 1.37 (95% CI = 1.26–1.49), *P* = 1.6 × 10^−13^, OR = 1.31 (95% CI = 1.22–1.40), *P* = 1.7 × 10^−13^)^20^, rs10189629 in *IL1RL1* (OR = 1.40 (95% CI = 1.27–1.54), *P* = 7.7 × 10^−12^)^18^, rs1043828 in *WDR36* (OR = 1.20 (95% CI = 1.14–1.27), *P* = 1.0 × 10^−10^)^21^, and rs20541 in *IL13* (OR = 1.21 (95% CI = 1.13–1.29), *P* = 1.0 × 10^−8^)^9^ (Table 1). Conditional analyses suggested two independent signals near *IL33*, where both variants remained genome-wide significant (rs340933: OR = 1.31 (95% CI = 1.21–1.43), *P* = 1.6 × 10^−10^, rs1342326: OR = 1.27 (95% CI = 1.19–1.37), *P* = 1.8 × 10^−11^). There was significant heterogeneity between the discovery studies for four out of nine top loci. As expected, this was consistently due to higher effect sizes in the COPSAC_severe_ cohort with a more severe and specific phenotype, in terms of a higher number of hospitalizations (Table 1 and Supplementary Table 1). Stratified analyses based on the number of hospitalizations consistently showed highest effect sizes in the group of children with most frequent hospitalizations in both COPSAC_severe_ and iPSYCH, and resulted in comparable effect sizes between studies (Supplementary Data File 2). The association patterns between the two discovery cohorts were similar as illustrated by the individual Manhattan plots (Supplementary Fig. 3).

**Table 1:** Genome-wide association study results for childhood asthma with severe exacerbations: discovery meta-analyses for SNPs reaching genome-wide significance (*P* < 5.0 × 10^−8^) and replication plus combined meta-analyses for novel locus.

Chr.	SNP	EA/OA	Nearest gene/s	COPSAC_severe_ *n* = 6532			iPSYCH *n* = 61,749			Stage	OR fixed (95% CI)	*P* value (fixed effects)	OR random (95% CI)	*P* value (random effects)	Het. *P*
				EAF	OR (95% CI)	*P* value	EAF	OR (95% CI)	*P* value						
17	rs7219923^18^	T/C	*GSDMB*	0.51	2.12 (1.93–2.33)	8.6 × 10^−54^	0.48	1.44 (1.35–1.55)	3.5 × 10^−25^	Discovery	1.65 (1.56–1.75)	1.6 × 10^−68^	1.74 (1.19–2.54)	3.3 × 10^−3^	2.54 × 10^−10^
7	rs6967330^10^	A/G	*CDHR3*	0.18	1.50 (1.34–1.69)	3.9 × 10^−12^	0.18	1.36 (1.25–1.48)	2.7 × 10^−13^	Discovery	1.41 (1.32–1.51)	2.1 × 10^−23^	1.41 (1.28–1.55)	1.6 × 10^−13^	0.30
6	rs1071630^18^	C/T	*HLA-DQA1*	0.59	1.36 (1.23–1.49)	6.9 × 10^−10^	0.58	1.19 (1.11–1.28)	2.5 × 10^−6^	Discovery	1.25 (1.18–1.32)	8.0 × 10^−14^	1.27 (1.11–1.44)	3.2 × 10^−4^	0.03
9	rs340933^66^	G/T	*IL33*	0.85	1.51 (1.32–1.74)	5.8 × 10^−9^	0.85	1.30 (1.17–1.44)	1.2 × 10^−6^	Discovery	1.37 (1.26–1.49)	1.6 × 10^−13^	1.39 (1.20–1.61)	2.2 × 10^−3^	0.08
9	rs1342326^66^	C/A	*IL33*	0.17	1.46 (1.30–1.63)	5.1 × 10^−11^	0.15	1.24 (1.13–1.35)	3.9 × 10^−6^	Discovery	1.31 (1.22–1.40)	1.7 × 10^−13^	1.34 (1.14–1.57)	4.4 × 10^−4^	0.02
2	rs10189629^18^	C/A	*IL1RL1*	0.89	1.47 (1.25–1.73)	2.1 × 10^−6^	0.88	1.36 (1.20–1.53)	5.7 × 10^−7^	Discovery	1.40 (1.27–1.54)	7.7 × 10^−12^	1.40 (1.27–1.54)	7.8 × 10^−12^	0.43
5	rs1043828^18^	C/T	*WDR36*	0.37	1.26 (1.15–1.38)	8.1 × 10^−7^	0.36	1.17 (1.09–1.26)	1.3 × 10^−5^	Discovery	1.20 (1.14–1.27)	1.0 × 10^−10^	1.21 (1.12–1.30)	2.1 × 10^−7^	0.21
5	rs20541^18^	A/G	*IL13*	0.21	1.34 (1.20–1.49)	1.2 × 10^−7^	0.21	1.14 (1.05–1.24)	1.3 × 10^−3^	Discovery	1.21 (1.13–1.29)	1.0 × 10^−8^	1.23 (1.05–1.44)	7.5 × 10^−3^	0.02
19	rs281379	G/A	*FUT2, MAMSTR*	0.53	1.18 (1.08–1.30)	2.6 × 10^−4^	0.53	1.18 (1.10–1.26)	3.2 × 10^−6^	Discovery	1.18 (1.11–1.25)	2.6 × 10^−9^	1.18 (1.12–1.25)	3.3 × 10^−9^	0.90
				–	–	–	–	–	–	Replication 1	1.38 (1.09–1.75)	8.4 × 10^−3^	–	–	–
				–	–	–	–	–	–	Replication 2	1.79 (1.00–3.30)	0.05	–	–	–
				–	–	–	–	–	–	Combined replication	1.43 (1.16–1.79)	1.1 × 10^−3^	1.43 (1.15–1.78)	1.2 × 10^−3^	0.42
				–	–	–	–	–	–	Combined	1.20 (1.13–1.27)	9.2 × 10^−10^	1.26 (1.05–1.51)	0.01	0.09

The study-specific logistic regression models were adjusted for sex and ten principal components.

Discovery (*n*: 68,281): COPSAC_severe_ (*n*: 6532) + iPSYCH (*n*: 61,749); replication 1: COPSAC birth cohorts (*n*: 918); replication 2: COAST (*n*: 200); combined replication: replication 1 + replication 2; combined (*n*: 69,393): discovery + combined replication.

Chromosome, EA/OA: effect allele/other allele, EAF: effect allele frequency, OR: odds ratio, CI: confidence interval, Het. *P* heterogeneity *P*: value for meta-analyses.

The large proportion of individuals with psychiatric disease in the iPSYCH cohort did not seem to affect the results since stratified analysis based on psychiatric case/control status showed no significant differences in asthma association for the nine top loci (Supplementary Table 2). Also, despite a higher prevalence of asthma hospitalizations among individuals with psychiatric disease, none of the top asthma SNPs showed association with psychiatric case status (Supplementary Table 3).

The novel *FUT2/MAMSTR* signal was replicated in the COPSAC birth cohorts (COPSAC_2000_ and COPSAC_2010_ combined), where there was evidence of an association between rs281379 and asthma before the age of 6 years (OR = 1.38 (95% CI = 1.09–1.75), *P*_replication 1_ = 0.008). We also found evidence of association between the rs281379 SNP and childhood asthma at the age of 6 years in the COAST (Comparative Outcomes And Service Utilization Trends) cohort (OR = 1.79 (95% CI = 1.00–3.30), *P*_replication 2_ = 0.05) with the combined meta-analysis (discovery and replication stages) remaining genome-wide significant (OR = 1.20 (95% CI = 1.13–1.27), *P*_combined_ = 9.2 × 10^−10^) (Table 1). In addition, the SNP was associated with asthma with onset during the first 6 years of life in the UK Biobank dataset (OR = 1.06 (95% CI = 1.02–1.10), *P* = 0.002, in 5881 cases and 88,097 controls) (Supplementary Table 4), but not with asthma after 6 years of life (OR = 1.00 (95%CI = 0.98–1.02), *P* = 0.96) in 38,860 cases and 88,097 controls). The *FUT2/MAMSTR* top SNP, rs281379, was not associated with lung function or other asthma-related traits in the COPSAC birth cohorts (Supplementary Table 5).

Based on gene expression data from nasal epithelium cells (NECs) obtained from the COPSAC_2010_ cohort (357 children), we examined the presence of expression quantitative trait loci (eQTL) signals for the *FUT2/MAMSTR* top SNP, rs281379. This SNP was a strong eQTL for *FUT2* (Beta = 0.71, (95% CI = 0.66–0.77), *P* = 2.05 × 10^−78^), a strong eQTL for *RASIP* (Beta = −0.42, (95% CI = −0.52–−0.32), *P* = 3.47 × 10^−14^), and a weak eQTL for *FAM83E* (Beta = −0.06, (95% CI = −0.12 to −0.01), *P* = 0.02), and *RPL8* (Beta = 0.03, (95% CI 0.00–0.06), *P* = 0.02) (Supplementary Table 6).

In an attempt to determine the causal variant for the *FUT2/MAMSTR* locus, we applied the Ensemble’s variant effect predictor (VEP) using the SNPs in the 99% credible set (Supplementary Table 7). Among the six SNPs in the credible set, one impacted the *MAMSTR* gene, while the remaining five SNPs affected *FUT2*. Overall, VEP estimated a 59% probability that the true causal variant impacts *FUT2*. The credible set included the functional SNP rs601338 (nonsense mutation, W143*), which was in high linkage disequilibrium (LD, *r*^2^ = 0.81, *D*′ = 0.97) with the top SNP rs281379 in the *FUT2* locus and showed similar evidence of association with asthma (OR = 1.16 (95% CI = 1.09–1.23), *P* = 1.6 × 10^−7^). We further used the Combined Annotation-Dependent Depletion (CADD) database (also PolyPhen2 and SIFT) to identify the functionality (deleterious, disease causal, pathogenicity) of variants at this locus, showing the highest CADD score (48) for the functional *FUT2* SNP, rs601338, followed by rs2287922 related to *RASIP1* with a score of 26 (Supplementary Table 8).

*FUT2* enzyme activity is necessary for secretion of ABO antigens (A/B/H) in body fluids and on epithelial mucosal surfaces determining the “secretor”/“non-secretor” status. The nonsense *FUT2* mutation W143* (rs601338) inactivates the *FUT2* enzyme and homozygosity (genotype AA) results in the “non-secretor” status, whereas genotypes AG/GG result in “secretor” status^22^. The A-allele related to “secretor” status was associated with an increased risk of asthma in the current study.

### *FUT2–ABO* epistatic effects increased the risk of early childhood asthma

Based on the known biological function of *FUT2* for the secretion of A and B antigens on epithelial surfaces, including airway epithelium, we looked for evidence of an association between the *ABO* locus on chromosome 9 and childhood asthma with severe exacerbations. The top signal in the *ABO* region was the intronic *ABO* SNP rs505922, showing a moderate association signal in the discovery stage, albeit without reaching genome-wide significance (OR = 1.13 (95% CI = 1.07–1.20), *P* = 1.11 × 10^−5^). This effect was similar in both discovery cohorts (COPSAC_severe_: OR = 1.15 (95% CI = 1.05–1.27), *P* = 0.0023; iPSYCH: OR = 1.12 (95% CI = 1.04–1.20), *P* = 0.0012). The *ABO* top SNP, rs505922, is in almost complete LD with the frameshift/deletion polymorphism rs8176719 (*r*^2^ = 0.96, *D*′= 1.0), with the rs505922 major (T) allele being correlated with the rs8176719 deletion encoding the O antigen^23,24^. The rs505922 minor (C) allele was associated with increased risk of childhood asthma, indicating that individuals with A, B, or AB blood groups have a higher risk of childhood asthma.

**Fig. 2:**
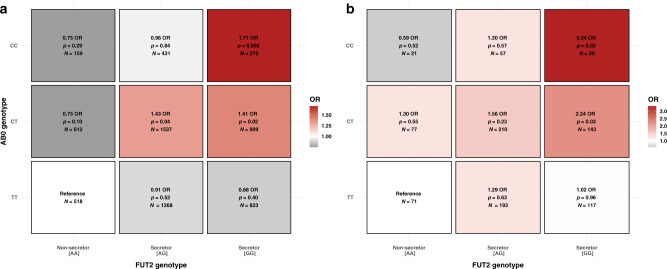
Association **“**heatmaps” for *FUT2 (*rs601338)–ABO (rs505922) genotype combinations. The risk of asthma (OR or odds ratio) was calculated for each genotype combination in relation to the reference group (*FUT2* [AA] and *ABO* [TT]). Heatmaps are shown for **a** the COPSAC_severe_ study (discovery) and **b** the COPSAC birth cohorts (replication). *N*: number of participants in each group. The logistic regression model in **a** is adjusted for sex and ten principal components. The model in **b** is adjusted for sex and cohort status.

**Fig. 3:**
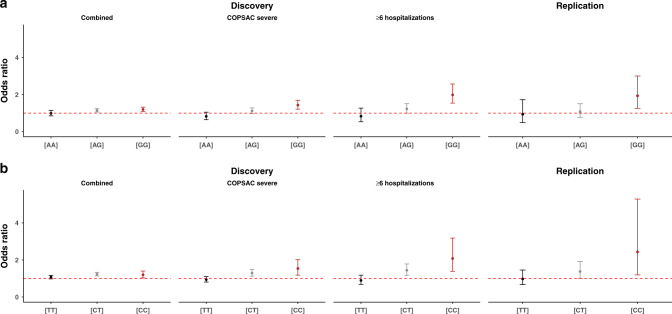
Stratified *FUT2* (rs601338) *and ABO* (rs505922) effects in the discovery and replication stages. For the discovery stage, the effects are shown in strata with increasing phenotype severity, namely the overall stratified results (iPSYCH + COPSAC_severe_), COPSAC_severe_, and the cases with six or more hospitalizations from COPSAC_severe_. **a** illustrates the *ABO* effect stratified on the *FUT2* genotypes (AA, AG, and GG). **b** illustrates the *FUT2* effect stratified on the *ABO* genotypes (TT, CT, and CC). For the discovery analyses, the stratified logistic regression model is adjusted for ten principal components and sex. Replication analyses are adjusted for sex and cohort status. Coloring of the error bars represents the different genotype groups. Error bars represent the 95% confidence interval for each stratified estimate. The estimate is represented by the circles. *N*_combined_ = 68,281 (*N*_cases_ = 2866, *N*_controls_ = 65415). $N_{{\mathrm{COPSAC}}_{\mathrm{severe}}}$  = 6532 (*N*_cases_ = 1204, *N*_controls_ = 5328), *N* ≥ 6_hospitalizations_ = 5707 (*N*_cases_ = 379, *N*_controls_ = 5328). *N*_replication_ = 918 (*N*_cases_ = 191, *N*_controls_ = 727).

The presence of association signals in both the *FUT2* and *ABO* genes raised the possibility that these signals were caused by the same mechanism, namely, secretion of A/B antigens. If this was the case, the two loci should show evidence of interaction in which the *ABO* SNP effect is only evident, or stronger, in children who were secretors based on *FUT2* genotype. Similarly, the *FUT2* effect should only be evident, or stronger, in individuals with A/B blood group-related genotypes. The meta-analysis of the *FUT2–ABO* interaction effect in the discovery stage revealed a borderline significant interaction (OR = 1.08 (95% CI = 0.99–1.17), *P* = 0.06), with significant evidence of heterogeneity between the two discovery studies (heterogeneity *P* = 0.002). While there was no significant interaction in iPSYCH, which has a less specific phenotype definition (interaction OR = 0.98 (95% CI = 0.87–1.09), *P* = 0.73), there was convincing evidence of interaction between *FUT2* and *ABO* in the COPSAC_severe_ study with a more severe and specific phenotype definition (interaction OR = 1.27 (95% CI = 1.11–1.45), *P* = 6.6 × 10^−4^) (Table 2). The strongest interaction signal was seen for the functional *FUT2* SNP (interaction OR = 1.29 (95% CI = 1.12–1.48), *P* = 3.2 × 10^−4^) (Table 2), indicating that the interaction signal could be driven by this SNP. The importance of phenotype specificity for detecting this interaction was further demonstrated by stratifying the cases in the COPSAC_severe_ cohort by severity in terms of the number of hospitalizations (Supplementary Data File 3). While there was no detectable interaction in the children with only two hospitalizations (interaction OR = 1.06 (95% CI = 0.83–1.36), *P* = 0.62), the interaction became evident in the strata with more severe disease, and the strongest interaction signal was seen for children with six or more hospitalizations (interaction OR = 1.51 (95% CI = 1.21–1.88), *P* = 2.6 × 10^−4^) (Table 2 and Supplementary Table 9). A case-only analysis of the interaction showed the same severity dependency, as we only observed a significant correlation between the *FUT2* and *ABO* SNPs using the most severe cases (beta = 0.17 (95% CI = 0.07; 0.27), *P* = 9.2 × 10^−4^) (Supplementary Table 10). We found a tendency of a similar increase in interaction estimate with increased severity in the iPSYCH data (Supplementary Table 9). To investigate the likelihood of observing the same severity-driven interaction signal for the COPSAC_severe_ cohort, we performed 10,000 phenotype permutations, and observed one interaction that showed an interaction effect with similar statistical significance in both the overall analysis and most severe strata. Importantly, the direction of this interaction was as expected from the known “biological interaction” between the two genes, since no effect was observed from the *ABO* variation in non-secretors, while there was an increasing effect with increasing numbers of functional *FUT2* alleles (secretors), as illustrated by *FUT2–ABO*-stratified analyses and risk-determining heat maps (Table 3, Figs. 2 and  3, and Supplementary Data File 3).

**Table 2:** Interaction between *FUT2* and *ABO* in COPSAC_severe_ (discovery).

Stratum	Cases/controls	Interaction *FUT2* (top SNP) × *ABO*		Interaction *FUT2* (functional SNP) × *ABO*	
		OR [95% CI]	*P* value	OR [95% CI]	*P* value
All	1204/5328	1.27 [1.11–1.45]	**6.6** **×** **10**^**−4**^	1.29 [1.12–1.48]	**3.2** **×** **10**^**−4**^
2 hospitalizations	276/5328	1.00 [0.78–1.29]	0.94	1.06 [0.83–1.36]	0.62
3 hospitalizations	240/5328	1.28 [0.98–1.67]	0.06	1.24 [0.95–1.62]	0.11
4–5 hospitalizations	293/5328	1.29 [1.01–1.65]	**0.04**	1.26 [0.99–1.62]	0.06
≥6 hospitalizations	379/5328	1.47 [1.18–1.82]	**5.7** **×** **10**^**−4**^	1.51 [1.21–1.88]	**2.6** **×** **10**^**−4**^

Both the top (rs281379) and functional (rs601338) *FUT2* SNPs were tested in the interaction with the *ABO* SNP (rs505922). The interaction effect was analyzed in four different severity strata in the COPSAC_severe_ study based on the number of hospitalizations. All logistic regression models were adjusted for sex and ten principal components. The reported *P* values are not corrected for multiple comparisons.

*P* < 0.05 is in bold.

**Table 3:** *FUT2* (rs601338) and *ABO* (rs505922) main and stratified effects in the COPSAC_severe_ study (discovery), and the COPSAC birth cohorts (replication).

Discovery (COPSAC_severe_)				
Gene	Analysis	OR [95% CI]	*P* value	Cases/controls
*FUT2*	Main effect	1.17 [1.07–1.29]	**0.001**	1204/5328
	*ABO* = TT	0.94 [0.81–0.10]	0.45	410/2199
	*ABO* = TC	1.29 [1.13–1.48]	**2.0** **×** **10**^**−4**^	623/2435
	*ABO* = CC	1.54 [1.18–2.02]	**0.001**	171/694
*ABO*	Main effect	1.16 [1.05–1.27]	**0.002**	1204/5328
	*FUT2* = AA	0.83 [0.65–1.05]	0.12	194/1095
	*FUT2* = AG	1.12 [0.98–1.28]	0.10	612/2624
	*FUT2* = GG	1.43 [1.21–1.69]	**2.2** **×** **10**^**−5**^	398/1609
*FUT2* × *ABO*	Interaction	1.29 [1.12–1.48]	**3.2** **×** **10**^**−4**^	1204/5328
**Replication (COPSAC birth cohorts)**				
*FUT2*	Main effect	1.31 [1.04; 1.67]	**0.02**	191/727
	*ABO* = TT	0.99 [0.67; 1.47]	0.96	64/317
	*ABO* = TC	1.38 [0.99; 1.94]	0.06	103/327
	*ABO* = CC	2.44 [1.20; 5.30]	**0.02**	24/83
*ABO*	Main effect	1.27 [1.00; 1.62]	**0.05**	191/727
	*FUT2* = AA	0.94 [0.49; 1.73]	0.84	27/142
	*FUT2* = AG	1.10 [0.78; 1.55]	0.57	93/367
	FUT2 = GG	1.93 [1.26; 3.00]	**0.003**	71/218
*FUT2* × *ABO*	Interaction	1.50 [1.05; 2.17]	**0.03**	191/727
	Risk score	2.82 [1.56; 5.10]	**5.7** **×** **10**^**−4**^	191/727

The logistic regression models were adjusted for ten principal components and sex for the discovery and for cohort status and sex for the replication. The reported *P* values are not corrected for multiple comparisons.

*P* < 0.05 is in bold.

We replicated this finding in the COPSAC birth cohorts, which showed a similar statistically significant interaction between the functional *FUT2* SNP and the *ABO* SNP (interaction OR = 1.50 (95% CI = 1.05–2.17), *P* = 0.03) (Table 3). Again, the direction of the interaction was as expected from the known biology and in line with the discovery results as illustrated by *FUT2–ABO* stratified analyses (Table 3 and Figs. 2b and 3). The effect of the *ABO* SNP increased from OR = 0.94 (95% CI = 0.49–1.73), *P* = 0.84, in the *FUT2* (AA) stratum to OR = 1.93 (95% CI = 1.26–3.00), *P* = 0.003, in the *FUT2* (GG) stratum. Similarly, the *FUT2* SNP effect increased from OR = 0.99 (95% CI = 0.67–1.47), *P* = 0.96, in the *ABO* (TT) stratum to OR = 2.44 (95% CI = 1.20–5.30), *P* = 0.02) in the *ABO* (CC) stratum (Table 3).

**Fig. 4:**
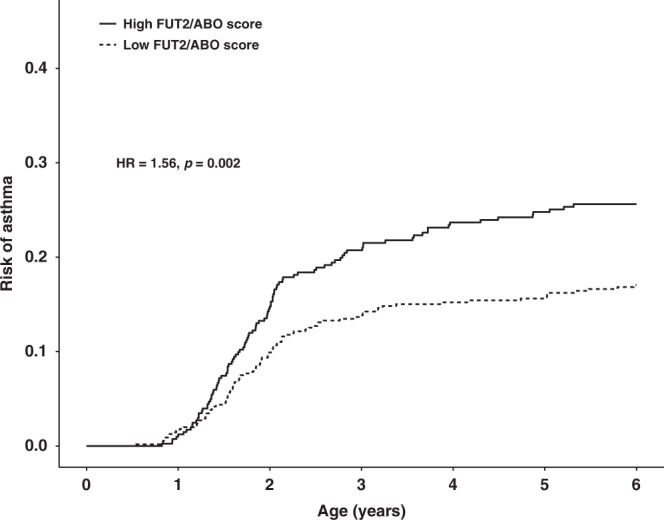
Kaplan–Meier curve illustrating the effect of the *FUT2–ABO* risk score on estimated risk of childhood asthma in the COPSAC birth cohorts (replication). A high *FUT2–ABO* score corresponds to individuals with a score > 0, and a low score corresponds to the remaining individuals with a score < 0. Hazard ratio and *P* value are estimated using a Cox proportional-hazard model. The model was adjusted for sex and cohort status. *N* = 918 (*N*_cases_ = 191, *N*_controls_ = 727). The *FUT2–ABO* score was based on rs601338 (*FUT2*) and rs505922 (*ABO*).

We constructed a combined *FUT2–ABO* risk score to capture the combined effects of the functional *FUT2* and *ABO* variants, as well as their observed interaction in the discovery analysis (“*FUT2–ABO* risk score”). This was done based on the COPSAC_severe_ discovery cohort by calculating risk estimates between a reference group (homozygous for *FUT2 (A)* (non-secretor) and for *ABO (T)* (O blood group) alleles) and each of the eight other possible genotype combinations. The values for this score in each *FUT2–ABO* stratum in comparison to a “standard” additive weighted genetic risk score or simple allele count score are shown in Supplementary Table 11 and Supplementary Fig. 4. The *FUT2–ABO* risk score replicated in relation to asthma before age 6 years in the COPSAC birth cohorts (OR = 2.82 (95% CI = 1.56–5.10), *P* = 5.7 × 10^−4^) (Table 3 and Fig. 4) and with current asthma by age 6 years (OR = 4.71 (95% CI = 1.73–13.07), *P* = 0.003), but not with lung function or other asthma-related traits (Supplementary Table 12).

### *FUT2–ABO* epistasis increased the risk of *Streptococcus pneumoniae* respiratory illnesses

**Fig. 5:**
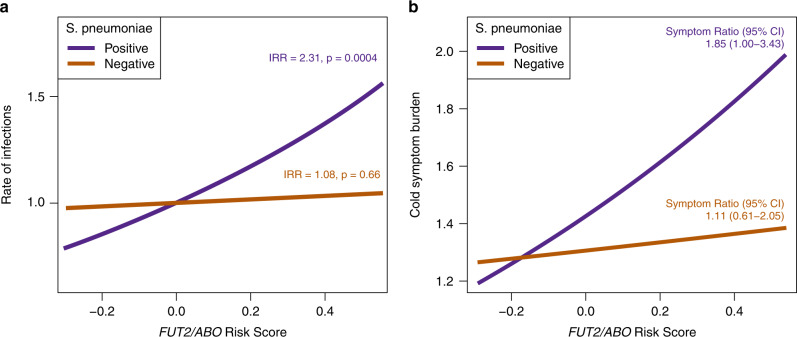
The effect of the *FUT2–ABO* risk score in relation to risk *S. pneumoniae* and non-*S. pneumoniae* respiratory illnesses. **a** illustrates the results in the COPSAC birth cohorts, and **b** results for the RhinoGen study. Incidence rate ratios in **a** are estimated using a quasi-Poisson model, which is adjusted for sex and cohort status. Symptom ratios in **b** are estimated using a mixed-effect quasi-Poison model adjusted for detection of respiratory virus, season, age, sex, race, atopy, and asthma, and a random-effect term for child to account for repeated sampling for each child. **a** *N* = 918 and **b** *N* = 310. The *FUT2–ABO* score was based on rs601338 (*FUT2*) and rs505922 (*ABO*).

Viral and bacterial infections are the main triggers of acute asthma symptoms, and blood group antigens in the airway epithelium have been hypothesized to play an important role for the susceptibility to various infectious agents^25,26^. We, therefore, investigated whether the *FUT2–ABO* gene variation was associated with an increased risk of specific bacterial or viral triggers of acute respiratory illnesses. The analysis was done in the COPSAC birth cohorts where bacterial and viral triggers were analyzed in airway secretions during acute respiratory illnesses in the first 3 years of life. The combined *FUT2–ABO* risk score was significantly associated with increased risk of acute respiratory illnesses with detection of *S. pneumoniae* (incidence rate ratio (IRR) = 2.31 (95% CI = 1.45–3.68), *P* = 0.0004) (Fig. 5), but not with detection of any other bacteria and viruses tested (Fig. 5 and Supplementary Table 13). The *FUT2–ABO* interaction pattern looked similar to the one seen for asthma as illustrated by *FUT2* *×* *ABO* stratified analysis of the risk of respiratory illnesses with detection of *S. pneumoniae* (Supplementary Fig. 5).

We then sought replication of the association between *FUT2* *×* *ABO* and susceptibility to respiratory illnesses with *S. pneumoniae* in the RhinoGen study, from which it has previously been reported that *S. pneumoniae* detection in the airways was associated to the burden of cold symptoms and risk of asthma exacerbations^27^. We hypothesized that this association was related to *FUT2–ABO* genotype and, as hypothesized, a significant interaction was observed between the *FUT2–ABO* risk score and detection of *S. pneumoniae* in relation to duration of upper respiratory tract (cold) symptoms (interaction IRR = 1.66 (95% CI = 1.02–2.70), *P* = 0.04). In line with the COPSAC birth cohort results, the *FUT2–ABO* risk score was associated with increased symptom burden only during episodes with detection of*S. pneumoniae* and not during episodes without detection of*S. pneumoniae* (Fig. 5).

## Discussion

We identified a novel susceptibility locus for early childhood asthma on chromosome 19 near the *FUT2* gene. *FUT2* encodes the enzyme fucosyltransferase 2, which determines the ability to secrete the soluble ABO blood group antigens (ABH) in body fluids and express them on mucosal surfaces, including ciliated columnar cells and cuboidal cells of the bronchial epithelium^28^, oral mucosa^29^, and the gastrointestinal tract^29^. Specifically, the functional nonsense mutation (W143*) rs601338 (in LD with the *FUT2* top SNP rs281379) is the main determinant of the *secretor/non-secretor* status for the ABO antigens (on non-erythrocytes). About 20% of Europeans are homozygous for the rs601338-null alleles (AA), which encodes a stop codon, inactivating the *FUT2* enzyme, classifying them as “*non-secretors*.” We found evidence that secretor status increases the risk of early childhood asthma.

Furthermore, there was evidence of childhood asthma association with the *ABO* SNP rs505922 in our study, in LD with the functional insertion/deletion variant rs8176719, which is the main determinant of the ABO blood group^24^. We observed an increased risk in the non-O group of children, indicating that the presence of A or B antigens increases the risk of developing early childhood asthma.

Importantly, we found evidence of interaction between the functional *FUT2* and *ABO* variants. This interaction is important for several reasons. First, it is a biologically plausible interaction, understood in detail at the biological/biochemical level. The proteins encoded by these two genes are responsible for consecutive steps in a biochemical pathway, each adding a monosaccharide unit to a growing oligosaccharide chain. Specifically, the *FUT2* enzyme adds a fucose unit to a precursor thus creating H-antigen, which is then further modified by the *ABO* enzyme to either A-antigen (by the addition of UDP-GalNAc) or B-antigen (by the addition of UDP-Gal)^22^. This increases the likelihood that it is not a spurious finding. Second, since *FUT2* enzyme activity is necessary for secretion of *ABO* antigens (A/B/H) in body fluids and on epithelial mucosal surfaces, it raises the possibility that the underlying mechanisms of asthma association at these loci could be related to the secretion of *ABO* antigens on epithelial surfaces. While the *ABO* finding alone could be related to non-epithelial mechanisms, for example, via expression on blood cells, and the *FUT2* signal alone could be caused by other nearby genes, a mechanism related to *ABO* antigen secretion seems the most plausible to explain the observed interaction showing no *ABO* effect in *FUT2* determined non-secretors and no *FUT2* effect in individuals with O blood type.

It is likely that a significant proportion of the currently unexplained phenotypic variance (“missing heritability”) is caused by interacting regions in the genome (so-called epistasis). Despite the expected involvement of such genetic interactions in complex diseases, only a very limited number of gene–gene interactions have been discovered and verified in a genome-wide analysis^15,16^. Our study demonstrates that phenotype specificity is important for the ability to detect gene–gene interactions, as evident by a strong interaction observed between the *FUT2* and *ABO* genes only in the most severe asthma cases (Table 2), as well as in the COPSAC birth cohorts with detailed clinical phenotyping. This might have a biological basis due to increased homogeneity and thereby more specific underlying mechanisms where interaction plays a role, or it could be due to higher statistical power to detect the interaction, since the studies where interaction was evident (COPSAC_severe_ and the birth cohorts) also showed higher main effects for *FUT2* and *ABO* individually. The fact that the interaction replicated in the COPSAC birth cohorts, where the asthma phenotype is carefully defined by diary-verified recurrent asthmatic episodes, but mainly include children with milder disease, indicates that the important characteristic allowing detection of interaction in this case was phenotype specificity rather than severity. If the finding that epistasis is only detected using a specific or homogeneous phenotype is a more general phenomenon, it might partly explain the lack of identified epistasis in previous GWAS and suggests that future studies of epistasis should focus on more specific phenotypes.

Several previous studies have investigated the association between ABO blood group secretor status and asthma with inconsistent results. In some of these studies, asthma was associated with secretor status^30^ (136 Taiwanese childhood asthma cases up to 13 years of age and 161 non-asthmatic age-matched controls), while other studies found an association with non-secretor status^31,32^ (165 Italian childhood asthma cases up to 15 years of age and 362 non-asthmatic age-matched controls; 228 French adult coal miners with asthma), or no association^33,34^ (33 Australian adult asthma cases and 127 age-matched non-asthmatic controls; 200 asthma cases (100 children and 100 adults) and 2000 non-asthmatic controls from India). The reason for these conflicting results is probably due to both a low number of individuals reducing the statistical power and lack of phenotype specificity. The *ABO* SNP rs505922 showed some evidence of association with asthma in a previous GWAS but without reaching genome-wide significance (OR = 1.04, *P* = 3.2 × 10^−5^)^35^.

AB antigens are glycans^36^, and presentation of specific glycans in mucus and on the epithelial surface of the respiratory system has been suggested to play an important role for the susceptibility to specific viral and bacterial infections^25,26^. Such respiratory infections are the main trigger of asthmatic symptoms, particularly in childhood^37^, and *FUT2–ABO*-related expression of AB antigens in the respiratory epithelium is, therefore, a biologically plausible mechanism conferring increased susceptibility to the development of early childhood asthma. In addition, previous studies found evidence of selection acting on both *FUT2* and *ABO*, where the coding sequences displayed a higher than expected nucleotide diversity, and demonstrated a positive correlation between allele frequencies and pathogen richness^38,39^. This evidence supports a continuous and critical role of the genes in mediating infection by pathogens. A potential role of *FUT2* for respiratory infections is supported by a recent candidate–gene study reporting association between the *FUT2* rs601338 G allele and a diagnosis of lower respiratory tract illnesses at 12–24 months of age^40^. A GWAS on common infections by Tian et al.^35^ also showed that *FUT2* secretor status was associated with a higher risk of childhood ear infections, and in line with our findings, the *ABO* (rs505922) signal was also present in that study, although no testing for *FUT2–ABO* interaction was reported. Furthermore, *FUT2* gene variation has previously been associated with risk of early childhood diarrheal infections, where secretor status was a risk^41^, and the viral infection mumps, where non-secretor status was associated with increased risk^35^, highlighting the diverse role of the *FUT2*-defined secretor status in relation to specific infections.

We found that the *FUT2–ABO* genotype was specifically associated with acute respiratory illnesses triggered by the bacterium *S. pneumoniae. Streptococcus pneumoniae* is known to cause a range of airway infections, including otitis media and pneumonia^42^, and is a common trigger of acute asthmatic episodes in young children^37^ providing a possible mechanism for the association. Furthermore, this is in line with experimental studies showing that pathogenic microbes often target host glycans for the purpose of nutrition, cell attachment, invasion, and/or immunomodulation^43^, and specifically that fucosylations play an important role for *S. pneumoniae* virulence^44–46^. Interestingly, there seems to be a strain-dependent specificity (based on two fucose-utilizing operons) for harvesting specific fucosylated glycans from blood group antigens. Particularly, *S. pneumoniae* strains carrying the Sp3GH98 operon system have specificity to the A/B antigen fucosylations and are thus predicted to be particularly virulent in individuals with A/B blood types^29^. Such a mechanism could explain our observation of increased risk of *S. pneumoniae* infections in children who are genetically determined secretors of A/B antigens, and we speculate that infections with *S. pneumoniae* subtypes carrying the Sp3GH98 operon system could be driving this association.

If future studies confirm that children with genetically determined secretor status and A/B genotype have an increased risk of specific *S. pneumoniae* infections, it might have clinical implications in terms of a personalized approach to treatment or prevention of infections, for example, by an extended vaccination scheme or a lower threshold for bacterial testing or antibiotic treatment in susceptible individuals. However, any clinical application would require several additional steps, including replication in different populations, establishing causality behind our findings, and randomized trials showing improved clinical benefit according to genotype.

### Strengths and limitations

The diagnosis of asthma is difficult in early life where there is no diagnostic gold standard and many children outgrow their symptoms, and some physicians prefer the term “wheezing” for asthmatic symptoms before age 6 years. However, the specificity of the diagnosis is probably increased in the present study due to the severity of symptoms for the severe asthma cases and the stringent diagnostic criteria applied in the birth cohorts.

It is a limitation of the study that we did not provide experimental data to support the causal mechanisms underlying the observed gene–gene interaction. Another limitation is the reduced genomic resolution with an inherent risk of missing important susceptibility variants, including the true causal variants at the genome-wide significant loci.

The main strength of our study is that we were able to include a relatively large group of individuals with a very specific asthma phenotype, which is likely to increase the study power^10^. Furthermore, asthma heritability seems higher for childhood-onset disease^7^, while most previous asthma GWAS focused on asthma in adults or included a wide age span. This is likely to be the reason for *FUT2* and *ABO* not being detected in the previous GWAS on asthma, including much larger sample sizes^8,9^.

Another strength of this study is that we were able to suggest a potential mechanism related to *S. pneumoniae* infection through assessment of infectious triggers during acute respiratory episodes. Such assessments might be particularly important for understanding the genetic mechanisms of childhood asthma, as also indicated by findings related to *CDHR3* gene variants, another susceptibility locus for early childhood asthma specifically conferring susceptibility to rhinovirus-C infections^47^.

### Conclusion

We identified *FUT2* and *ABO* variation, and epistatic effects between the two, as a genetic mechanism increasing the risk of early childhood asthma and hypothesize that this is related to the expression of AB antigens in the respiratory epithelium and involves infection with *S. pneumoniae*. This is one of the few examples of epistatic effects in asthma, or other complex diseases, and our data suggest that phenotype specificity might be the key to revealing such effects.

## Methods

### Ethics statement

All human research was approved by the relevant institutional review boards and ethical committees and conducted according to the Declaration of Helsinki. All participants and/or their parents in the clinical studies provided written and oral informed consent. COPSAC_2000_, COPSAC_2010_, and Inter99 were approved by the Danish Scientific Ethics Committee, Region H. The registry-based studies (COPSAC_severe_ and iPSYCH) were approved by the Danish Scientific Ethics Committees (Region H and Region Midt, respectively), the Danish Health Data Authority, the Danish data protection agency, and the Danish Neonatal Screening Biobank Steering Committee.

### Governance

The Danish Code of Conduct for Research Integrity and other recognized codes of good research practice have been followed and complied with. The national and international rules on the safety and rights of patients and healthy subjects, including Good Clinical Practice (GCP) as per the EU’s Directive on GCP definition, the International Conference on Harmonization’s (ICH) GCP guidelines, and the Declaration of Helsinki, have been complied with. All national and international legislations involving General Data Protection Regulation (GDPR), the Danish Act on Processing of Personal Data, and the practice of the Danish Data Inspectorate have been followed.

### Study population

The current study comprised a two-stage design: discovery and replication. The discovery set included two case–control studies from Denmark. The first study included childhood asthma exacerbation cases from the COPSAC_severe_ cohort and non-asthmatic adult controls from the Inter99 study cohort, while the second set included cases and controls defined within the iPSYCH study cohort. A total of 68, 281 individuals (2866 cases and 65,415 controls) were part of the discovery set. The replication set comprised 918 children from the COPSAC_2000_ and COPSAC_2010_ cohorts (191 children with asthma and 727 controls without asthma) and the UK Biobank (5881 children with asthma and 88097 non-asthmatic controls).

#### Discovery phase studies

COPSAC_severe_: This is a registry-based cohort constituting children with asthma who have been hospitalized and registered in the national health registries. This study was approved by the Ethics Committee for Copenhagen (H-B-2998-103) and the Danish data protection agency (2008-41-2622). In accordance with the Danish law, the research ethics committee can grant exemption from obtaining informed consent under certain circumstances. For this study cohort, such an exemption was granted (H-B-2998-103).

Identification of children with acute repeated hospitalizations was made from the Danish National Patient Register including all diagnoses and discharge information from specific Danish hospitals^48^. The national birth register was used to obtain information on birth-related events. Inclusion criteria involved the presence of at least two acute hospitalizations relating to asthma (ICD8 codes 493, ICD-10 codes J45-46) for age ranging from 2 to 6 years (both years inclusive). Hospitalization duration had to be >1 day, and two hospitalizations separated by a minimum time period of 6 months. Exclusion criteria included the presence of comorbidities during hospitalization, registered chronic diagnosis considered to affect the risk of hospitalization for asthma, low birth weight (<2.5 kg) or gestational age of child <36 weeks at birth. Further characterization of cases with respect to the number of hospitalizations from asthma and acute bronchitis and concurrent atopy was made. After meeting all inclusion and exclusion criterion, and checking that there was no overlap between COPSAC_severe_ and iPSYCH cases from Denmark, 1204 cases of severe childhood asthma exacerbations were available for the current study (Supplementary Fig. 6).

The control individuals were drawn from a population-based Danish cohort called Inter99^49^. Inter99 is a randomized, non-pharmacological intervention study for the prevention of ischemic heart disease, conducted on 6784 randomly ascertained participants aged 30–60 years at the Research Centre for Prevention and Health in Glostrup, Denmark (ClinicalTrials.gov: NCT00289237). All participants provided informed consent and the study was approved by the Danish Scientific Ethics Committees, region H. Individuals who indicated in a questionnaire that they had physician-diagnosed asthma were excluded, resulting in participation of 5328 non-asthmatic controls from the Inter99 cohort in the current study. In the combined case–control (COPSAC_severe_ and Inter99) dataset, a total of 525,976 overlapping SNPs remained after quality control (QC) (Supplementary Fig. 7).

iPSYCH study: The iPSYCH consortium has established a large Danish population-based case–cohort sample (iPSYCH2012) aimed at unraveling the genetic and environmental architecture of severe mental disorders. The iPSYCH2012 sample is nested within the entire Danish population born between 1981 and 2005, including 1,472,762 persons. The study was approved by the Danish Scientific Ethics Committees (Region Midt), the Danish Health Data Authority, the Danish data protection agency, and the Danish Neonatal Screening Biobank Steering Committee. Dried blood spots for virtually all individuals were retrieved from the Danish neonatal screening biobank and processed for genotyping and GWAS using the DNA amplification method^50^. More information on this initiative can be obtained from http://ipsych.au.dk/about-ipsych/.

Asthma cases within the iPSYCH study were defined using registry data from the National Patient Registries. Individuals with at least one hospitalization due to a primary diagnosis of asthma exacerbations (ICD8-codes 493, ICD-10 codes J45-46) in the first 6 years of life were classified as asthma cases. The remaining individuals without hospitalizations due to severe asthma exacerbations were used as controls.

After QC and admixture analysis, a total of 61,749 (72%) individuals of European ancestry remained of the original 86,189 individuals included in the iPSYCH study^51^. One thousand and sixty-two asthma cases and 60,087 non-asthmatic controls with both genotype and phenotype data participated in the current study (Supplementary Fig. 8).

#### Replication phase studies

The COPSAC_2000_ and the COPSAC_2010_ cohorts were combined and used as one replication cohort comprising 918 children (including 191 childhood asthma cases and 727 non-asthmatic controls) with complete genotype and phenotype information. COPSAC_2000_ is a mother–child cohort where all the mothers had a history of a doctor’s diagnosis of asthma after 7 years of age and thus this is a high-risk asthma cohort comprising 411 children. Newborns were enrolled in the first month of life, and details on the cohort have been described previously^52^. COPSAC_2010_ is a mother–child cohort comprising 700 children born to unselected mothers from Denmark as described previously in detail^53^. In both studies, asthma was diagnosed by doctors in the research clinic based on a previously detailed quantitative symptom algorithm^54,55^ requiring all of the following criteria: (1) verified diary recordings of five episodes of troublesome lung symptoms (cough, breathlessness, or wheeze) within the preceding 6 months, each lasting at least 3 consecutive days; (2) symptomatology typical of asthma, including exercise-induced symptoms, prolonged nocturnal cough, and persistent cough outside common cold; (3) the rescue use of inhaled β2-agonist; and (4) response to a 3-month course of inhaled corticosteroids, followed by relapse after the end of treatment. Remission was defined as a period of 12 months without relapse. The studies were conducted in accordance with the guiding principles of the Declaration of Helsinki and were approved by the Local Ethics Committee (COPSAC_2000_: KF 01-289/96; COPSAC_2010_: H-B-2008-093), and the Danish Data Protection Agency (COPSAC_2000_ and COPSAC_2010_: 2015-41-3696). Both parents provided written informed consent before enrollment.

As part of the replication, 289 newborns from the COAST study, from Madison, Wisconsin, were enrolled between November 1998 and May 2000, as described previously^56^. At least one parent of the COAST cohort children had respiratory allergies, a history of physician-diagnosed asthma, or both. The parents of the participating newborns (*n* = 214) of European ancestry gave consent for their child to participate in genetic studies, and the current study analysis includes these data. 200 children were evaluated for asthma beginning at 6 years of age. Diagnosis for current asthma was made at the end of the sixth year of life, basis the documented presence of one or more of the following characteristics during the previous year: (1) physician diagnosis of asthma, (2) use of albuterol for coughing or wheezing episodes (prescribed by a physician), (3) use of a daily controller medication, (4) step-up plan including the use of albuterol or short-term use of inhaled corticosteroids during illness, and (5) use of prednisone for asthma exacerbation. Four separate investigators, independently evaluated each subject for the presence or absence of asthma while being blinded to any antecedent histories concerning viral illnesses or patterns of aeroallergen sensitization, based on the above criteria.

### Genotyping, SNP calling, and quality control

Each study had genotyping performed using genome-wide arrays followed by study-specific quality filters prior to imputation. The current study focuses on exome-rich genomic arrays and is limited to non-imputed genotype data-based associations.

COPSAC cohorts: DNA sampling for COPSAC_severe_ cases has previously been described in detail^11^. All COPSAC cohorts (COPSAC_severe_, COPSAC_2000_, COPSAC_2010_) were genotyped on the Illumina HumanOmniExpressExome 8 v1-2 BeadChip (951,117 SNPs) at the AROS Applied Biotechnology AS Center, Aarhus, Denmark. SNP genotype calling for COPSAC_severe_ and COPSAC birth cohorts was performed using the GenCall followed by zCall^49^. SNPs with (a) MAF > 0.01, (b) cluster separation score ≥0.3, (c) individual call rate >99%, (d) SNP call rate >99%, and (e) Hardy–Weinberg equilibrium (HWE) *P* values > 10^−6^ were included. Individuals with a sex mismatch were excluded. Individuals not clustering with the European ancestry (Utah residents with ancestry from Northern or Western Europe (CEU)) were excluded using the multidimensional scaling analyses seeded with individuals from the International HapMap 3 project. Following this pairwise identity-by-decent estimates were calculated in the genetically homogenous (European) individuals where monozygotic twins or genetic duplicates were excluded for the COPSAC_severe_ cohort.

Inter99: Genotyping of the Inter99 cohort was performed on the Illumina HumanExome BeadChip 12.v.1.0 (*n* = 280,234 SNPs)^49^ and the Illumina Infinium OmniExpress-24 BeadChips; (710,000 SNPs) and merged. Detailed information on genotyping and QC for the Inter99 study^49^ has been provided in the Supplementary Fig. 7.

COPSAC_severe_ cases and Inter99 non-asthmatic controls were finally merged for 525.976 overlapping SNPs forming a case-control dataset (*n*_cases_ = 1204, *n*_controls_ = 5328).

All genotype QC was performed using PLINK v.1.90^57^ (www.cog-genomics.org/plink/1.9/).

iPSYCH: iPSYCH samples were processed at the Broad Institute (Boston, MA, USA) using the Infinium PsychChip v1.0 array (Illumina, San Diego, CA, USA) in accordance with the manufacturer’s instructions. Individuals with a homogenous genetic background (after global and local ethnicity filtering using PCA and Mahalanobis distance criteria) were checked for various degrees of kinship using the KING (v1.9, October 2015) software package (http://people.virginia.edu/~wc9c/KING/). All “second-degree” relatives were excluded ensuring no two subjects were closer than third-degree relatives. Study-specific genotyping and QC details have been elaborated previously^51^ and also under Supplementary Fig. 8. After QC, the iPSYCH study contributed with 1662 childhood asthma cases and 60,087 non-asthmatic controls (Supplementary Fig. 8).

COAST and RhinoGen: Peripheral blood samples were collected and aliquots of frozen blood were shipped on dry ice to the University of Chicago, where DNA was extracted using the Puregene extraction kit, following the manufacturer’s instructions (Gentra Systems, Inc., Minneapolis, MN). Genotyping was done using the Illumina GoldenGate custom genotyping assay at the National Heart, Lung, and Blood Institute’s (NHLBI) Resequencing and Genotyping Service at Johns Hopkins University. QC parameters were SNP call rate >95% call rates, Hardy–Weinburg *P* values > 0.001, and MAFs >5% and European ancestry (using the multidimensional scaling analyses seeded with individuals from the International HapMap 3 project).

UK Biobank analysis was based on the age of asthma (field 3786) and age of doctors diagnosed asthma (field 22147). We only used genotype data from the European unrelated population. Analysis was adjusted for ten PCs and gender. Details on the UK Biobank genotyping and QC have been described previously^58^.

#### SNP annotation

The Genome Variation Server (https://gvs.gs.washington.edu/GVS138/HelpAbout.jsp), which is also a part of the Seattle Sequence annotation database (https://snp.gs.washington.edu/SeattleSeqAnnotation154/HelpAbout.jsp), was used for annotating the SNPs on the Exome chip/s in the current study. This has also been incorporated by the NHLBI Exome sequencing project-based Exome Variant Server (http://evs.gs.washington.edu/EVS/) for annotating exome-based gene variants.

### Statistical analyses

*GWAS meta-analysis*: We ran a GWAS among childhood asthma exacerbation cases vs. non-asthmatic controls for the two (case–control) discovery cohorts, followed by a discovery stage meta-analysis. Followed by this, we identified childhood asthma loci reaching genome-wide significance and replicated them in COPSAC birth cohorts, followed by a combined meta-analysis of the top loci.

The discovery meta-analysis was performed using the METAL^59^ software (https://genome.sph.umich.edu/wiki/METAL) whereas the replication-based combined meta-analysis for the top SNPs was performed using R meta package (Meta v4.8-4)^60^.

*Discovery phase*: This was based on two GWASs: (1) 1204 cases (COPSAC_severe_) and 5328 controls (Inter99 study) and (2) 1662 cases and 65,415 controls (iPSYCH study), a combined discovery meta-analysis comprising 2866 childhood asthma cases and 65,415 control individuals.

Here, the single-SNP association testing included an additive genetic model based on the logistic regression analyses with a binary outcome (presence or absence of asthma) using Plink 1.9^57^. This model was adjusted for sex and population substructure defined by the first ten PCs (childhood asthma–SNP + sex + first 10 PCs). Prior studies have shown evidence of shared mechanisms between asthma and psychiatric disorders^61^; therefore, the presence/absence of reported psychiatric disorders in the iPSYCH study was included as a binary covariate for the iPSYCH analysis.

Following this, we ran an inverse variance-based fixed-effects meta-analyses between the two participating discovery cohorts using METAL (version March 2011)^59^. Since the discovery stage study cohort cases differ in asthma severity (Supplementary Table 1) and in order to have a comparative overview of how the variants perform assuming random effects, we also ran the random-effects meta-analysis model. Testing for heterogeneity was also performed as part of the meta-analysis package using METAL where *I*^2^ statistic denoting the percentage of variation across studies was estimated (*I*^2^ = 100% × (*Q* − d.f.)/*Q*), where *Q* is the *χ*^2^ statistic. Significance for heterogeneity was denoted by the heterogeneity (or Het_*P*_) *P* values.

*Replication phase*: All independent SNPs reaching a *P* value < 5.0 × 10^−8^ in the discovery phase was subsequently used in the replication phase using genotype data from the two COPSAC birth cohorts. The design of the cohorts is inherently different, as the children in COPSAC 2000 all have asthmatic mothers (Supplementary Note 1). To alleviate this problem, we generated a binary variable, indicating what cohort the individual child belonged to. The SNP effects on asthma during the first 6 years of life was estimated using logistic regression, with gender and the binary cohort variable as additional covariates. Children without any asthma diagnosis and incomplete follow-up at 6 years of age were removed from the analysis. The results of the logistic regression were summarized using ORs, 95% CIs, and *P* values.

We also performed replication analysis for the novel top SNP/SNPs in the COAST birth cohort and the UK Biobank Study set using a logistic regression model (childhood asthma–SNP + sex + first 10 PCs).

*LD-based SNP clumping and conditional analysis*: LD-based SNP clumping technique was utilized to identify index SNPs for each associated locus accounting for the LD structure (at *r*^2^ = 0.10) and a genomic window size of 1 Mb, using Plink^57^. We performed conditioning for index SNPs within loci showing multiple signals exceeding genome-wide significance and sharing a low LD between each other (*r*^2^ < 0.02) after clumping. This helped us identify if two or more signals within a defined region were independent or not. Ancestry-specific LD information was also verified using National Institutes of Health-based LDlink database^49^ (https://ldlink.nci.nih.gov/).

*Fine-mapping*: We applied Bayesian fine-mapping on the discovery summary statistics for the *FUT2* locus as previously described^62^. The region was defined as all SNPs in ±500 kb from the lead SNP in the region. The fine-mapping is done by calculating the posterior probability that a variant in the region is causal. The variants are subsequently sorted according to their posterior probability, and the 99% credible set of SNPs is defined as those that have a cumulative probability of at least 99%. The credible sets were calculated using the corrcoverage package in R (https://annahutch.github.io/corrcoverage). We further used the CADD database, v1.6, and PolyPhen2, v2, and SIFT to identify the functionality (deleterious, disease causal, pathogenicity) of variants at this locus.

*eQTL analysis using nasal epithelial cells*: We obtained RNA sequence data from NECs of 357 children from the COPSAC_2010_ birth cohort at 6 years visit. Raw fastQ files were merged and were aligned to the human reference genome using the STAR software version 2.5.1a^63^. For each gene available in our data, a gene count number was available from STAR, projecting the level of expression, that is, the higher the number of mRNA transcripts, the higher the expression level. Normalization of the gene count data was done using the TMM (trimmed mean of *M* values) method, which is used to account for library size variation between samples^63,64^. Following this step, samples were converted to log counts per million, and mean-variance relationships were found to identify proper weights using the software voom^65^.

*Phenotype permutations and severity stratification*: In order to investigate the likelihood of observing a similar overall interaction effect, which was primarily driven by the most severe group of cases, we performed 10,000 phenotype permutations. For each permutation run, we shuffled the IDs in the COPSAC_severe_ cohort and calculated the overall interaction effect between the *FUT2* and *ABO* variants, and, subsequently, the severity-stratified interaction effect, using the original case information on the number of hospitalizations. We extracted the interaction estimates for each permutation run and compared these to the observed values. The permutation analysis was done using R version 3.4.4

*FUT2–ABO asthma risk score*: Given two SNPs with an additive genotype coding, nine possible genotype combinations exist. Of these nine groups, the only group without any risk alleles consists of individuals who are homozygous for both non-risk alleles, whereas the remaining eight genotype groups all have at least one risk allele. In order to capture *FUT2* and *ABO* main effects as well as the observed interaction pattern between the two, we generated a *FUT2–ABO* risk score by estimating the risk of asthma in each of these eight genotype strata compared to the reference group with no risk allele copies. The effect of the specific genotype strata in relation to asthma was estimated using logistic regression, and adjusted for ten principal components and sex, and the Beta estimate was used as a score for individuals in the respective strata. Supplementary Table 11 illustrates the estimated effect for each of the eight genotype groups. Standard allele counts and additive risk scores would assign risks to every genotype group with at least one risk allele (Supplementary Fig. 4), whereas the *FUT2–ABO* interaction score separates itself by assigning no effects to the non-secretor (*FUT2* [AA] genotype) and O blood groups (*ABO* [TT] genotype), hereby capturing the biologically plausible pattern of *FUT2–ABO* interaction observed in the discovery stage. The *FUT2–ABO* score is therefore primarily driven by individuals who are AB secretors. We used the COPSAC_severe_ (discovery stage) data to derive the *FUT2–ABO* interaction score using the functional *ABO* and *FUT2* SNP. The interaction score was applied in the downstream analysis in terms of replication of the asthma association in the COPSAC birth cohorts, association to asthma-related traits, and association to acute respiratory illnesses with detection of different bacterial and viral triggers. The logistic regression model was done using R version 3.4.4.

*Risk of bacterial and viral triggers of acute respiratory illnesses*: Both COPSAC birth cohorts include data on potential bacterial and viral triggers during acute respiratory illnesses in the first 3 years of life^47^. We investigated whether the *FUT2–ABO* risk score was associated with an increased risk of acute respiratory illnesses with detection of specific bacterial or viral triggers during the first 3 years of life. Only children with full follow-up for the first 3 years were included in the analyses. Children without any acute visits were assumed to have no episodes. We tested the effect of the *FUT2–ABO* risk score on 11 different infectious triggers, 3 of which were bacterial infections and 8 were viral infections. We applied a quasi-Poisson model to account for the observed overdispersion. Each effect estimate was adjusted for sex and cohort status. Results were summarized using the IRR, quantifying the increase in frequency ratio per copy of effect allele, 95% CIs, and *P* values. The quasi-Poisson model was fitted using the glm command in R version 3.4.4. The reported *P* values were not corrected for multiple testing.

*Association to other related traits*: We examined the association of the index SNP (and the *FUT2–ABO* risk score) with lung function measures in (a) neonates (provocative dose 15, forced expiratory flow at 50%, and forced expiratory volume at 0.5 second (FEV0.5)), and (b) children at 6 years of age (FEV1, forced vital capacity (FVC), FEV1:FVC ratio before and after bronchodilation). In addition, we assessed similar associations with allergic sensitization and atopic dermatitis (eczema) at the 6 years visit within the COPSAC birth cohorts. Each (linear or logistic) model was adjusted for sex and study cohort.

*RhinoGen replication analysis*: The RhinoGen study included 167 outpatient children with asthma and 143 children without asthma aged 4–12 years, and was conducted to identify genetic and microbial associations with respiratory illnesses and exacerbations of asthma^27^. During peak cold seasons in April and September, samples of nasal mucus were collected weekly and 3028 specimens were analyzed using RT-PCR for common respiratory viruses and three bacterial pathogens (*S. pneumoniae*, *Moraxella catarrhalis*, and *Haemophilus influenzae*) as previously described^27,66^. Respiratory symptoms were recorded in diaries. The children were scored for cold symptom severity based on a four-point scoring system (0 = no symptoms, 1 = “mild stuffy or runny nose but does not affect daily activity,” 2 = “moderate stuffy or runny nose and reduced activity but does not affect sleep,” 4 = “cannot breathe through the nose and not able to sleep well because of symptoms”). Written informed consent was obtained from the parents, and written assent was obtained from children aged 7 years and older. The study was approved by the University of Wisconsin Human Subjects Committee (H-2007-0136-CR008).

To examine the relationship between *FUT2–ABO* risk score, *S. pneumoniae* detection, and cold symptoms, a mixed-effects quasi-Poisson regression model was fit. The model response was the weekly cold symptom burden (the sum of the cold symptom scores during the 7-day period centered at the day of nasal sampling). Model covariates included fixed-effect terms for *FUT2–ABO* risk score,*S. pneumoniae* detection, *FUT2–ABO* risk score-by-*S. pneumoniae* interaction, detection of respiratory virus, season, age, sex, race, atopy, and asthma, and a random-effect term for child to account for repeated sampling of children. Testing for a differential effect of *FUT2–ABO* risk score according to the presence or absence of *S. pneumoniae* was done by testing the significance of the interaction term, and the coefficient for that term was exponentiated to provide an estimate of the ratio of the change in cold symptom burden with respect to a one unit increase in *FUT2–ABO* risk score in the presence of *S. pneumoniae* to that in the absence of *S. pneumoniae*.

### Reporting summary

Further information on research design is available in the Nature Research Reporting Summary linked to this article.

## Supplementary information

Supplementary Information

Peer Review File

Reporting Summary

Description of Additional Supplementary Files

Supplementary Data 1

Supplementary Data 2

Supplementary Data 3

## Data Availability

GWAS summary statistics are available at Figshare (10.6084/m9.figshare.13161098.v2). All other data that support the findings of this study are available from the corresponding author K.B. upon reasonable request. Participant-level personally identifiable data are protected under the Danish Data Protection Act and European Regulation 2016/679 of the European Parliament and of the Council (GDPR) that prohibit distribution even in pseudo-anonymized form, but can be made available under a data transfer agreement as a collaboration effort.
