# Supplementary material for: FUT2–ABO epistasis increases the risk of early childhood asthma and Streptococcus pneumoniae respiratory illnesses
Source: Nat Commun. 2020 Dec 16;11:6398. doi: 10.1038/s41467-020-19814-6 (PMC7744576; doi:10.1038/s41467-020-19814-6)
Supplement: Supplementary file 1 — Supplementary Information [file 41467_2020_19814_MOESM1_ESM.pdf]

Supplementary Material

***FUT2-ABO* epistasis increases the risk of early childhood asthma and *Streptococcus pneumoniae* respiratory illnesses**

INDEX

I. Supplementary Note 1

1. Study cohorts and Phenotyping

II. Supplementary Tables

Supplementary Table 1. Study characteristics.

Supplementary Table 2 Index SNP-asthma associations stratified on iPsych cohort by psychiatric case-control status

Supplementary Table3. Associations between index SNP and psychiatric disease status in the iPSYCH cohort

Supplementary Table 4. Genome-wide association study results for severe childhood asthma exacerbations: discovery meta-analyses and replication for top SNPs reaching genome-wide significance ($p_{discovery} < 5.0 \times 10^{-8}$)

Supplementary Table 5. Association of *FUT2* rs281379 with childhood lung function, allergic sensitization, and eczema in COPSAC birth cohorts.

Supplementary Table 6. eQTL analysis for the top *FUT2* variant in relation to the genes in genomic proximity.

Supplementary Table 7. Variant effect predictor results for SNPs in the 99% credible set for the *FUT2* locus.

Supplementary Table 8. Combined annotation dependent depletion (CADD) scores for functional prediction of *FUT2* locus SNPs.

Supplementary Table 9. *FUT2* and *ABO* interaction stratified by severity in iPSYCH and $COPSAC_{severe}$.

Supplementary Table 10. Case-only *FUT2* and *ABO* interaction stratified by severity in $COPSAC_{severe}$

Supplementary Table 11. Deriving the *FUT2-ABO* combined risk score

Supplementary Table 12. Association of *FUT2-ABO* combined risk score with lung function, allergic sensitization, eczema and eosinophil counts in COPSAC birth cohorts

Supplementary Table 13. Association between the *FUT2-ABO* combined risk score and number of bacterial infections ever in early life (0-3 years) in the combined prospective COPSAC birth cohorts ($COPSAC_{2000}$ and $COPSAC_{2010}$).

III. Supplementary Figures

Supplementary Figure 1. QQ Plot (Discovery GWAS Meta analyses)

Supplementary Figure 2. Regional Plot for Chromosome 19 *FUT2/MAMSTR* locus

Supplementary Figure 3 Manhattan plots for $COPSAC_{severe}$ and iPSYCH

Supplementary Figure 4. Comparing the observed risk score for *FUT2* and *ABO* with the additive model based risk score, and risk allele counts.

Supplementary Figure 5. *FUT2×ABO* genotype stratified heatmap of respiratory illnesses by *S. pneumoniae*

Supplementary Figure 6. $COPSAC_{severe}$ patient selection flow chart

Supplementary Figure 7. $COPSAC_{severe}$ and Inter99 genotype quality control (QC)

Supplementary Figure 8. iPsych study participant selection, genotyping and imputation QC

I. SUPPLEMENTARY NOTE 1

Study Cohorts and Phenotyping

Discovery Stage

COPSAC$_{severe}$[1]

This is a register-based cohort comprising children with asthma who have been characterized from the national health registries based on hospitalization for asthma. This study was approved by the Ethics Committee for Copenhagen (H-B-2998-103) and the Danish data protection agency (2008-41-2622). In accordance with the Danish law, the research ethics committee can grant exemption from obtaining informed consent under certain circumstances. For this study cohort, such an exemption was granted (H-B-2998-103).

Case Selection: Children with acute repeated hospitalizations where asthma was registered as the primary reason for hospitalization (cases) were identified in the Danish National Patient Register covering all diagnoses from discharges from Danish hospitals[2]. Information on birth-related events was obtained from the national birth register. Inclusion criteria were at least two acute hospitalizations for asthma (ICD8-codes 493, ICD-10 codes J45-46) from 2 to 6 years of age (both years included). Duration of hospitalization had to be more than 1 day, and two hospitalizations had to be separated by at least 6 months. Exclusion criteria were side diagnosis during hospitalization, registered chronic diagnosis considered to affect risk of hospitalization for asthma, low birth weight (<2.5 kg) or gestational age of under 36 weeks at birth. Cases were further characterized with respect to the number of hospitalizations

from asthma and acute bronchitis and for concurrent atopy. After all inclusion/exclusions 1204 cases of severe childhood asthma exacerbations were available. A flow chart describing the case selection process is available as **Supp. Figure 5.**

INTER99[3,4]

These control individuals were drawn from a middle aged population based Danish cohort called Inter99. Inter99 is a randomized, non-pharmacological intervention study for the prevention of ischaemic heart disease, conducted on 6,784 randomly ascertained participants aged 30 to 60 years at the Research Centre for Prevention and Health in Glostrup, Denmark (ClinicalTrials.gov: NCT00289237). Individuals who indicated in a questionnaire that they had physician-diagnosed asthma were excluded, and 5328 individuals from Inter99 participated as non-asthmatic controls in the current study.

iPSYCH[5]

The Integrative Psychiatric Research (iPSYCH) consortium has established a large Danish population-based Case-Cohort sample (iPSYCH2012) aimed at unravelling the genetic and environmental architecture of severe mental disorders. The iPSYCH2012 sample is nested within the entire Danish population born between 1981 and 2005, including 1 472 762 persons. Dried blood spots for virtually all individuals were retrieved from the Danish neonatal screening biobank and processed for genotyping and GWAS using DNA amplification method[6].

86189 individuals were included in the iPsych study, among which 77639 (90%) passed sample QC[7]. After all inclusions and exclusions, a total of 61,749 individuals of European ancestry (childhood asthma cases, n_{cases}: 1662 and non-asthmatic controls, $n_{controls}$: 60, 087) with genotype and phenotype data participated in the current study (**Supplementary figure 6**).

Cases (iPsych study):

Asthma cases within the iPSYCH study was defined using registry data from the National Patient registries. Individuals with at least one hospitalization with due to a primary diagnosis of asthma exacerbations (ICD8-codes 493, ICD-10 codes J45-46) in the first six years of life were classified as asthma cases. A total of 1662 asthma cases participated in the current study.

Controls (iPsych study):

The remaining 60, 087 individuals without hospitalizations due to severe asthma attacks were used a controls in the iPSYCH study.

Replication Stage
$COPSAC_{2000}$[8]

This is a mother child cohort where all the mothers had a history of a doctor's diagnosis of asthma after 7 years of age and thus this is a high risk asthma cohort comprising 411 children. Newborns were enrolled in the first month of life, and details on the cohort have been described previously.

COPSAC$_{2010}$[9]

This is a mother child cohort comprising of 700 children born to unselected mothers from Denmark and has been described previously in detail[9].

The Ethics Committee for Copenhagen and the Danish Data Protection Agency approved these two studies. The study was conducted in accordance with the guiding principles of the Declaration of Helsinki and was approved by the Local Ethics Committee (COPSAC$_{2000}$: KF 01-289/96, COPSAC$_{2010}$: H-B-2008-093), and the Danish Data Protection Agency (COPSAC$_{2000}$ and COPSAC$_{2010}$: 2015-41-3696). Both parents gave written informed consent before enrolment.

COAST study[10,11]

289 newborns were enrolled in Madison, Wisconsin, between November 1998 and May 2000, as described previously[10]. All the children had at least one parent with respiratory allergies, a history of physician-diagnosed asthma, or both. The parents of 214 of the newborns who were of European ancestry gave consent for their child to participate in genetic studies, and data from these children are included in the current analyses. A total of 200 of these children were evaluated for asthma beginning at 6 years of age. Current asthma was diagnosed at the end of the sixth year of life based on the documented presence of one or more of the following characteristics in the previous year: (1) physician diagnosis of asthma, (2) use of albuterol for coughing or wheezing episodes (prescribed by physician), (3) use of a daily controller medication, (4) step-up plan including use of albuterol or short-term use of inhaled corticosteroids during illness, and (5) use of prednisone for asthma exacerbation. Four separate investigators, blinded to any antecedent histories concerning viral

illnesses or patterns of aeroallergen sensitization, independently evaluated each subject for the presence or absence of asthma based on the above criteria.

RhinoGen[11]

The RhinoGen study included 167 outpatient children with asthma and 143 children without asthma aged 4 to 12 years, and was conducted to identify genetic and microbial associations with respiratory illnesses and exacerbations of asthma[11]. During peak cold seasons in April and September, samples of nasal mucus were collected weekly and 3028 specimens were analyzed using RT-PCR for common respiratory viruses and three bacterial pathogens (*S. pneumoniae*, *M. catarrhalis* and *H. influenzae*) as previously described[12,13]. Respiratory symptoms were recorded in diaries along with use of albuterol and peak expiratory flow rates. Written informed consent was obtained from the parents, and written assent was obtained from children aged 7 years and older. The study was approved by the University of Wisconsin Human Subjects Committee (H-2007-0136-CR008).

II. SUPPLEMENTARY TABLES

Supplementary Table 1. Study Characteristics

**Cohort**	**N (N_{cases}/$N_{controls}$)**	**Female %, $\%_{All}$ ($\%_{Cases}$/$\%_{Controls}$)**	**Cases**		
			**Age at asthma diagnosis (years)**	**% with exacerbations**	**[a]$N_{Exacerbations}$, Median (IQR)**
**Discovery Stage**					
COPSAC Severe	6532 (1204/5328)	47.6 (33.2/50.8)	2.2 (1.3 - 3.3)	100	4 (3 - 6)
iPSYCH	61,749 (1662/60,087)	47.0 (29.0/47.4)	1.8 (1.0 - 3.6)	100	1 (1 - 2)
**Replication Stage**					
COPSAC birth cohorts (2000 + 2010)	918 (191/727)	49.7 (43.7/51.3)	1.8 (1.4 - 2.5)	36.4	2 (1 - 4)

[a]Summary based on n=1188 cases with data available on number of exacerbations; Copsac $N_{exacerbation}$ is based on asthma cases with exacerbations (n=66/181)

Supplementary Table 2. Index SNP-asthma associations in the iPSYCH cohort stratified by psychiatric case-control status.

SNP (Genome-wide significant loci)	Psychiatric controls N = 18282 (311/17971)		Psychiatric cases N = 43467 (1351/42116)		Difference in effect estimates*
	OR (95% CI)	*P* value	OR (95% CI)	*P* value	
GSDMB rs7219923	1.27 (1.09-1.49)	0.002	1.49 (1.38-1.62)	8.00e-24	0.07
CDHR3 rs6967330	1.40 (1.16-1.70)	0.005	1.36 (1.24-1.49)	1.10e-10	0.75
HLA-DQA1 rs1071630	1.28 (1.08-1.51)	0.003	1.17 (1.08-1.27)	1.25e-04	0.28
IL33 rs340933	1.25 (0.99-1.59)	0.07	1.31 (1.17-1.31)	5.25e-06	0.65
IL33 rs1342326	1.30 (1.06 – 1.59)	0.01	1.22 (1.10 – 1.35)	9.14e-05	0.57
IL1RL1 rs10189629	1.37 (1.05-1.82)	0.02	1.36 (1.19-1.55)	7.68e-06	0.95
WDR36 rs1043828	1.14 (0.97-1.35)	0.10	1.18 (1.09-1.27)	4.80e-05	0.75
IL13 rs20541	1.13 (0.93-1.16)	0.21	1.15 (1.05-1.26)	0.003	0.85
FUT2 rs281379	1.15 (0.98-1.35)	0.09	1.19 (1.10-1.29)	1.22e-05	0.68

*P-value for the interaction between SNP and iPSYCH status in relation to childhood asthma. Logistic regression models adjusted for sex + 10 principal components. The reported P-values are not adjusted for multiple comparisons

Supplementary Table 3. Associations between index SNP and psychiatric disease status in the iPSYCH cohort.

SNP	Association with psychiatric disease status<br>N = 61749 (1662/60087)	
	**OR (95% CI)**	***P* value**
GWAS significant loci		
GSDMB rs7219923	1.00 (0.98-1.03)	0.97
CDHR3 rs6967330	0.97 (0.94-1.00)	0.05
HLA-DQA1 rs1071630	1.02 (0.99-1.05)	0.13
IL33 rs340933	1.00 (0.97-1.04)	0.65
IL33 rs1342326	1.00 (0.96 – 1.03)	1.00
IL1RL1 rs10189629	0.99 (0.96-1.03)	0.82
WDR36 rs1043828	0.99 (0.96-1.01)	0.39
IL13 rs20541	1.02 (0.99-1.06)	0.09
FUT2 rs281379	0.99 (0.97-1.02)	0.93

P-value for association between top SNPs and psychiatric status (presence/absence of psychiatric disorders) in in iPSYCH study. Logistic regression models adjusted for sex + 10 principal components. The reported P-values are not corrected for multiple comparisons.

Supplementary Table 4. Association results for the genome-wide significant loci ($p_{discovery} < 5.0 \times 10^{-8}$) in the discovery stage. Results are shown for the discovery stage as well as replication cohorts. The COPSAC birth cohorts results include a meta-analysis if COPSAC2000 and COPSAC2010). UK Biobank results represent analyses of asthma with onset during the first 6 years of life.

Chr	SNP	Discovery		COPSAC birth cohorts		UK Biobank	
		OR (95 CI%)	P-value	OR (95 CI%)	P-value	OR (95 CI%)	P-value
17	rs7219923	1.65 (1.56-1.75)	1.6×10^{-68}	1.62 (1.29-1.05)	4.0×10^{-5}	1.40 (1.34-1.45)	3.6×10^{-66}
7	rs6967330	1.41 (1.32-1.51)	2.1×10^{-23}	1.23 (0.91-1.65)	0.16	1.10 (1.04-1.15)	2.6×10^{-3}
6	rs1071630	1.25 (1.18-1.32)	8.0×10^{-14}	1.33 (1.07-1.67)	0.01	1.23 (1.17-1.27)	3.4×10^{-22}
9	rs340933	1.37 (1.26-1.49)	1.6×10^{-13}	1.24 (0.90-1.75)	0.20	1.27 (1.20-1.33)	2.5×10^{-18}
9	rs1342326	1.31 (1.22-1.40)	1.7×10^{-13}	1.16 (0.85-1.61)	0.38	1.29 (1.23-1.35)	3.3×10^{-26}
2	rs10189629	1.40 (1.27-1.54)	7.7×10^{-12}	1.50 (1.04-2.24)	0.04	1.40 (1.32-1.30)	3.9×10^{-26}
5	rs1043828	1.20 (1.14-1.27)	1.0×10^{-10}	1.09 (0.86-1.38)	0.46	1.15 (1.10-1.19)	2.0×10^{-12}
5	rs20541	1.21 (1.13-1.29)	1.0×10^{-8}	1.01 (0.76-1.34)	0.94	1.19 (1.13-1.24)	4.1×10^{-13}
19	rs281379	1.18 (1.11-1.25)	2.6×10^{-9}	1.38 (1.09-1.75)	8.4×10^{-3}	1.06 (1.02-1.10)	2.7×10^{-3}

Discovery: COPSACsevere n=6532 and iPSYCH n=61749, $N_{discovery}$=68281; COPSACcohorts: $N_{copsac\_cohorts}$=912; UK Biobank: $N_{UKBiobank}$=93978 (N_{cases}=5881, $N_{controls}$=88097); Chr:Chromosome; OR: Odds ratio; 95CI: 95% Confidence interval. Logistic regression models adjusted for sex and 10 principal components. The reported P-values are not corrected for multiple comparison.

Supplementary Table 5. Association of the *FUT2/MAMSTR* top SNP (rs281379) with childhood lung function, allergic sensitization, and eczema in COPSAC birth cohorts.

SNP (EA/OA)	Trait		BETA	SE	OR (95% CI)	*P*	N
rs281379 (G/A)	Infant lung function	FEF50	-0.0095	0.023	-	0.67	346
		FEV0.5	0.0028	0.016	-	0.86	350
		PD15	-0.189	0.112		0.09	306
	Lung function at 6 years	FEV1	-0.309	0.208	-	0.13	856
		FVC	0.02	0.014	-	0.15	865
		FEV1/FVC ratio	-0.209	0.136	-	0.12	854
	Allergic Sensitization		0.069	0.18	1.07 (0.75-1.53)	0.70	725
	Eczema/Atopic dermatitis		0.078	0.102	1.08 (0.88-1.32)	0.44	852

EA/OA: Effect allele/Other allele; LFT: Lung function test; OR: Odds ratio; CI: confidence interval. All tests have been adjusted for sex and cohort. Linear/Logistic regression models depending on continuous/binary variables. The reported P-values are not adjusted for multiple comparisons.

Supplementary Table 6. eQTL analyses for the top *FUT2/MAMSTR* SNP (rs281379) in relation to the genes in genomic proximity.

Gene	Beta estimate (95% CI)	P-value
FUT2	0.71 (0.66 ; 0.77)	2.05e-78
RASIP1	-0.42 (-0.52 ; -0.32)	3.47e-14
FAM83E	-0.06 (-0.12 ; -0.01)	0.02
RPL18	0.03 (0.00 ; 0.06)	0.02
SPHK2	0.03 (-0.04 ;0.11)	0.42
MAMSTR	-0.05 (-0.17 ; 0.07)	0.45
CA11	0.02 (-0.10 ; 0.15)	0.73
FUT1	0.01 (-0.07 ; 0.09)	0.79
DBP	0.01 (-0.06 ; 0.07)	0.82

These gene expression data are based on nasal epithelial cells from n=357 children from the $COPSAC_{2010}$ birth cohort, at their 6 years visit. Analysis is based on a linear regression. The reported P-values are not adjusted for multiple comparisons.

Supplementary Table 7. Variant effect predictor results for SNPs in the 99% credible set for the *FUT2/MAMSTR* locus.

SNP	Posterior probability	Consequence	Affected gene	Impact
rs281379	0.37	Downstream gene variant	*MAMSTR*	Modifier
rs503279	0.20	3’ UTR variant	*FUT2*	Modifier
rs504963	0.15	3’ UTR variant	*FUT2*	Modifier
rs485186	0.12	Synonymous variant	*FUT2*	Low
rs602662	0.09	Missense variant	*FUT2*	Moderate
rs601338	0.02	Stop gained	*FUT2*	High
rs2287921	0.01	Intron variant	*RASIP1*	Modifer
rs516246	0.01	Intron variant	*FUT2*	Modifer
rs2287922	0.009	Missense variant	*RASIP1*	Moderate

Supplementary Table 8. Combined annotation dependent depletion (CADD) scores for functional prediction of the *FUT2/MAMSTR* locus SNPs

SNP	Position (Base pairs)	Raw Score	PHRED (CADD)	SIFT prediction	PolyPhen2 prediction	Annotation	Gene
rs601338	49206674	9.18	48	damaging	Probably damaging	Stop Gain	*FUT2*
rs2287922	49232226	3.75	26.2	damaging	Probably damaging	Missense	*RASIP1*
rs602662	49206985	2.47	22.5	tolerated	Probably damaging	Missense	*FUT2*
rs2287921	49228272	0.298	7.19	-	-	Intron	*RASIP1*
rs281379	49214274	0.129	4.75	-	-	-	*MAMSTR/FUT2*
rs503279	49209010	-0.058	1.83	-	-	3'UTR	*FUT2*
rs504963	49208865	-0.018	2.35	-	-	3'UTR	*FUT2*
rs485186	49207206	-0.258	0.48	-	-	Upstream	*FUT2*
rs516246	49206172	-0.25	0.51	-	-	Intron	*FUT2*

SNP: Single Nucleotide Polymorphism; BP: SNP position in Base Pairs corresponding to dbSNP Assembly GRCh37.p13; SIFT: Sorting Intolerant from Tolerant (https://sift.bii.a-star.edu.sg); PolyPhen2 (Polymorphism Phenotyping v2; http://genetics.bwh.harvard.edu/pph2/dokuwiki/about).

Supplementary Table 9. *FUT2* (rs601338) and *ABO* interaction stratified by severity in $\text{COPSAC}_{\text{severe}}$ and iPSYCH

Disease stratum	$\text{COPSAC}_{\text{severe}}$			iPSYCH		
	Cases	OR [95% CI]	P	Cases	OR [95% CI]	P
**1**	-	-	-	1236	0.96 [0.85 ; 1.08]	0.48
**2**	276	1.06 [0.83 ; 1.36]	0.62	267	1.05 [0.82 ; 1.35]	0.70
**3**	240	1.24 [0.95 ; 1.62]	0.11	83	0.93 [0.60 ; 1.45]	0.76
**4-5**	293	1.26 [0.99 ; 1.62]	0.06	45	0.95 [0.52 ; 1.73]	0.87
**≥ 6**	279	1.51 [1.21 ; 1.88]	2.4e-04	31	1.56 [0.71 ; 3.47]	0.28

Disease stratum: number of exacerbations. Logistic regression models adjusted for sex and 10 principal components. The reported P-values are not adjusted for multiple comparisons.

Supplementary Table 10. Case-only analysis of the *FUT2* (rs601338) and *ABO* interaction stratified by severity in $COPSAC_{severe}$

**Disease stratum**	**$COPSAC_{severe}$**		
	**Cases**	**Beta [95% CI]**	**P**
**2**	276	0.01 [-0.13 ; 0.11]	0.80
**3**	240	0.07 [-0.04 ; 0.19]	0.24
**4-5**	293	0.07 [-0.04 ; 0.18]	0.21
**≥ 6**	279	0.17 [0.07 ; 0.27]	**9.2e-4**

Disease stratum: number of exacerbations. P values <0.05 in bold. Linear model associating the correlation between the two SNPs. The model is adjusted for 10 principal components and sex. Reported P-values are not adjusted for multiple comparisons.

Supplementary Table 11. Deriving the *FUT2(rs601338)-ABO* combined risk score. The score was applied for each individual in terms of the β-estimate according to genotype

COPSAC$_{severe}$			
**ABO [CC]**	β = -0.29, p = 0.69, N = 159 (24/135)	β = -0.04, p = 0.84, N = 431 (76/355)	β = 0.54, **p = 0.005**, N = 275 (71/204)
**ABO [CT]**	β = -0.29, p = 0.10, N = 612 (85/527)	β = 0.36, **p = 0.007**, N = 1537 (337/1200)	β = 0.34, **p = 0.009**, N = 909 (201/708)
**ABO [TT]**	Reference N = 518 (85/433)	β = -0.09, p = 0.52, N = 1268 (199/1069)	β = -0.13, p = 0.40, N = 823 (126/697)
	**FUT2 [AA]**	**FUT2 [AG]**	**FUT2 [GG]**

FUT2 SNP: rs601338, *ABO* SNP: rs505922. P values < 0.05 in bold. Logistic regression models adjusted for sex and 10 principal components. Reported P-values are not adjusted for multiple comparisons

Supplementary Table 12. Association of *FUT2(rs601338)-ABO* combined risk score with lung function, allergic sensitization, eczema and eosinophil counts in COPSAC birth cohorts

SNP/score	Trait		BETA	SE	OR (95CI)	P	N
FUT2-ABO combined risk score	Infant lung function	FEF50	-0.033	0.069	-	0.62	346
		FEV0.5	0.006	0.049	-	0.90	350
		PD15	-0.547	0.335		0.10	306
	Lung function at 6 years	FEV1	-0.547	0.335	-	0.10	306
		FVC	0.016	0.043	-	0.69	865
		FEV1/FVC ratio	-0.215	0.416	-	0.60	854
	Allergic Sensitization		0.851	0.55	2.34 (0.79-6.92)	0.12	725
	Eczema/Atopic dermatitis		0.53	0.308	1.69 (0.92-3.11)	0.08	852

FUT2 rs601338 and *ABO* rs505922 were used to calculate the combined risk score. LFT: Lung function test; SE: Standard error; OR: Odds ratio; CI: confidence interval. All tests have been adjusted for sex and cohort. Linear/Logistic regression models depending on continuous/binary variables. Reported P-values are not adjusted for multiple comparisons

Supplementary Table 13. Association between the *FUT2(rs601338)-ABO* combined risk score and number of bacterial infections ever in early life (0-3 years) in the combined COPSAC birth cohorts (COPSAC$_{2000}$ and COPSAC$_{2010}$)

**Infection type**	**No. Of Aspirates**	**Combined Risk**	
		**IRR (95% CI)**	***P* value**
**Bacteria**			
S. pneumoniae	473	2.31 (1.45 - 3.68)	**0.0004**
H. influenzae	445	1.15 (0.68 - 1.94)	0.59
M. catarrhalis	697	1.29 (0.84 - 1.89)	0.26
**Viruses**			
Rhinovirus	328	1.39 (0.80 - 2.40)	0.24
RSV	241	0.89 (0.51 - 1.54)	0.67
Corona	121	0.65 (0.28 - 1.50)	0.31
Parainfluenza	121	0.79 (0.37 - 1.70)	0.55
Influenza	78	0.58 (0.19 - 1.79)	0.34
Adeno	44	0.99 (0.27 - 3.65)	0.99
Metapneumo	61	0.88 (0.30 - 2.60)	0.82
Boca	74	1.16 (0.43 - 3.12)	0.76

IRR: Incidence Rate Ratio; CI: Confidence Interval. Quasi-Poisson regression model adjusted for sex and Cohort; P value <0.05 in bold. Reported P-values are not adjusted for multiple comparisons

III. SUPPLEMENTARY FIGURES

Supplementary Figure 1. Quantile-Quantile (Q-Q) Plot for the discovery stage GWAS meta-analysis

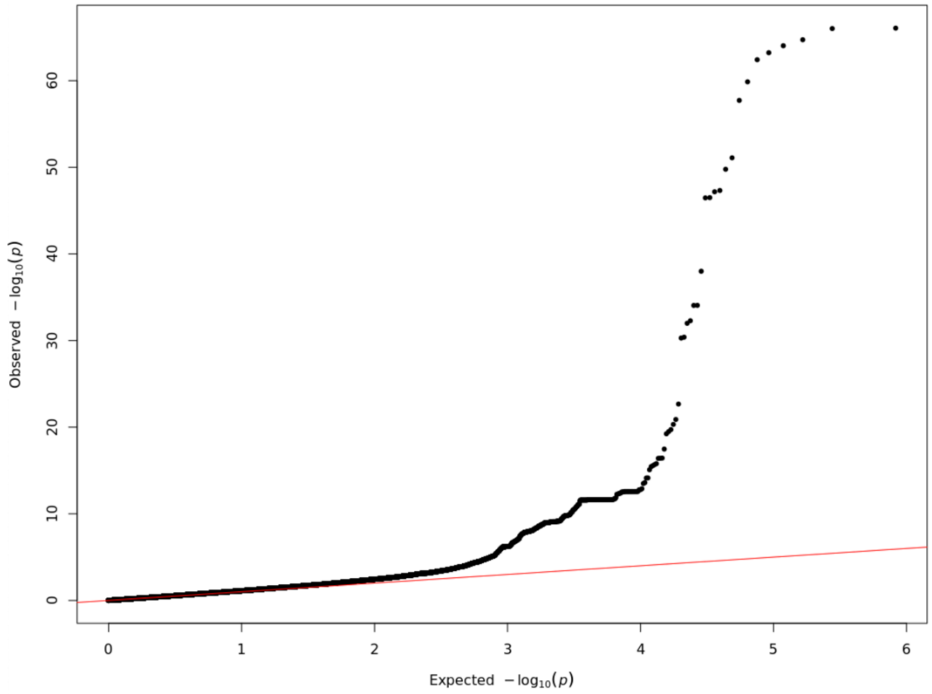

$\lambda_{\text{discovery meta}}$: 1.13, $\lambda_{\text{COPSACsevere}}$: 0.99, λ_{iPsych}: 1.02

Supplementary Figure 2. Regional Plot for the *FUT2*/*MAMSTR* locus rs281379 on chromosome 19. rs281379 (in purple diamond shaped) is the most significant SNP in the region, based on association P value; all other SNPs are shown in circles. The colors represent the degree of linkage disequilibrium (LD) with the index SNP (r^2 values in the key); For additional documentation please see http://locuszoom.sph.umich.edu//

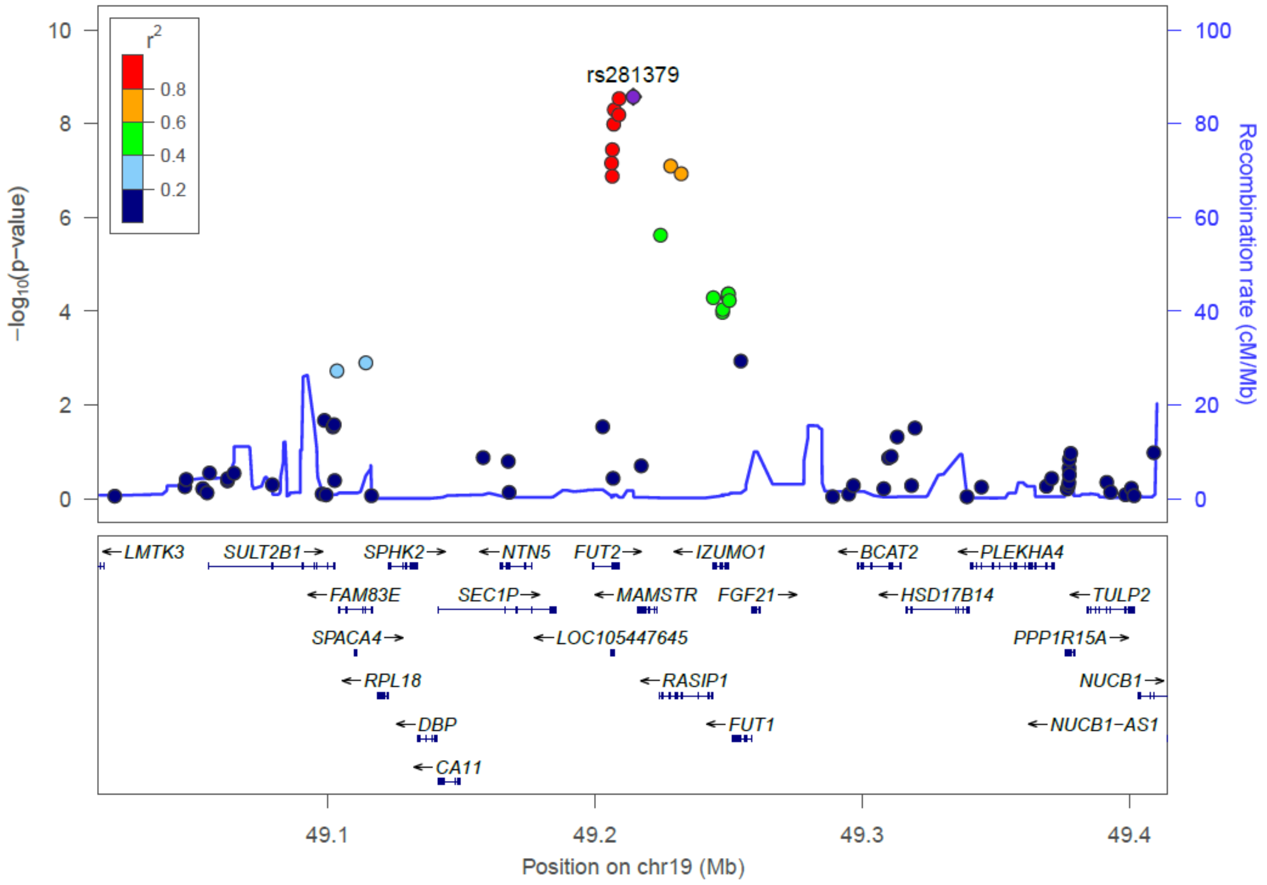

Supplementary Figure 3. Manhattan plots for A) $COPSAC_{severe}$ and B) iPSYCH. Association P-value (suggestive threshold line in black): $1{\times}10^{-4}$, p value (genome-wide threshold line in red): 5.0×10^{-8}. Associations based on logistic regression. Model adjusted for sex, and first 10 principal components. $N_{Copsac\ Severe}$ = 6,532. N_{iPsych} = 61,749.

A) $COPSAC_{severe}$

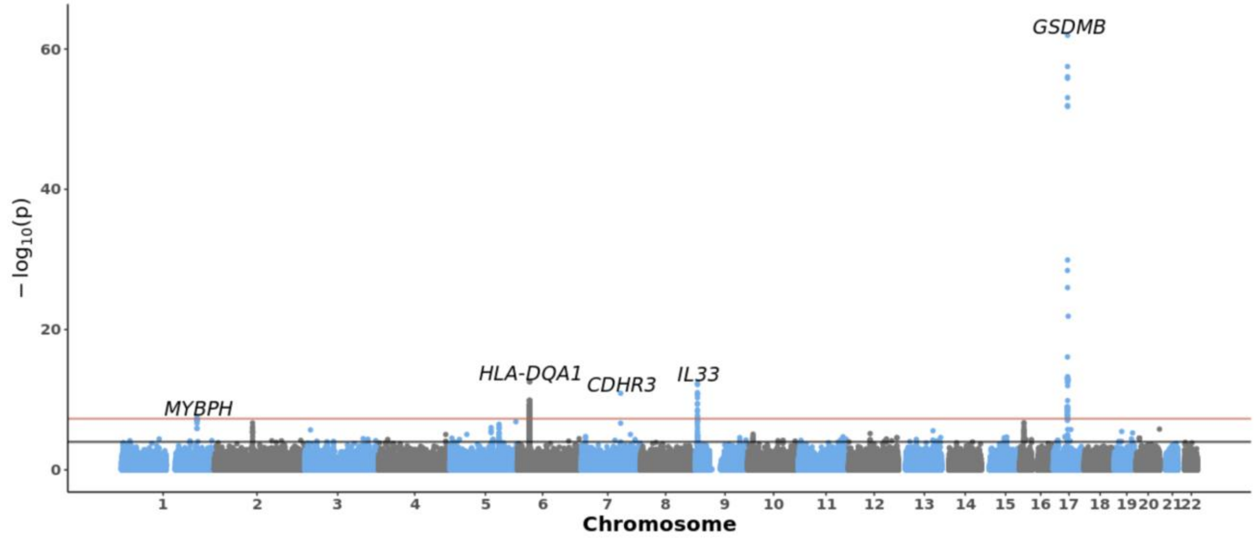

B) iPSYCH

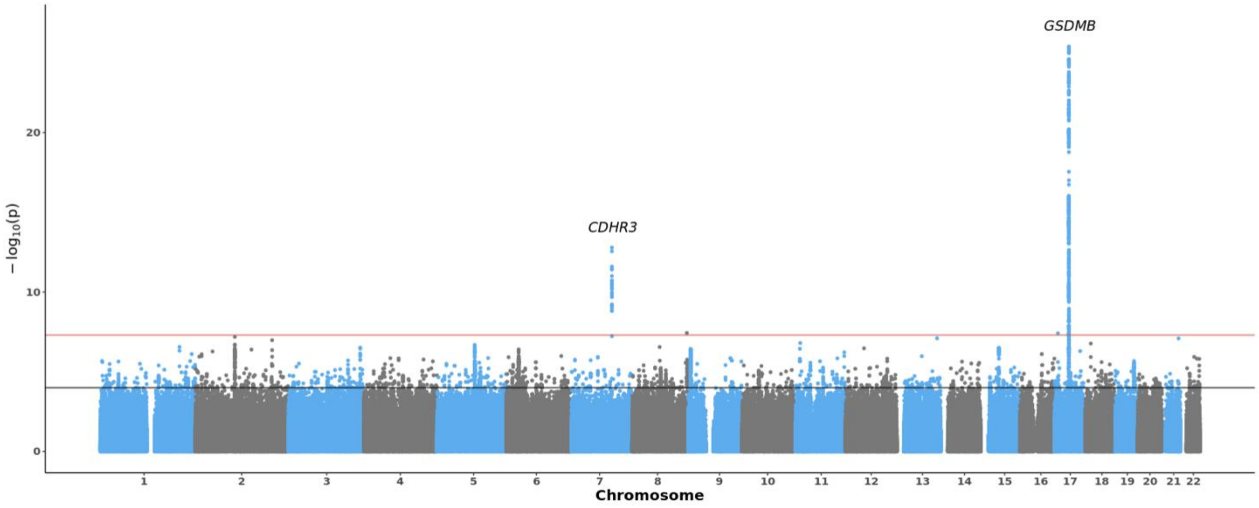

GSDMB
CDHR3
20
10
0
− log10(p)
1
2
3
4
5
6
7
8
9
10
11
12
13
14
15
16
17
18
19
20
21
22
Chromosome

Supplementary Figure 4. Comparing the observed risk score for *FUT2(rs601338)* and *ABO* with the additive model-based risk score, and risk allele counts. The observed risk score is based on per-genotype estimates from a logistic regression model. Additive model risk is based on the overall estimated effects using a logistic regression. Risk-allele counts represents the number of risk alleles for the two SNPs.

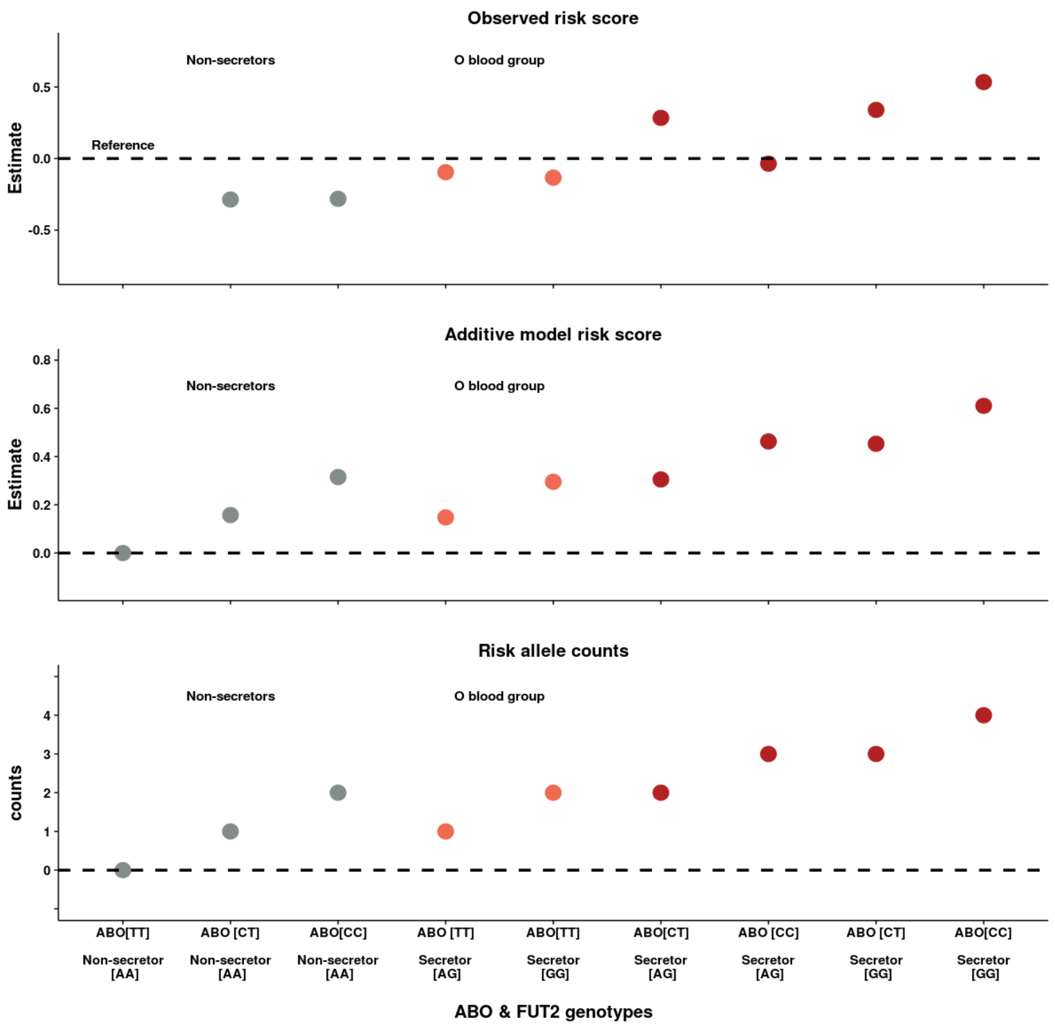

Supplementary Figure 5. *FUT2(rs601338)-ABO* genotype stratified heatmap of respiratory illnesses with *S. pneumoniae*. Incidence rate ratios are estimated using a quasi-Poison model adjusted for sex and cohort status. The reported P-values are not adjusted for multiple comparisons.

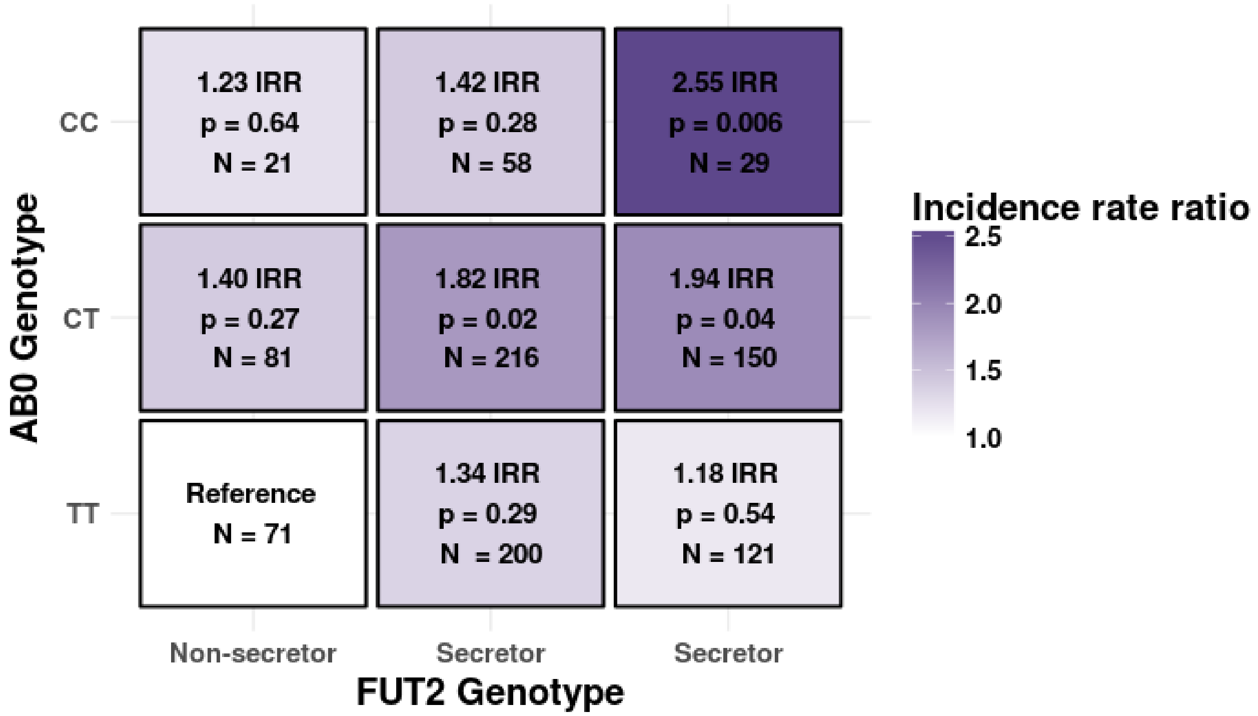

FUT2 SNP: rs601338, *ABO* SNP: rs505922

Supplementary Figure 6. COPSAC$_{severe}$ case selection flow chart

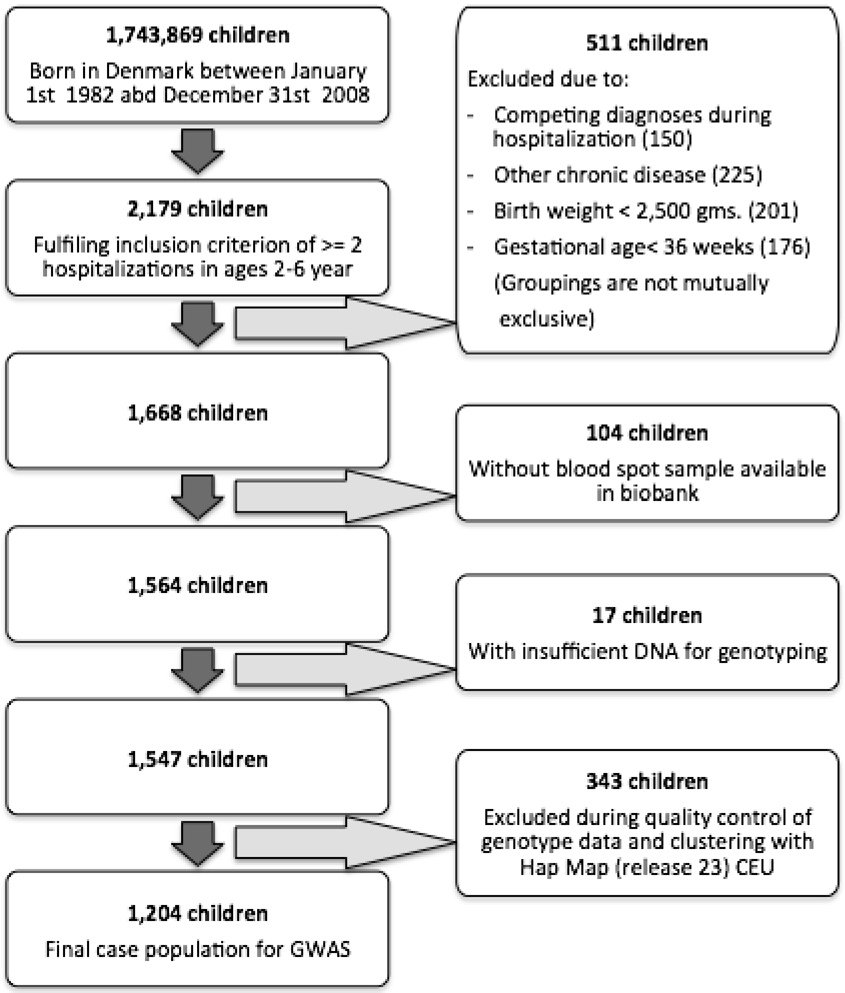

Supplementary Figure 7. COPSAC$_{severe}$ and Inter99 genotype quality control (QC).

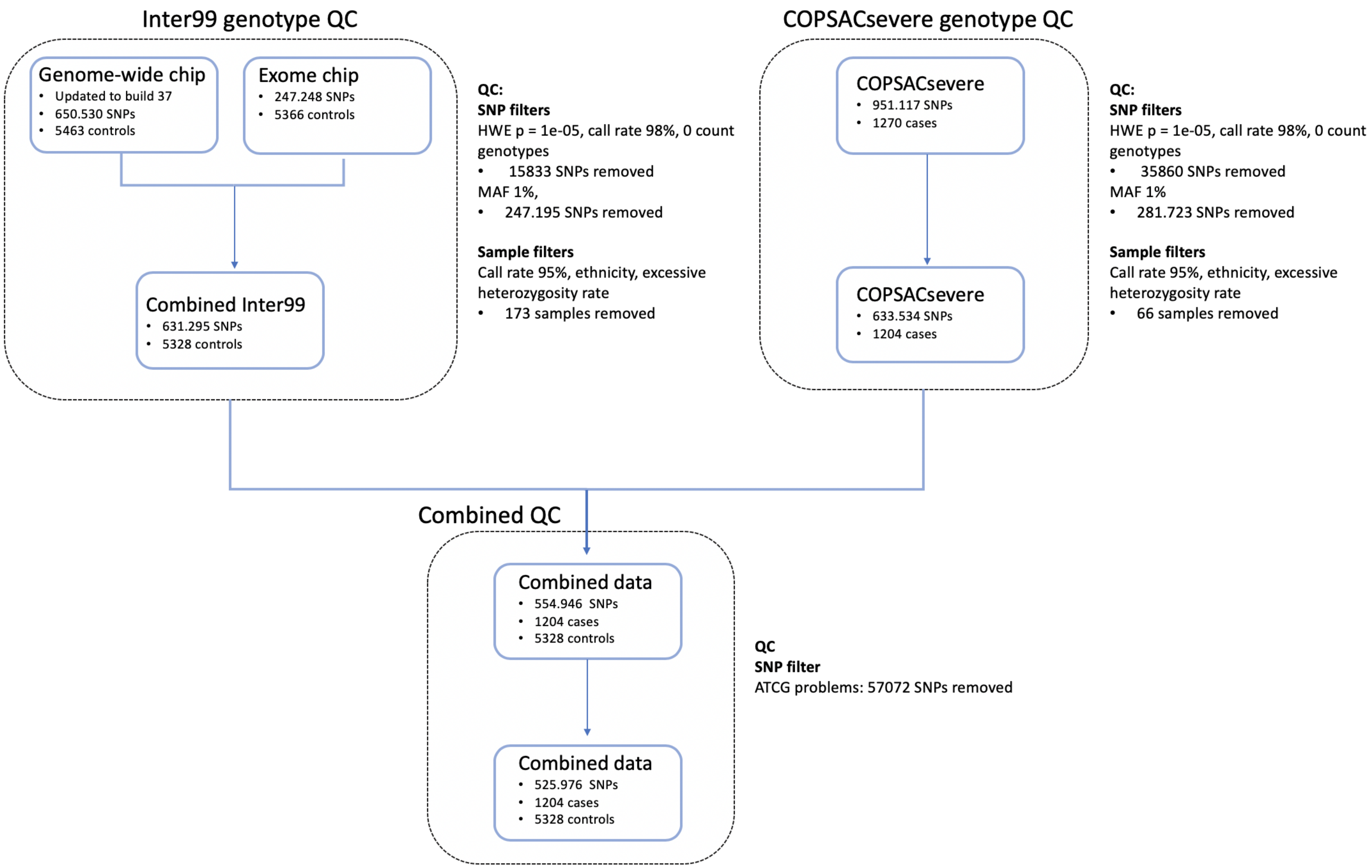

Supplementary Figure 8. iPSYCH study participant selection, genotyping and imputation quality control (QC) flow chart.

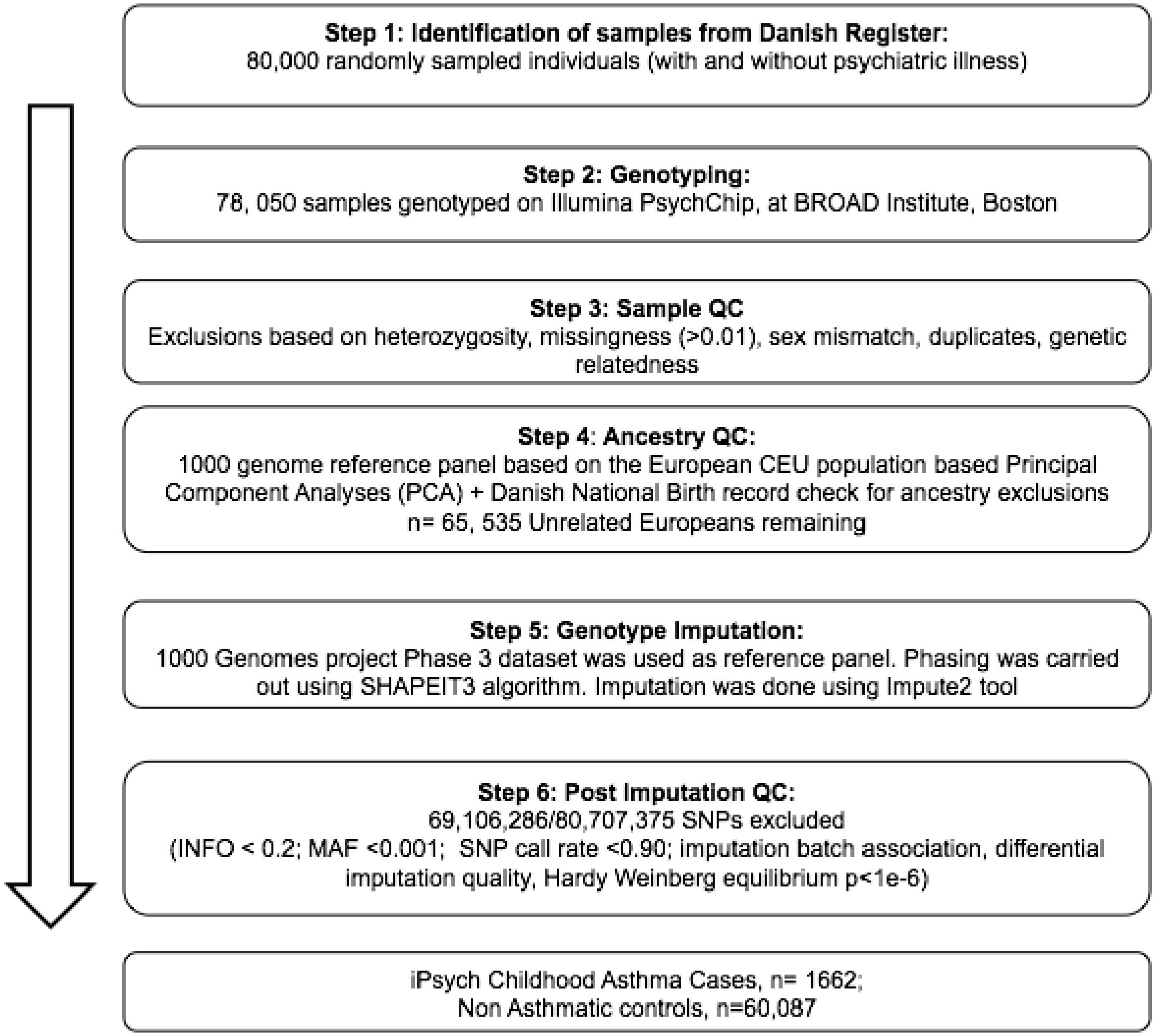

Supplementary References

1. Bonnelykke, K. *et al.* A genome-wide association study identifies CDHR3 as a susceptibility locus for early childhood asthma with severe exacerbations. *Nat Genet* **46**, 51-5 (2014).
2. Lynge, E., Sandegaard, J.L. & Rebolj, M. The Danish National Patient Register. *Scand J Public Health* **39**, 30-3 (2011).
3. Ahluwalia, T.S. *et al.* A novel rare CUBN variant and three additional genes identified in Europeans with and without diabetes: results from an exome-wide association study of albuminuria. *Diabetologia* **62**, 292-305 (2019).
4. Jorgensen, T. *et al.* A randomized non-pharmacological intervention study for prevention of ischaemic heart disease: baseline results Inter99. *Eur J Cardiovasc Prev Rehabil* **10**, 377-86 (2003).
5. Demontis, D. *et al.* Discovery of the first genome-wide significant risk loci for attention deficit/hyperactivity disorder. *Nat Genet* **51**, 63-75 (2019).
6. Hollegaard, M.V. *et al.* Robustness of genome-wide scanning using archived dried blood spot samples as a DNA source. *BMC Genet* **12**, 58 (2011).
7. Pedersen, C.B. *et al.* The iPSYCH2012 case-cohort sample: new directions for unravelling genetic and environmental architectures of severe mental disorders. *Mol Psychiatry* **23**, 6-14 (2018).
8. Bisgaard, H. The Copenhagen Prospective Study on Asthma in Childhood (COPSAC): design, rationale, and baseline data from a longitudinal birth cohort study. *Ann Allergy Asthma Immunol* **93**, 381-9 (2004).
9. Bisgaard, H. *et al.* Deep phenotyping of the unselected COPSAC2010 birth cohort study. *Clin Exp Allergy* **43**, 1384-94 (2013).
10. Caliskan, M. *et al.* Rhinovirus wheezing illness and genetic risk of childhood-onset asthma. *N Engl J Med* **368**, 1398-407 (2013).
11. Loisel, D.A. *et al.* Genetic associations with viral respiratory illnesses and asthma control in children. *Clin Exp Allergy* **46**, 112-24 (2016).
12. Kloepfer, K.M. *et al.* Detection of pathogenic bacteria during rhinovirus infection is associated with increased respiratory symptoms and asthma exacerbations. *J Allergy Clin Immunol* **133**, 1301-7, 1307 e1-3 (2014).
13. Bashir, H. *et al.* Association of rhinovirus species with common cold and asthma symptoms and bacterial pathogens. *J Allergy Clin Immunol* **141**, 822-824 e9 (2018).
