# Supplementary material for: FUT2–ABO epistasis increases the risk of early childhood asthma and Streptococcus pneumoniae respiratory illnesses
Source: Nat Commun. 2020 Dec 16;11:6398. doi: 10.1038/s41467-020-19814-6 (PMC7744576; doi:10.1038/s41467-020-19814-6)
Supplement: Supplementary file 3 — Reporting Summary [file 41467_2020_19814_MOESM3_ESM.pdf]

nature research

Corresponding author(s): Klaus Bønnelykke

☐ Initial submission ☐ Revised version ☒ Final submission

Life Sciences Reporting Summary

Nature Research wishes to improve the reproducibility of the work that we publish. This form is intended for publication with all accepted life science papers and provides structure for consistency and transparency in reporting. Every life science submission will use this form; some list items might not apply to an individual manuscript, but all fields must be completed for clarity.

For further information on the points included in this form, see Reporting Life Sciences Research. For further information on Nature Research policies, including our data availability policy, see Authors & Referees and the Editorial Policy Checklist.

› Experimental design

1. Sample size

Describe how sample size was determined.	We included available data-sets on early childhood asthma hospitalization in order to provide optimal power for GWAS discovery. This resulted in a sample size that was several times larger than previous GWAS- studies on this phenotype, which was sufficent for providing genome-wide significant findings.

2. Data exclusions

Describe any data exclusions.	Described at individual data steps.

3. Replication

Describe whether the experimental findings were reliably reproduced.	Replication steps were included both for the initial asthma susceptibility locus as well as for the association with specific infectious triggers, which both provided successful replication.

4. Randomization

Describe how samples/organisms/participants were allocated into experimental groups.	No randomization took place in this study. Cases and controls were analyzed based upon predefined criteria and analyzed according to genotype.

5. Blinding

Describe whether the investigators were blinded to group allocation during data collection and/or analysis.	Doctors characterizing cases and controls were blinded to child genotypes.

Note: all studies involving animals and/or human research participants must disclose whether blinding and randomization were used.

6. Statistical parameters

For all figures and tables that use statistical methods, confirm that the following items are present in relevant figure legends (or in the Methods section if additional space is needed).

n/a	Confirmed	
☐	☒	The exact sample size (*n*) for each experimental group/condition, given as a discrete number and unit of measurement (animals, litters, cultures, etc.)
☐	☒	A description of how samples were collected, noting whether measurements were taken from distinct samples or whether the same sample was measured repeatedly
☒	☐	A statement indicating how many times each experiment was replicated
☐	☒	The statistical test(s) used and whether they are one- or two-sided (note: only common tests should be described solely by name; more complex techniques should be described in the Methods section)
☐	☒	A description of any assumptions or corrections, such as an adjustment for multiple comparisons
☐	☒	The test results (e.g. *P* values) given as exact values whenever possible and with confidence intervals noted
☐	☒	A clear description of statistics including central tendency (e.g. median, mean) and variation (e.g. standard deviation, interquartile range)
☐	☒	Clearly defined error bars

See the web collection on statistics for biologists for further resources and guidance.

Software

Policy information about availability of computer code

7. Software

Describe the software used to analyze the data in this study.	The single SNP analysis was done using PLINK v.1.9, and the meta-analysis for the discovery phase was conducted using the software METAL (version March 2011) with an inverse variance weighted fixed-effect model. Downstream data analysis was done using R v. 3.4.4. In addition, the following software and versions were used:<br>-PolyPhen2 (v2, web interface)<br>-CADD v1.6<br>- STAR v2.5.1a

For manuscripts utilizing custom algorithms or software that are central to the paper but not yet described in the published literature, software must be made available to editors and reviewers upon request. We strongly encourage code deposition in a community repository (e.g. GitHub). *Nature Methods* guidance for providing algorithms and software for publication provides further information on this topic.

Materials and reagents

Policy information about availability of materials

8. Materials availability

Indicate whether there are restrictions on availability of unique materials or if these materials are only available for distribution by a for-profit company.	No unique materials were used.

9. Antibodies

Describe the antibodies used and how they were validated for use in the system under study (i.e. assay and species).	No antibodies were used.

10. Eukaryotic cell lines

a. State the source of each eukaryotic cell line used.	No eukaryotic cell lines were used.
b. Describe the method of cell line authentication used.	No eukaryotic cell lines were used.
c. Report whether the cell lines were tested for mycoplasma contamination.	No eukaryotic cell lines were used.
d. If any of the cell lines used are listed in the database of commonly misidentified cell lines maintained by ICLAC, provide a scientific rationale for their use.	No eukaryotic cell lines were used.

Animals and human research participants

Policy information about studies involving animals; when reporting animal research, follow the ARRIVE guidelines

11. Description of research animals

Provide details on animals and/or animal-derived materials used in the study.	No animals were used.

Policy information about studies involving human research participants

12. Description of human research participants

Describe the covariate-relevant population characteristics of the human research participants.	Information on research participants in the individual studies is provided in the methods and supplementary material.<br>All human research was approved by the relevant institutional review boards and ethical committees and conducted according to the Declaration of Helsinki. All participants and/or their parents in the clinical studies provided written and oral informed consent. COPSAC2000, COPSAC2010, and Inter99 were approved by the Danish Scientific Ethics Committee, Region H. The registry-based studies (COPSACsevere and iPSYCH) were approved by the Danish Scientific Ethics Committees (Region H and Region Midt, respectively), the Danish Health Data Authority, the Danish data protection agency, and the Danish Neonatal Screening Biobank Steering Committee.
