# Supplementary material for: FUT2–ABO epistasis increases the risk of early childhood asthma and Streptococcus pneumoniae respiratory illnesses
Source: Nat Commun. 2020 Dec 16;11:6398. doi: 10.1038/s41467-020-19814-6 (PMC7744576; doi:10.1038/s41467-020-19814-6)
Supplement: Supplementary file 4 — Description of Additional Supplementary Files [file 41467_2020_19814_MOESM4_ESM.pdf]

Description of Additional Supplementary Files

Supplementary Data 1
All genome-wide significant SNPs from the discovery GWAS meta-analysis.

Supplementary Data 2
Severity stratification based on the number of hospitalizations for COPSACsevere and iPSYCH.

Supplementary Data 3
FUT2 and *ABO* main and stratified effects for each severity stratum in COPSACsevere
