# Supplementary material for: FUT2–ABO epistasis increases the risk of early childhood asthma and Streptococcus pneumoniae respiratory illnesses
Source: Nat Commun. 2020 Dec 16;11:6398. doi: 10.1038/s41467-020-19814-6 (PMC7744576; doi:10.1038/s41467-020-19814-6)
Supplement: Supplementary file 2 — Peer Review File [file 41467_2020_19814_MOESM2_ESM.pdf]

Reviewers' comments:

Reviewer #1 (Remarks to the Author):

In this paper, Ahluwalia et al study the genetic architecture of asthma with exacerbation in children of European ancestry. They perform exome-wide association study in two independent cohorts followed by meta-analysis (N = ~3000 pediatric cases, ~ 65000 controls) to identify common variants (MAF >= 1) associated with childhood asthma. In their meta-analysis, they report six loci that reach the genome-wide significance including one novel locus between FUT2 and MAMSRT genes on chromosome 19. They replicated the results in an independent cohort of ~1000 children and find a similar effect size for the index variant at FUT2/MAMSRT locus in the replication cohort. The authors then test the interaction of a nonsense variant that is in LD with the index SNP at FUT2/MAMSRT locus and an intronic variant overlapping ABO and find a significant interaction between these two loci in the most severe asthma cases, concluding that the underlying mechanisms driving the FUT2/MAMSRT association signal is related to secretion of ABO antigens. Finally, the authors check the association between a risk score, generated using the FUT2/ABO interacting alleles, and different respiratory infections. They show that this risk score is associated with S. pneumoniae infection in the pediatric population. The points raised by the authors about the importance of careful phenotyping in the study of asthma (and other complex phenotypes) as well as the importance of considering epistatic effects in understanding the genotype-phenotype relationships in genetic studies of complex traits is certainly true. The challenge that this reviewer had with the manuscript is that there felt that the narrative is built upon a single lead (association at FUT2/MAMSRT locus) without sufficient evidence for causality for asthma. I felt that the analysis presented provided weak evidence for the conclusions made by the authors and in places they had issues that I could not get over right away.

Major Comments:
1- Meta-analysis of Exome-wide association analyses lead to the discovery of six association signals (p < 5x10-8) with replication in an independent cohort. I had a number of concerns/questions regarding the association signals:
A. The references for the five previously known association signals are not given. Furthermore, it is important to know if the OR from the current study is similar (in terms of direction and magnitude of effect) with the results from independent studies. And if not what can derive the difference.
B. Can you add the replication and meta-analysis results (OR and P) for the five previously known SNPs to table 1?
C. The authors claim the larger effect sizes in the COPSACsevere reflect the more severe phenotype in this cohort. Given the overlap in the confidence intervals of ORs for the two cohorts are the differences between ORs significant? Does including the number of hospitalizations (as a proxy for severity) makes the ORs in the two cohorts to converge?
D. I checked the recent study of childhood asthma from UK biobank (Ferreira, et al, AJHG, 2019) and the FUT2/MAMSRT signal is not detected in this (larger) cohort can the authors comment on this?
E. Given the difference in age distribution for the two discovery cohorts (Table S1), the large difference age between cases and controls, and the difference in asthma risk between age groups (see Ferreira, et al, AJHG, 2019) it seems to me that age is an appropriate covariate to include in the model. Does the inclusion of the age affect the association results (strength and significance)?
F. Figure S1 shows that the lambda is 1.11 for the meta-analysis. Can the authors comment on the inflation of lambda? Could the inflation of test statistics reflect the lack of adequate correction for age or other confounders?
G. In figure 2 the signal at CDHR3 and IL33 looks unusual to me, the expected LD tail is not present for these two signals that raise concerns regarding false positives. Can the authors comment on this?
H. The authors mention that a random effect model was also applied at the discovery stage what are the fixed and random effects included in this model? If the random effect is the individual is this because there are overlaps between the two discovery cohorts? The p-values for the random effect model are largely different from the meta-analysis and logistic regression p-values why is that? What

are the ORs for the random effect?

2- Replication cohort included 185 pediatric cases and 787 controls from COPSAC 2010 cohort plus an additional 411 (healthy?) children from COPSAC 2000 cohort. My concerns regarding the replications analysis are:
A. I had a hard time understanding why the authors did the replication analysis using a Cox model instead of logistic regression. Yes, the cohort designs are different and COPSAC 2000 have probably higher asthma risk than average controls but what is the replication the ORs and p-values if you do perform a case-control analysis? What are the ORs and p-values in the COPSAC 2010 only (are the p-values nominally significant? Are the ORs similar to the discovery cohort?)
B. What are the frequencies of tag SNPs in COPSAC 2000 and COPSAC 2010?
C. Are the HR from the Cox model and the ORs from logistic regression/meta-analysis really comparable? I frankly don't know. Can you comment?

3- The authors use "SNP clumping" to identify the index SNPs at each locus followed by conditional analysis to identify independent signals within the locus. Why identifying the causal SNPs at a locus with highly linked variants is challenging the standard practice is to perform fine-mapping to narrow down the association signal at the FUT2/ MAMSRT locus as the SNP with the lowest p-value might not be the SNP with the highest likelihood to derive the association signal. For further information on fine-mapping see Schaid et al Nat Rew Gen, 2018.

4- While a causal SNP is not convincingly identified, possible causal genes are given even less attention, thus limiting the impact of the manuscript in understanding childhood asthma pathogenesis.
A. The authors assume that the signal at rs281379 is mapped to the FUT2 gene. The nearest genes to rs281379 are MAMSRT (~2 Kb) and FUT2 (~ 5Kb). However, as shown in figure S2, several other genes exist in the region. What is the justification for choosing FUT2 for further follow up (interaction analysis, risk of infection)?
B. Is it possible that rs281379 or other SNPs in linkage with it have a regulatory effect on FUT2 or other genes in the region (expression, splicing, methylation, etc)? Is there any evidence of physical interaction with nearby genes?
C. After narrowing the causal SNP and causal gene using in silico/statistical methods, bringing functional validation of the results can bring strong orthogonal support for the author's observations.

5- After the association analysis, the authors move on to investigate the interaction between the index SNP at FUT2/MAMSRT locus and ABO locus based on the known biological relationship between these two loci. I find the results of analysis speculative and unconvincing due to the following reasons:
A. The interaction is done between a nonsense SNP overlapping FUT2 and the top association signal overlapping ABO which is an intronic SNP. The FUT2 nonsense SNP is in LD with the index SNP at FUT2/MAMSRT locus ($r2 = 0.8$) whoever given the large difference between the MAF of these two alleles (0.23 vs. 0.32) the LD is probably not strong enough to assume that FUT2/MAMSRT index SNP is tagging this SNP. To me, the biological relevance is not enough to justify this choice of FUT2 nonsense SNP as causal SNP.
B. Moreover, neither the nonsense FUT2 SNPs nor the ABO intronic SNP reaches the genome-wide significance. This combined with the fact that the interaction p-values are not very significant (only interactions in the 6+ hospitalization group pass multiple testing threshold, table S6) makes me wonder if the observed effect can be driven by chance (or by a biological mechanism unrelated to the secretion of ABO antigens). If you take all variants that are similarly in LD ($r2 >= 0.8$) with the index SNP at the FUT2/ MAMSRT locus and all the variants in LD with the index SNP at ABO locus how often do one see p-values as strong as the ones in obtained by the interaction analysis? How often does one see a similar or larger OR? And how often do one see the effect getting stronger in the more severe group (6+ hospitalization)? If most of the interactions that are less biological plausibility have a more significant p-value/OR, or a stronger OR in the severe subset of patients what does that mean for the proposed biological mechanism (e.g effect of a FUT2 nonsense variant on ABO the secretor/non-secretor status).

C. Can you repeat the interaction using case-only analysis comparing (sever to non-sever cases), what is the OR and p-vale in this analysis? Having the same magnitude and direction of effect in this independent comparison can bring supporting evidence for claims regarding the role of this FUT2xABO interaction in asthma severity.
D. The conclusion from the interaction analysis states "since it (the interaction) is biologically plausible, it supports the validity of the two individual association signals, including the ABO locus which did not reach genome-wide significance." This is a dangerous claim to make! Biologically plausible associations can be driven by chance and without a biological basis as proven by many years of candidate gene studies.

Minor comments:

1. In the replication cohort, the numbers reported for in table S1 are 185 cases and 727 controls. The number of controls doesn't match the number of controls presented in the methods (787+411 != 727) where does the discrepancy come from?
2. What is the threshold for removing related individuals?
3. How the IBD calculation and genotyping QC is done? I assume using plink however no reference was provided.
4. Can you add the median age of controls to table S1? Can you report the information for the two replication cohorts separately in the table?
5. Methods are sparsely reported, examples: heterogeneity P.value calculation, random effect model for discovery stage, covariates included in the interaction model.
6. Can you add a supplementary table for all the variants the passes the genome-wide significance threshold at each of the six loci?
7. Are the risk rations presented in figure 3 statistically different?

Reviewer #2 (Remarks to the Author):

Ahluwalia and co workers present the results of the exome wide analysis of exomic variation in relation to hospitalizations due to asthma before the age of 6 years in two cohorts (COPSACsevere and IPsych), and replicate their findings in two well-characterized birth cohorts from the COPSAC group for asthma. In COPSAC severe and the combined COPSAC sample, but not in IPsych, they present replicable interactions between a novel reported variant in the FUT2 locus and the ABO locus. They go on to show that the risk score is related to respiratory illness, in relation to Str Pneumonia carriage, a finding that is subsequently replicated in an additional cohort.
This manuscript contains several important and interesting findings: A novel locus for early childhood exacerbations, evidence for epistasis in the genetic susceptibility for asthma exacerbations and recurrent infections, as well as a biologically plausible hypothesis for the role of FUT2 ABO interactions. This finding, if replicated, may have important consequences for future attempts of preventing asthma exacerbations in vulnerable subgroups. This paper is well written, statistical methods are sound.

There are also several concerns relating to the presentation of this work, and the analysis shown, that will be outlined below.

MAJOR CONCERNS

1. The two discovery cohorts. Phenotype definition from the two discovery cohorts (COPSAC severe and I Psych) was done based on registry data. However, the phenotype defined was different: 2 or more exacerbations in COPSAC severe, and One or more exacerbations in I Psych. Inconsistencies between the cohorts are interpreted due two these different definitions, but is unclear why the

phenotype definition was different?
a. Please present the I Psych data stratified by the number of exacerbations (these data are available, according to the supplement).
b. In table 1, please present cohort specific results, as well the meta analysis, using both fixed and random effects.
c. For the control selection in I Psych: were subjects with asthma or exacerbations > 6 years included as controls?

2. SNP selection for the exome wide study of COPSACsevere. The number of SNPs in the discovery cohort is drastically reduced due to the study design (Study 1 263 K SNPs, Study 2 951 K SNPs, overlap only 32 K SNPs.
a. Could a flow chart be provided how that is possible?
b. Could the authors comment how well this remaining SNPs set covers the exome?

3. Reference of this locus as an (childhood) asthma locus. While this reviewer has no doubt that the findings done in the discovery phase are done for a highly relevant phenotype (hospitalizations labeled as asthma exacerbations), describing a group of children that have severe episodes of dyspnea and bronchoconstriction that necessitates a hospitalization, the further work on this locus needs careful description of the phenotype. Replication was shown for common cold symptoms as well
a. Please provide a detailed description of the asthma definition in COPSAC. Which symptoms were used for this definition? " certain burden of symptoms" is unclear.
b. Perhaps, describing the 2 – 6 year definition as asthma symptoms is a better way forward (as was previously done in COPSAC as troublesome respiratory symptoms).
c. In the RhinGen study, was this locus associated with asthma using their definitions? This may help understand the reader a bit more what the phenotypic correlates of this locus are.

MINOR CONCERNS
4. Ascertainment of the cohorts. Are I Psych and Inter99 truly randomly ascertained population based cohorts? Or is any case selection present? This was well presented for I Psych, but is unclear for Inter99
5. R155. A functional SNP is proposed for the FUT2 locus.
a. Is this the only functional SNP or eQTL for the FUT2 top SNP? In the discussion, it is proposed that the FUT2 signal could be due to nearby genes, but why ?
b. Please present exact r2.
6. The interaction of the FUT2 locus and the ABO was not significant in the discovery dataset. Please provide full data on population specific interactions for the 2 cohorts in the supplement , not only for the COPSACsevere but also for the I Psych, stratified on the number of exacerbations.
7. R218 " Current asthma" ; how defined?
8. R239 upper respiratory symptoms: which symptoms
9. Discussions, when discussing previous evidence from other studies on the ABO locus (line 289 – 295), please add sample size and phenotype definition of these study to the discussion.
10. Table 3. Please specify for which phenotype the interactions are shown
11. Figure 6, two different Y axis: Rate of infections and Symptoms burden are shown, please explain the differences

Copenhagen, May 25th, 2020

To the Editors and reviewers

We thank the reviewers for thorough evaluation of our study and valuable suggestions for improvements, which we address individually below. We believe that we have been able to address all the issues raised, and this has significantly improved the value of our study. The improvements include a significant increase in the genome-wide coverage from 32.669 to 525.976 SNPs from adding additional genotyping data. In addition, we have clarified that the epistasis finding was not due to multiple testing and have further studied the biologically plausible SNPs involved by fine mapping analyses.
We believe that the findings of epistasis related to a specific asthma phenotype will be of interest to a broad readership and hope that you will now find it suitable for publication in Nature Communications.

Sincerely,
Tarun Ahluwalia, Anders Eliasen and Klaus Bønnelykke

Response to the reviewer's comments

Reviewer #1

1 - Meta-analysis of Exome-wide association analyses lead to the discovery of six association signals ($p < 5\times10^{-8}$) with replication in an independent cohort. I had a number of concerns/questions regarding the association signals:

A. The references for the five previously known association signals are not given. Furthermore, it is important to know if the OR from the current study is similar (in terms

of direction and magnitude of effect) with the results from independent studies. And if not what can derive the difference.

R1. As per the reviewer's suggestions, references for the known genome-wide significant loci have been added to Table 1.
The effect directionality is in concordance with previous reports for all loci.
The effect sizes in our study are generally larger in magnitude compared to previous reports focusing on more heterogenous asthma phenotypes (e.g. Moffatt et al. 2010, and Demenais et al. 2018). This is probably due to the more homogenous and severe phenotype in our study, as discussed in detail in R4 and shown in the new Supplementary Table 2. As shown in **R4**, the effect sizes were consistently lower in the stratum with few hospitalizations (1 or 2 hospitalizations) where it was comparable to other independent studies using a broader phenotype of asthma (ORs generally in the range of 1.1 to 1.3 for childhood asthma), while it increased to substantially larger effect sizes for children with many hospitalizations. The larger effect sizes are probably also partly explained by the focus on early childhood asthma, since earlier onset is associated with increased heritability (Thomsen et al. 2010) and correspondingly higher effect sizes (Pividori et al. 2019).
As an example, the top signal in our study (and most other GWAS on asthma) was the 17q21 locus. This locus had an OR of 1.41 in a recent study of childhood asthma with onset < 12 years of age in UK biobank (Pividori et al. 2019). In our study, the top SNP at 17q21 had an OR of 1.64 in the overall discovery analysis. In analyses stratified on severity, the OR increased from 1.31 in children with 1 hospitalization to 2.32 in children with >= 6 hospitalizations in iPSYCH, and from an OR of 1.69 in children with 2 hospitalizations to 2.43 in children with >=6 hospitalizations in COPSACsevere.

B. Can you add the replication and meta-analysis results (OR and P) for the five previously known SNPs to table 1?

R2. Thanks for your suggestion. We have now added replication results for known loci to Supplementary Table 4. We have done this for the COPSAC birth cohorts and for UK Biobank replication (asthma below 7 yrs). We have added additional replication of the *FUT2/MAMSTR*

locus from the COAST birth cohort Unfortunately, we do not have results for known loci from this cohort for practical reasons, but these should be available during the coming months and can be added during a potential revision.

We have not added these "replication" results for known loci to the main table in order not to confuse the study flow. These are all robustly replicated well known loci, and we are not trying to replicate these in this study. We only try to replicate the novel locus on chr 19, and as described below, we have now sought additional replication of this locus in the US COAST study with a birth cohort setup resembling the COPSAC birth cohorts, and this has been added to Table 1. Also, we have added individual results from the two discovery studies to Table 1, as requested by reviewer 2 (**R30**), already making this table a little crowded. "Replication results" for the known loci can of course be added to Table 1 at the discretion of the reviewers or Editors.

C. The authors claim the larger effect sizes in the COPSACsevere reflect the more severe phenotype in this cohort. Given the overlap in the confidence intervals of ORs for the two cohorts are the differences between ORs significant?

R3. Thanks for your comment. The ORs are significantly different between the discovery cohorts for the SNPs in or near *GSDMB, HLA-DQA1, IL33* (rs1342326), and *IL13* as seen from the significant p-values of heterogeneity in the discovery stage meta-analysis as reported in Table 1.

Does including the number of hospitalizations (as a proxy for severity) makes the ORs in the two cohorts to converge?

R4. We thank the reviewer for the comment. We have now looked further into this by stratified analyses based on number of hospitalizations in both COPSACsevere and iPSYCH, and indeed this makes the OR between the two studies converge. In the COPSAC severe cohort, with a considerable number of children in each stratum, there was a clear tendency of higher effect estimates (ORs) in the strata with a larger number of hospitalizations, as expected. In the iPSYCH cohort, there were much fewer children with more than 1 hospitalization, but still there was a clear tendency of higher effect sizes with increasing number of hospitalizations, and

consistently for all SNPs the effects size was higher in children with 6 or more hospitalizations compared to children with only 1 hospitalization (see table below).

COPSACsevere stratified on number of hospitalizations.

	**Number of hospitalizations**			
	**2**	**3**	**4-5**	**≥ 6**
	**OR [95% CI], p**	**OR [95% CI], p**	**OR [95% CI], p**	**OR [95% CI], p**
GSDMB	1.69 [1.42 ; 2.02] 5.39e-9	1.93 [1.60 ; 2.34] 1.81e-11	2.08 [1.74 ; 2.48] 6.69e-16	2.43 [2.07 ; 2.87] 1.23e-26
CDHR3	1.37 [1.10 ; 1.69] 0.004	1.48 [1.17 ; 1.85] 7.0e-04	1.45 [1.17 ; 1.78] 5.2e-04	1.65 [1.38 – 1.98], 5.3e-08
HLA-DQA1	1.18 [0.97 ; 1.45] 0.10	1.20 [0.97 ; 1.48], 0.10	1.56 [1.28 ; 1.92] 1.6e-05	1.56 [1.30 ; 1.87], 1.9e-06
IL33 ***(*** rs340933***)***	1.31 [1.01 ; 1.71] 0.04	1.44 [1.09 ; 1.94] 0.01	1.37 [1.06 ; 1.79] 0.02	1.68 [1.32 ; 2.17] 4.7e-05
IL33 ***(*** rs1342326***)***	1.28 [1.02 ; 1.60] 0.03	1.23 [0.96 ; 1.56] 0.09	1.50 [1.21 ; 1.85] 1.62e-04	1.74 [1.44 ; 2.08] 3.11e-11
IL1RL1	1.35 [1.00 ; 1.85], 0.06	1.30 [0.96 ; 1.82], 0.11	1.21 [0.92 ; 1.64], 0.19	1.86 [1.40 ; 2.54], 4.57e-05
WDR36	1.20 [1.01 ; 1.44] 0.04	1.36 [1.13 ; 1.64] 0.001	1.22 [1.02 ; 1.45] 0.02	1.27 [1.09 ; 1.48] 2.1e-03
IL13	1.16 [0.94 ; 1.42] 0.17	1.36 [1.09 ; 1.67] 0.005	1.46 [1.21 ; 1.77] 9.7e-05	1.37 [1.15 ; 1.63] 4.7e-04
FUT2	1.15 [0.97 ; 1.38] 0.11	1.28 [1.06 ; 1.55] 0.01	1.10 [0.93 ; 1.31] 0.27	1.35 [1.16 ; 1.58] 1.4e-04

iPSYCH stratified on number of hospitalizations.

	Number of hospitalizations				
	1	2	3	4-5	≥ 6
	OR [95% CI], p	OR [95% CI], p	OR [95% CI], p	OR [95% CI], p	OR [95% CI], p
GSDMB	1.31 [1.21;1.42], 3.94e-11	1.74 [1.46 ; 2.08], 5.46e-10	2.45 [1.77 ; 3.43], 1.01e-07	2.34 [1.51 ; 3.69], 1.82e-04	2.32 [1.38; 4.04], 0.001
CDHR3	1.34 [1.22 ; 1.48], 3.22e-9	1.22 [0.98 ; 1.52], 0.07	1.72 [1.21 ; 2.42], 0.002	1.89 [1.17 ; 2.96], 0.007	2.17 [1.24 ; 3.78], 0.005
HLA-DQA1	1.12 [1.03 ; 1.21] 0.008	1.39 [1.16 ; 1.67] 3.71e-04	1.46 [1.05 ; 2.03] 0.02	1.68 [1.08 ; 2.65] 0.02	1.39 [0.82 ; 2.38] 0.23
IL33 ***(*** rs340933***)***	1.14 [1.04 ; 1.25], 0.006	1.16 [0.95 ; 1. 41], 0.14	1.10 [0.75 ; 1.56], 0.62	1.46 [0.91 ; 2.28], 0.10	1.36 [0.76 ; 2.32], 0.27
IL33 ***(*** rs1342326***)***	1.18 [1.06 ; 1.31], 0.001	1.40 [1.13 ; 1.72], 0.002	1.24 [0.82 ; 1.81], 0.29	1.50 [0.88 ; 2.44], 0.11	1.46 [0.76 ; 2.60], 0.22
IL1RL1	1.26 [1.10 ; 1.44] 7.4e-04	2.28 [1.60 ; 3.37] 1.16e-05	1.06 [0.67 ; 1.76] 0.83	1.08 [0.58 ; 2.23] 0.81	2.71 [1.00 ; 11.13] 0.09
WDR36	1.14 [1.05 ; 1.23] 0.002	1.20 [1.00 ; 1.42] 0.04	1.20 [0.88 ; 1.64] 0.24	1.40 [0.92 ; 2.12] 0.11	2.07 [1.26;3.43] 0.004
IL13	1.10 [0.99 ; 1.20] 0.07	1.33 [1.09 ; 1.61] 0.004	1.06 [0.72 ; 1.52], 0.76	1.28 [0.78 ; 2.03], 0.31	1.71 [0.98 ; 2.88], 0.05
FUT2	1.16 [1.07 ; 1.26]	1.23 [1.09 ; 1.61]	1.05 [0.77 ; 1.43],	1.15 [0.76 ; 1.77],	1.66 [0.99 ; 2.83],

	2.70e-04	0.004	0.75	0.51	0.06

Indeed, these stratified analyses makes the effect sizes converge between the two discovery studies, and tests of heterogeneity between the two studies are mainly non-significant as shown in the combined table below with tests of heterogeneity between studies for each stratum. In the few comparisons where there is a tendency of heterogeneity (*IL1RL1*), it is now actually in the direction of larger effect size in iPSYCH (but none of these differences are statistically significant considering multiple testing).

Compared COPSACsevere and iPSYCH results in hospitalization strata.

Locus	Study	1		2		3		4-5		6	
		**OR [95% CI], p**	**Het-p**	**OR [95% CI], p**	**Het-p**	**OR [95% CI] p**	**Het-p**	**OR [95% CI] p**	**Het-p**	**OR [95% CI] p**	**Het-p**
**GSDMB**	iPSYCH	1.31 [1.21;1.42], 3,94e-11	-	1.74 [1.46 ; 2.08], 5.46e-10	0.82	2.45 [1.77 ; 3.43], 1.01e-07	0.22	2.34 [1.51 ; 3.69], 1.82e-04	0.63	2.32 [1.38; 4.04], 0.001	0.87
	COPSACsevere	-		1.69 [1.42 ; 2.02] 5.39e-9		1.93 [1.60 ; 2.34] 1.81e-11		2.08 [1.74 ; 2.48] 6.69e-16		2.43 [2.07 ; 2.87] 1.23e-26	
**CDHR3**	iPSYCH	1.34 [1.22 ; 1.48], 3.22e-9	-	1.22 [0.98 ; 1.52], 0.07	0.46	1.72 [1.21 ; 2.42], 0.002	0.48	1.89 [1.17 ; 2.96], 0.007	0.30	2.17 [1.24 ; 3.78], 0.005	0.36
	COPSACsevere	-		1.37 [1.10 ; 1.69] 0.004		1.48 [1.17 ; 1.85] 7.0e-04		1.45 [1.17 ; 1.78] 5.2e-04		1.65 [1.38 – 1.98], 5.3e-08	
**HLA-DQA1**	iPSYCH	1.12 [1.03 ; 1.21] 0.008	-	1.39 [1.16 ; 1.67] 3.71e-04	0.24	1.46 [1.05 ; 2.03] 0.02	0.33	1.68 [1.08 ; 2.65] 0.02	0.77	1.39 [0.82 ; 2.38] 0.23	0.69
	COPSACsevere	-		1.18 [0.97 ; 1.45] 0.10		1.20 [0.97 ; 1.48], 0.10		1.56 [1.28 ; 1.92] 1.6e-05		1.56 [1.30 ; 1.87], 1.9e-06	
**IL33** (rs340933)	iPSYCH	1.14 [1.04 ; 1.25], 0.006	-	1.16 [0.95 ; 1. 41], 0.14	0.39	1.10 [0.75 ; 1.56], 0.62	0.26	1.46 [0.91 ; 2.28], 0.10	0.91	1.36 [0.76 ; 2.32], 0.27	0.50
	COPSACsevere	-		1.31 [1.01 ; 1.71] 0.04		1.44 [1.09 ; 1.94] 0.01		1.37 [1.06 ; 1.79] 0.02		1.68 [1.32 ; 2.17] 4.7e-05	

**IL33**<br>(rs1342326)	iPSYCH	1.18<br>[1.06 ; 1.31],<br>0.001	-	1.40<br>[1.13 ; 1.72],<br>0.002	0.57	1.24<br>[0.82 ; 1.81],<br>0.29	0.97	1.50<br>[0.88 ; 2.44],<br>0.11	1.00	1.46<br>[0.76 ; 2.60],<br>0.22	0.59
	COPSACsevere	-		1.28<br>[1.02 ; 1.60]<br>0.03		1.23<br>[0.96 ; 1.56]<br>0.09		1.50<br>[1.21 ; 1.85]<br>1.62e-04		1.74<br>[1.44 ; 2.08]<br>3.11e-11	
**IL1RL1**	iPSYCH	1.26<br>[1.10 ; 1.44]<br>7.4e-04	-	2.28<br>[1.60 ; 3.37]<br>1.16e-05	0.03	1.06<br>[0.67 ; 1.76]<br>0.83	0.48	1.08<br>[0.58 ; 2.23]<br>0.81	0.76	2.71<br>[1.00 ; 11.13]<br>0.09	0.55
	COPSACsevere	-		1.35<br>[1.00 ; 1.85],<br>0.06		1.30<br>[0.96 ; 1.82],<br>0.11		1.21<br>[0.92 ; 1.64],<br>0.19		1.86<br>[1.40 ; 2.54],<br>4.57e-05	
**WDR36**	iPSYCH	1.14<br>[1.05 ; 1.23]<br>0.002	-	1.20<br>[1.00 ; 1.42]<br>0.04	1	1.20<br>[0.88 ; 1.64]<br>0.24	0.50	1.40<br>[0.92 ; 2.12]<br>0.11	0.55	2.07<br>[1.26;3.43]<br>0.004	0.07
	COPSACsevere	-		1.20<br>[1.01 ; 1.44]<br>0.04		1.36<br>[1.13 ; 1.64]<br>0.001		1.22<br>[1.02 ; 1.45]<br>0.02		1.27<br>[1.09 ; 1.48]<br>2.1e-03	
**IL13**	iPSYCH	1.10<br>[0.99 ; 1.20]<br>0.07	-	1.33<br>[1.09 ; 1.61]<br>0.004	0.34	1.06<br>[0.72 ; 1.52],<br>0.76	0.25	1.28<br>[0.78 ; 2.03],<br>0.31	0.62	1.71<br>[0.98 ; 2.88],<br>0.05	0.44
	COPSACsevere	-		1.16<br>[0.94 ; 1.42]<br>0.17		1.36<br>[1.09 ; 1.67]<br>0.005		1.46<br>[1.21 ; 1.77]<br>9.7e-05		1.37<br>[1.15 ; 1.63]<br>4.7e-04	
**FUT2**	iPSYCH	1.16<br>[1.07 ; 1.26]<br>2.70e-04	-	1.23<br>[1.09 ; 1.61]<br>0.004	0.62	1.05<br>[0.77 ; 1.43],<br>0.75	0.29	1.15<br>[0.76 ; 1.77],<br>0.51	0.85	1.66<br>[0.99 ; 2.83],<br>0.06	0.46
	COPSACsevere	-		1.15<br>[0.97 ; 1.38]<br>0.11		1.28<br>[1.06 ; 1.55]<br>0.01		1.10<br>[0.93 ; 1.31]<br>0.27		1.35<br>[1.16 ; 1.58]<br>1.4e-04	

This table has now been added to the online supplement (Supplementary Table 2) and commented on in the manuscript:

“There was significant heterogeneity between the discovery studies for 4 out of 9 top loci. As expected, this was consistently due to higher effect sizes in the COPSACsevere cohort with a more severe and specific phenotype, in terms of a higher number of hospitalizations (Supplementary tables 1 and 2). Stratified analyses based on number of hospitalizations consistently showed highest effect sizes in the group of children with most frequent

hospitalizations in both COPSACsevere and iPSYCH, and resulted in comparable effect sizes between studies (**Supplementary Table 2**).

Even though restricting analyses in iPSYCH to children with a higher number of hospitalizations would result in more homogeneity between the discovery studies, it would significantly reduce the discovery potential, since the large group with only 1 hospitalization contributes significantly to the statistical power, and we therefore chose to retain all children in the analyses.

D. I checked the recent study of childhood asthma from UK biobank (Ferreira, et al, AJHG, 2019) and the FUT2/MAMSRT signal is not detected in this (larger) cohort can the authors comment on this?

R5. Thanks for your comment. The UK biobank study is based on adults, and the asthma phenotype is very different than our phenotype of early childhood asthma (<7 years) with severe exacerbations. It is therefore not surprising that the results differ from ours, in line with the rationale of performing phenotype specific studies. Ferreira et al. studied asthma with reported onset before 19 years of age (PMID: 30929728) which is quite different compared to the phenotype defined in the current study.

However, we did actually observe a significant association for the *FUT2/MAMSTR* SNP in the UK Biobank dataset when specifically using a case definition of childhood asthma with reported onset before 7 years of age, similar to the current study (**OR = 1.06 (95% CI = 1.02 – 1.10), *P* = 0.002**), while there was no association with asthma with reported onset after age 7 years (**OR = 1.00 (95%CI = 0.98 - 1.02), *P* = 0.96**)). It is remarkable and reassuring that the *FUT2* signal is also seen in another independent population and even in the UK Biobank study based on adults and relying on recall of asthma debut many years earlier in life with respect to the early onset definition. This age-dependent replication emphasizes the importance of phenotype specificity, and this replication result has now been included in the manuscript. Since the phenotype is very different (age at onset based on recall in adults), we did not add this to the meta-analyses, where it would skew data and add substantial heterogeneity, but added it to the results.

E. Given the difference in age distribution for the two discovery cohorts (Table S1), the large difference age between cases and controls, and the difference in asthma risk between age groups (see Ferreira, et al, AJHG, 2019) it seems to me that age is an appropriate covariate to include in the model. Does the inclusion of the age affect the association results (strength and significance)?

R6. We thank the reviewer for this comment. The difference in age distribution between COPSACsevere and iPSYCH is not as big as implied in Table S1. The numbers in COPSACsevere were based on age of fulfilling the criteria for participation in COPSACsevere, which was a minimum of 2 hospitalizations between 2 and 7 years of age. If we calculate the debut based on the first asthma related hospitalization ever, the age distribution is similar between the two studies (COPSACsevere median age = 2.2. iPSYCH median age = 1.8). This is a more meaningful comparison and Supplementary Table 1 has now been updated accordingly.
As the age refers to the age of asthma diagnosis/hospitalization, it is difficult to include this as a covariate in the analysis, since it is not possible to include a proper age for the controls.

F. Figure S1 shows that the lambda is 1.11 for the meta-analysis. Can the authors comment on the inflation of lambda? Could the inflation of test statistics reflect the lack of adequate correction for age or other confounders?

R7. We thank the reviewer for the comment. In the updated GWAS with higher genome-wide coverage, the lambda value is 1.13. This is in-line with other childhood asthma GWAS, i.e. a lambda value of 1.17 was in a study of UK biobank data of childhood asthma (Ferreira, et al, AJHG, 2019; PMID: 30929728). Likewise, larger lambda values were presented in a similar study (Ferreira, et al, Nature Genetics, 2017; PMID: 29083406) of 1.15 and 1.22 for UK Biobank and 23andMe, respectively. Because the lambda value is lower than in other asthma GWASs, we do not believe this reflects a lack of correction in our study.

G. In figure 2 the signal at CDHR3 and IL33 looks unusual to me, the expected LD tail is not present for these two signals that raise concerns regarding false positives. Can the authors comment on this?

R8. As described below, we have now increased in the number of examined SNPs in the revised study (from 32.669 to 525.976 using additional genotyping data), and the updated results show the expected LD tail for these two loci (see main figure 1 and the individual Manhattan plots for the discovery studies supplementary figure 3). The LD pattern for the *CDHR3* variant, reflecting a relatively narrow LD region, is similar to what has previously been reported (Bønnelykke et al. 2014; PMID: 24241537).

H. The authors mention that a random effect model was also applied at the discovery stage what are the fixed and random effects included in this model? If the random effect is the individual is this because there are overlaps between the two discovery cohorts? The p-values for the random effect model are largely different from the meta-analysis and logistic regression p-values why is that? What are the ORs for the random effect?

R9. We thank the reviewer for this comment. The discovery meta-analysis was solely based on the summary statistics data from each of the COPSACsevere and IPSYCH studies. The fixed vs random effects models refer to expected differences in effect size between the studies in the meta-analysis. If there is a large heterogeneity between the effect estimates in the two discovery studies, then the results from the random effect and fixed effect will be different, as the variance between the studies are included in the random effects model, providing different effect estimates and standard errors.

Similar to most other meta GWAS we used a fixed effect model in the primary discovery analysis, since the random effects model is normally considered too conservative (as also illustrated in this study by the weak random-effects p-value for the very strong 17q21 locus).

However, we report both fixed and random effects results as further information to the reader, in order to be able to judge the potential effect of heterogeneity. The ORs for the random effects model (discovery meta-analysis) have now been added to Table 1.

Importantly, there was no heterogeneity between the studies for our novel locus near FUT2, meaning that the choice of fixed vs random effects model did not affect this locus, which is genome-wide significant in both models.

2 - Replication cohort included 185 pediatric cases and 787 controls from COPSAC 2010 cohort plus an additional 411 (healthy?) children from COPSAC 2000 cohort. My concerns regarding the replications analysis are

A. I had a hard time understanding why the authors did the replication analysis using a Cox model instead of logistic regression. Yes, the cohort designs are different and COPSAC 2000 have probably higher asthma risk than average controls but what is the replication the ORs and p-values if you do perform a case-control analysis? What are the ORs and p-values in the COPSAC 2010 only (are the p-values nominally significant? Are the ORs similar to the discovery cohort?)

R10. We are sorry for not being very clear. The 191 cases and 785 controls are the total numbers from the combined COPSAC2000 and COPSAC2010 cohorts. There are cases and controls in each cohort (59 cases and 299 controls in COPSAC2000, and 132 cases and 486 controls in COPSAC2010), and the children have been followed and diagnosed according to very similar diagnostic algorithms and methods. In the combined analysis, any potential difference between studies is adjusted for by including study as covariate in the model.

In the replication of the novel locus, we used a case-control approach (logistic regression model) in order to make the method comparable to the discovery stage and to enable meta-analysis of the results (Table 1).

When studying interaction and doing stratified analysis in the COPSAC birth cohorts (eg Table 3A) we chose using a Cox proportional hazard model. This is the standard way of analyzing asthma and asthma-related outcomes in our birth cohorts, because it takes optimal advantage of the longitudinal design of the cohorts. Cox regression is designed exactly for this purpose, by allowing all individuals to participate (contribute with risk time) as long as they are in the study, and it therefore has better statistical power compared to a logistic regression model, which can only include individuals with full follow-up. The primary difference between the models is thus, that controls without follow-up for the first 6 years of life are removed using the logistic regression model, whereas they are included in the Cox model until they are censored.

However, as the follow-up rate in the COPSAC cohorts is relatively high, there is no major difference between the logistic regression results and the Cox proportional hazards model, as demonstrated below:

Using cox-proportional hazards:

Gene	Analysis	HR [95% CI]	P	cases/controls
FUT2	Main effect	1.27 [1.04; 1.57]	**0.02**	191/785
	ABO = TT	0.99 [0.70;1.41]	0.97	64/338
	ABO = TC	1.35 [1.01;1.80]	**0.04**	103/360
	ABO = CC	2.01 [1.08;3.77]	**0.03**	24/87
ABO	Main effect	1.22 [0.99; 1.50]	0.07	191/785
	FUT2 = AA	0.94 [0.53;1.65]	0.84	27/148
	FUT2 = AG	1.07 [0.79;1.45]	0.66	93/403
	FUT2 = GG	1.65 [1.16;2.35]	**0.005**	71/234
FUT2 × ABO	Interaction	1.38 [1.01; 1.87]	**0.04**	191/785
	Risk score	2.84 [1.55; 5.22]	$\mathbf{7.7 \times 10^{-4}}$	191/785

Using logistic regression:

Gene	Analysis	OR [95% CI]	P	cases/controls
FUT2	Main effect	1.31 [1.04; 1.67]	**0.02**	191/727
	ABO = TT	0.99 [0.67;1.47]	0.96	64/317
	ABO = TC	1.38 [0.99;1.94]	0.06	103/327
	ABO = CC	2.44 [1.20;5.30]	**0.02**	24/83
	Main effect	1.27 [1.00; 1.62]	**0.05**	191/727

ABO	FUT2 = AA	0.94 [0.49;1.73]	0.84	27/142
	FUT2 = AG	1.10 [0.78;1.55]	0.57	93/367
	FUT2 = GG	1.93 [1.26;3.00]	**0.003**	71/218
FUT2 × ABO	Interaction	1.50 [1.05; 2.17]	**0.03**	191/727
	Risk score	2.82 [1.56; 5.10]	**5.7×10^{-4}**	191/727

Using cox-proportional hazards:

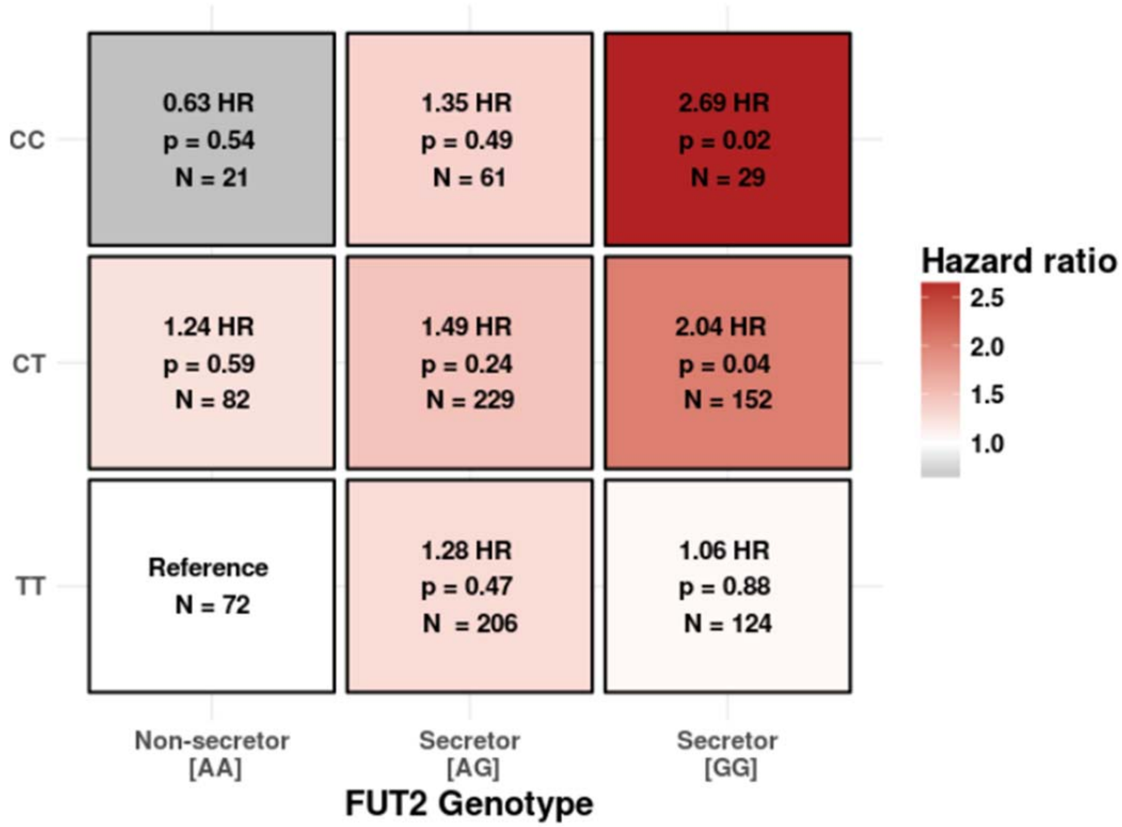

Using logistic regression:

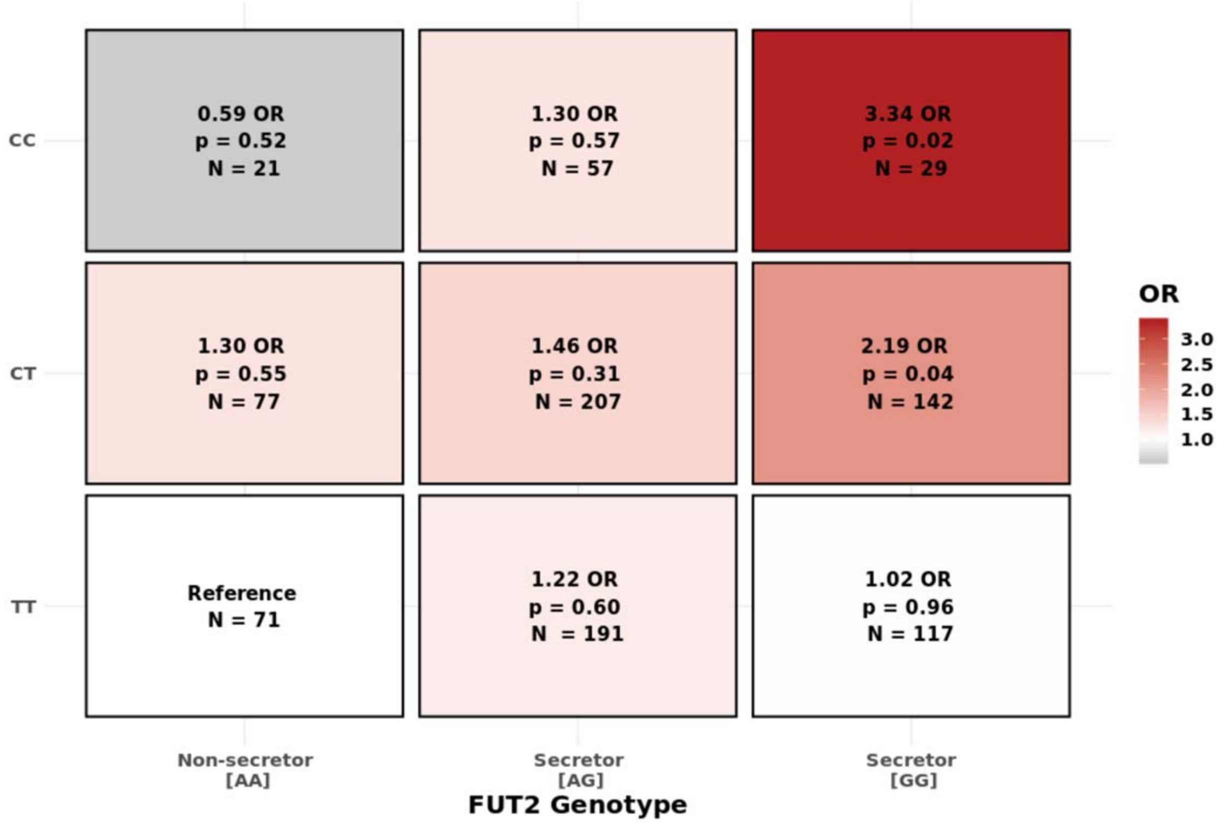

<u>According to the reviewers suggestion and in order to avoid confusion and changes in analytical model in the study, we have now changed all analyses to logistic regression and ORs instead of HRs.</u>

The HR is now only used in Figure 4 to provide a statistical model corresponding to the Kaplan Meier plots illustrated in the figure.

The main difference between the two COPSAC cohorts is that COPSAC2000 is a high-risk asthma cohort, as all the mothers have an asthma diagnosis. This inherent difference is mitigated by adjusting for cohort in all COPSAC birth cohort analyses. The two COPSAC birth cohorts were combined in order to obtain sufficient statistical power. As seen below, the FUT2 estimates were not significantly different between the two cohorts:

The *FUT2* effect in COPSAC2010: OR = 1.31 (95% CI = 0.98 - 1.76), p = 0.07

The *FUT2* effect in COPSAC2000: OR = 1.67 (95% CI = 1.10 - 2.57), p = 0.02

B. What are the frequencies of tag SNPs in COPSAC 2000 and COPSAC 2010?

R11. The MAFs are almost identical in the two cohorts:
MAF for rs281379 in COPSAC2000 is 47.1%, and for COPSAC2010 46.0%
MAF for the functional *FUT2* SNP (rs601338) in COPSAC2000 is 44.0%, and for COPSAC2010 43.1%

C. Are the HR from the Cox model and the ORs from logistic regression/meta-analysis really comparable? I frankly don't know. Can you comment?

R12. Thanks for your comment. Both the HR obtained from a Cox proportional hazard and ORs from a logistic regression provides an estimate of the risk rate and are therefore to some extent comparable (eg on OR of 2 and a HR of 2 will often be interpreted as a doubled risk, although this is not strictly correct statistically). Please see reply R10 for comparison of results using OR and HR. As described above, we now provide the estimates for the birth cohort results using OR instead.

3 - The authors use "SNP clumping" to identify the index SNPs at each locus followed by conditional analysis to identify independent signals within the locus. Why identifying the causal SNPs at a locus with highly linked variants is challenging the standard practice is to perform fine-mapping to narrow down the association signal at the FUT2/ MAMSRT locus as the SNP with the lowest p-value might not be the SNP with the highest likelihood to derive the association signal. For further information on fine-mapping see Schaid et al Nat Rew Gen, 2018

R13. As suggested by the reviewer, we have now applied Bayesian fine-mapping within the *FUT2/MAMSTR* region (Mahajan et al. 2018; PMID: 30297969). The region was defined as all SNPs in +- 500kb from the lead SNP in the region. The fine-mapping was done by calculating the posterior probability that a variant in the region is causal. The variants were subsequently sorted according to their posterior probability, and the 99% credible set of SNPs was defined as

those that have a cumulative probability of 99%. The following SNPs were included in the 99% credible SNP set for the region, indicating that there is a 99% probability that the true causal variant is among the set. The credible sets were calculated using the "corrcoverage" package in R. We then applied Ensemble's variant effect predictor (VEP; PMID: 27268795; https://www.ensembl.org/info/docs/tools/vep/index.html) on the variants included in the credible set for the *MAMSTR/FUT2* region. These results provided no clear single causal variant in the region. The single SNP with highest posterior probability was related to MAMSTR. On the other hand, the results suggested a 59% probability that the true causal variant impacts *FUT2*, a 37% probability that the causal variant affects *MAMSTR*, and a 0.9% probability that the causal variant impacts *RASIP1* (all three genes being part of a gene cluster ranging 44.6 Kb on Chromosome 19). Furthermore, the rs601338 FUT2 functional SNP has the highest impact on the affected gene according to VEP and on the SNP pathogenicity prediction through Combined Annotation Dependent Depletion (CADD) scores (PMID: 24487276; Supplementary Table 7 and Supplementary Table 8)

These results have been added as Supplementary Table 7:

**SNP**	**Posterior probability**	**Consequence**	**Affected gene (Chr. 19 locus)**	**Impact**
rs281379	0.37	Downstream gene variant	*MAMSTR*	Modifier
rs503279	0.20	3' UTR variant	*FUT2*	Modifier
rs504963	0.15	3' UTR variant	*FUT2*	Modifier
rs485186	0.12	Synonymous variant	*FUT2*	Low
rs602662	0.09	Missense variant	*FUT2*	Moderate
rs601338	0.02	Stop gained	*FUT2*	High
rs2287921	0.01	Intron variant	*RASIP1*	Modifier
rs516246	0.01	Intron variant	*FUT2*	Modifier
rs2287922	0.009	Missense variant	*RASIP1*	Moderate

And as Supplementary Table 8

SNP	Position (Base pairs)	Raw Score	PHRED (CADD)	SIFT prediction	PolyPhen2 prediction	Annotation	Gene
rs601338	49206674	9.18	48	damaging	Probably damaging	Stop Gain	*FUT2*
rs2287922	49232226	3.75	26.2	damaging	Probably damaging	Missense	*RASIP1*
rs602662	49206985	2.47	22.5	tolerated	Probably damaging	Missense	*FUT2*
rs2287921	49228272	0.298	7.19	-	-	Intron	*RASIP1*
rs281379	49214274	0.129	4.75	-	-	-	*MAMSTR/FUT2*
rs503279	49209010	-0.058	1.83	-	-	3'UTR	*FUT2*
rs504963	49208865	-0.018	2.35	-	-	3'UTR	*FUT2*
rs485186	49207206	-0.258	0.48	-	-	Upstream	*FUT2*
rs516246	49206172	-0.25	0.51	-	-	Intron	*FUT2*

SNP: Single Nucleotide Polymorphism; BP: SNP position in Base Pairs corresponding to dbSNP Assembly GRCh37.p13; SIFT: Sorting Intolerant from Tolerant (https://sift.bii.a-star.edu.sg); PolyPhen2 (**Poly**morphism **Phen**otyping v**2; http://genetics.bwh.harvard.edu/pph2/dokuwiki/about**).

and described in the main text:

"Applying gene expression data from nasal epithelium cells (NEC) obtained from the COPSAC2010 cohort, we examined the presence of eQTL signals for the FUT2/MAMSTR top SNP, rs281379. This SNP was a strong eQTL for FUT2 (Beta = 0.71, (95% CI = 0.66 – 0.77), P = 2.05 × 10-78), where the A allele reduced the expression of FUT2. This SNP was also a strong eQTL for RASIP (Beta = -0.42, (95% CI = -0.52 - -0.32), P = 3.47 × 10-14) and a weak eQTL for FAM83E (Beta = -0.06, (95% CI = -0.12 - -0.01), P = 0.02), and RPL18 (Beta = 0.03, (95% CI = 0.00 - 0.06), P = 0.02) (Supplementary Table 7).

In an attempt to determine the causal variant for the FUT2/MAMSTR locus we applied the Ensemble's variant effect predictor (VEP) using the SNPs in the 99% credible set (Supplementary table 8). Of the 6 SNPs in the credible set, one impacted the MAMSTR gene, and the remaining 5 SNPs had an impact on FUT2, suggesting a 59% probability that the true causal variant impacts FUT2. The credible set included the functional SNP rs601338 (nonsense

mutation, W143X) which was in high linkage disequilibrium (LD, r2 > 0.80, D' = 0.97) with the top SNP rs281379 in the FUT2 locus and showed similar evidence of association with asthma (OR = 1.16 (95% CI = 1.09-1.23), P = 1.6 × 10-7). We further used the Combined Annotation-Dependent Depletion (CADD) database (also PolyPhen2 and SIFT) to identify the functionality (deleterious, disease causal, pathogenicity) of variants at this locus, showing the highest CADD score (48) for the functional *FUT2* SNP, rs601338, followed by rs2287922 related to *RASIP1* with a score of 26 (**Supplementary Table 8**)."

4 - While a causal SNP is not convincingly identified, possible causal genes are given even less attention, thus limiting the impact of the manuscript in understanding childhood asthma pathogenesis.

A. The authors assume that the signal at rs281379 is mapped to the FUT2 gene. The nearest genes to rs281379 are MAMSRT (~2 Kb) and FUT2 (~ 5Kb). However, as shown in figure S2, several other genes exist in the region. What is the justification for choosing FUT2 for further follow up (interaction analysis, risk of infection)?

R14. We agree with the reviewer, that we cannot be sure that the causal variant in the chr 19 region is related to *FUT2*. As described above it can also be, completely or partly, related to another gene in the region. As described above, fine mapping provides no definitive evidence of the functional variant as now also described in the manuscript. However, as also described above, the *FUT2* functional variant is the most deleterious SNP in LD with the top variant rs281379 (LD, $r^2 = 0.81$), the SNP with strongest eQTL support, the one strongest expressed in the airways, and is also supported by the evidence of interaction with *ABO*, which is well known at the biological level. This makes it a likely candidate to explain, at least part of, the association signal at the locus.

B. Is it possible that rs281379 or other SNPs in linkage with it have a regulatory effect on FUT2 or other genes in the region (expression, splicing, methylation, etc)? Is there any evidence of physical interaction with nearby genes?

R15. Using gene expression data obtained from nasal epithelial cells (NEC) in the COPSAC2010, we found that the top-asthma associated SNP rs281379 shows strongest association with expression of *FUT2*, but also with expression of *RASIP* and to a smaller extent *RPL18* and *SPHK2* (see table below). This suggests that this asthma-signal for this SNP could be mediated by *FUT2* expression but also other genes, including *RASIP*, or a combination.

Gene	Estimate (95% CI)	P-value
FUT2	0.71 (0.66 ; 0.77)	2.05e-78
RASIP1	-0.42 (-0.52 ; -0.32)	3.47e-14
FAM83E	-0.06 (-0.12 ; -0.01)	0.02
RPL18	0.03 (0.00 ; 0.06)	0.02
SPHK2	0.03 (-0.04 ;0.11)	0.42
MAMSTR	-0.05 (-0.17 ; 0.07)	0.45
CA11	0.02 (-0.10 ; 0.15)	0.73
FUT1	0.01 (-0.07 ; 0.09)	0.79
DBP	0.01 (-0.06 ; 0.07)	0.82

This table has now been added to the online supplement (Supplementary table 7) and is described in the manuscript:

"Applying gene expression data from nasal epithelium cells (NEC) obtained from the COPSAC2010 cohort, we examined the presence of eQTL signals for the FUT2/MAMSTR top SNP, rs281379. This SNP was a strong eQTL for FUT2 (Beta = 0.71, (95% CI = 0.66 – 0.77), P = 2.05e-78), where the A allele reduced the expression of FUT2. This SNP was also a strong eQTL for RASIP (Beta = -0.42, (95% CI =-0.52 - -0.32), P = 3.47e-14) and a weak eQTL for FAM83E (Beta = -0.06, (95% CI =-0.12 - -0.01, P = 0.02), and RPL18 (Beta = 0.03, (95% CI = 0.00 – 0.06.32), P = 0.02) (**Supplementary Table 6**)."

Similar results were found in the Genotype-Tissue Expression (GTEx) database (https://gtexportal.org/home/). The strongest regulatory evidence of rs281379 in the GTEx database is that it influences the expression of *FUT2* (p = 2.8e-84), where the A allele reduces

the expression in the Esophagus - mucosa. Furthermore, the SNP is also an eQTL for the *RASIP1* gene (p = 1.2e-32) in fibroblasts.

We have found no evidence of physical interaction e.g. due to chromosomal looping.

C. After narrowing the causal SNP and causal gene using in silico/statistical methods, bringing functional validation of the results can bring strong orthogonal support for the author's observations.

5 - After the association analysis, the authors move on to investigate the interaction between the index SNP at FUT2/MAMSRT locus and ABO locus based on the known biological relationship between these two loci. I find the results of analysis speculative and unconvincing due to the following reasons:

A. The interaction is done between a nonsense SNP overlapping FUT2 and the top association signal overlapping ABO which is an intronic SNP.

R16. It is correct that the top signal from the *ABO* locus is the intronic SNP rs505922. However, this SNP is in high LD with the frameshift/deletion polymorphism (rs8176719, r^2=0.96, D'=1.0), which directly encodes the 0- vs A/B blood type. This is the closest tag for 0 blood type in our data, and this SNP is used as tag for 0 blood type in several studies.

This means that we practically test the known biological interaction between secretor (*FUT2*) genotype and 0 vs AB blood type, which is known and characterized extensively in other biological settings – and is highly biologically plausible. In other words, in order to test the biological known interaction between *FUT2* and *ABO*, we would choose rs505922 as the optimal tagging SNP – and this at the same time was the SNP in the *ABO* region with strongest association signal.

The FUT2 nonsense SNP is in LD with the index SNP at FUT2/MAMSRT locus (r2 = 0.8) whoever given the large difference between the MAF of these two alleles (0.23 vs. 0.32) the LD is probably not strong enough to assume that FUT2/MAMSRT index SNP is tagging

this SNP. To me, the biological relevance is not enough to justify this choice of FUT2 nonsense SNP as causal SNP.

R17. The MAF for the top variant rs281379 is 46.63% (gnomAD v2.1.1, https://gnomad.broadinstitute.org/; European (non-Finnish) population), which is similar to the functional *FUT2* variant (rs601338: MAF=0.472 in the gnomAD database), which has a MAF of 44.51% (current study).

As described above, there is no robust evidence that the functional *FUT2* SNP is the causal variant at this locus. However, seeing this GWS association signal near *FUT2* and also an *ABO* signal (basically representing 0 blood type) led to the hypothesis that these two signals could represent the same mechanism – and if this was the case, there should be evidence of interaction (e.g. the *AB0* signal should only be present in secretors defined by *FUT2* status).

The obvious way to test this is to take the functional SNP at FUT2 as well as the functional SNP at *ABO* (tagged by the top SNP at this locus), based on the known biological interaction between *ABO* and *FUT2*, which is specifically mediated by these two functional genetic variations. This effectively allows investigation of this biological plausible interaction on a population scale.

However, we also tested interaction between the top-SNP at the *FUT2/MAMSTR* locus and *ABO* (Table 2), also because regulatory mechanisms related to *FUT2* expression might contribute to the interaction with *ABO*.

With respect to the *FUT2* interaction with *ABO*, we find these two chr 19 SNPs (the top SNP and the functional *FUT2* SNP) to be the only relevant ones to test, and we only tested these two interactions, thereby limiting the number of interaction tests to 2.

B. Moreover, neither the nonsense FUT2 SNPs nor the ABO intronic SNP reaches the genome-wide significance. This combined with the fact that the interaction p-values are not very significant (only interactions in the 6+ hospitalization group pass multiple testing threshold, table S6) makes me wonder if the observed effect can be driven by chance (or by

a biological mechanism unrelated to the secretion of ABO antigens). If you take all variants that are similarly in LD (r2 >= 0.8) with the index SNP at the FUT2/ MAMSRT locus and all the variants in LD with the index SNP at ABO locus how often do one see p-values as strong as the ones in obtained by the interaction analysis? How often does one see a similar or larger OR? And how often do one see the effect getting stronger in the more severe group (6+ hospitalization)? If most of the interactions that are less biological plausibility have a more significant p-value/OR, or a stronger OR in the severe subset of patients what does that mean for the proposed biological mechanism (e.g effect of a FUT2 nonsense variant on ABO the secretor/non-secretor status).

R18. As described above, the interaction test was hypothesis driven limiting this to the best tags for the known biological (functional) variants at *FUT2* and *ABO*. In addition, we tested interaction between the top SNP at *FUT2/MAMSTR* and the *ABO* locus. We therefore only tested 2 interactions, and the p-values for both SNPs are robust for multiple testing (p<6.6*10-4).

Importantly, the interaction is further replicated in the independent COPSAC birth. Not only is the interaction significantly replicated, it also shows the exact same interaction pattern (Table 3.B). Actually, a one-sided p-value test would be appropriate for replicating the interaction, since we will only accept replication in the correct direction. This demonstrates an even more significant replication of the interaction (p = 0.015), which is unlikely to be a chance finding.

Even though we did not perform multiple tests with respect to the interactions, the significance level for the interaction would also survive multiple testing for a large number of SNPs as asked by the reviewer.
There is 1 SNP (rs514659) with $r^2 > 0.80$ for the top *ABO* variant (rs505922) in the revised data set (Updated SNP array for the Inter99 study controls). Likewise, there are 5 SNPs (rs516246, rs681343, rs485186, rs569970, rs503279) with $r^2 > 0.80$ for the top *FUT2* variant (rs281379), resulting in 12 different *ABO-FUT2* combinations (2 *ABO* SNPs and 6 *FUT2* SNPs). Given that the association p-value in COPSACsevere was $p = 3.2 \times 10^{-4}$, this would survive testing of all 12 combinations, and the bonferroni correction for multiple testing is even too conservative given that all of these SNPs are correlated. Similarly, the strong signal observed in the most

pronounced cases ($p = 2.6 \times 10^{-4}$) would hold for multiple testing, when analyzing all possible ABO-FUT2 interactions in each disease severity strata.

Based on 1000 phenotype permutations using the entire COPSACsevere, one permutation resulted in an interaction OR of 1.24, with a p-value of 0.001, indicating a 1 out of 1000 likelihood of a chance finding. Additionally, applying a similar approach within each disease severity strata, 1 out of 10.000 permutations showed an interaction effect with similar statistical significance in both the overall analysis and most severe strata.

C. Can you repeat the interaction using case-only analysis comparing (sever to non-sever cases), what is the OR and p-vale in this analysis? Having the same magnitude and direction of effect in this independent comparison can bring supporting evidence for claims regarding the role of this FUT2xABO interaction in asthma severity.

R19. We thank the reviewer for this suggestion and agree that a case only analysis is important as supportive evidence. Below is a case-only analysis within each disease strata, testing interaction (correlation between the SNPs) between the *ABO* and *FUT2* SNP. This analysis confirms the interaction in children with several hospitalizations similar to what was seen in the case-control model.

Disease strata	β[95% CI]	p-value
**2**	0.01 [-0.13 ; 0.11]	0.80
**3**	0.07 [-0.04 ; 0.19]	0.24
**4-5**	0.07 [-0.04 ; 0.18]	0.21
**6 or more**	0.17 [0.07 ; 0.27]	9.2e-4

D. The conclusion from the interaction analysis states "since it (the interaction) is biologically plausible, it supports the validity of the two individual association signals, including the ABO locus which did not reach genome-wide significance." This is a

dangerous claim to make! Biologically plausible associations can be driven by chance and without a biological basis as proven by many years of candidate gene studies.

R20. We agree that without experimental evidence, this cannot be proven and have removed this sentence.

Minor comments:

1. In the replication cohort, the numbers reported for in table S1 are 185 cases and 727 controls. The number of controls doesn't match the number of controls presented in the methods (787+411 != 727) where does the discrepancy come from?

R21. The number of cases and controls in table S1 refers to the combined number of asthmatics and non-asthmatics in both birth cohorts. Both cohorts include cases and control. The numbers in the methods section were incorrect and this typo has been corrected. We thank the reviewer for noticing this.

2. What is the threshold for removing related individuals?

R22. As per the reviewers comment the following details have now been incorporated in the revised manuscript:

COPSACsevere cases and Inter99 controls: Pairwise Identity by Decent (IBD) estimates were calculated (using the PLINK tool –genome option) in the genetically homogenous (European) individuals and monozygotic twins or genetic duplicates were excluded. Final analyses were adjusted for first 10 principal components (correcting for population sub structure or other relatedness).

iPsych study: Individuals with a homogenous genetic background (after global and local ethnicity filtering using PCA and Mahalanobis distance criteria) were checked for various degrees of kinship using the KING software package (http://people.virginia.edu/~wc9c/KING/).

In total KING identified 1st degree (or closer), 2nd degree (or closer), and 3rd degree (or closer) relative pairs. All "second degree" relatives were excluded ensuring no two subjects were closer than 3rd degree relatives.

3. How the IBD calculation and genotyping QC is done? I assume using plink however no reference was provided.

R23. Yes, we applied PLINK to do the genotype QC, including IBD calculations. This is now referenced in the method section.

4. Can you add the median age of controls to table S1? Can you report the information for the two replication cohorts separately in the table?

R24. For iPSYCH and COPSACsevere both cased and controls were followed prospectively in registries into late childhood/early adulthood.
For the birth cohorts, the majority of children were followed to age 7 years as were the cases.
As noted in response to reviewer 2, we have performed an additional analysis in iPSYCH limiting controls to those not diagnosed with asthma by age 7 years, meaning that this is the last observation point (age) for all controls in that analysis. This analysis showed similar results.

5. Methods are sparsely reported, examples: heterogeneity P.value calculation, random effect model for discovery stage, covariates included in the interaction model.

R25. The methods have been improved with information on details pointed out by the reviewer. We have also added a flow chart depicting the same (Supplementary Figure 7).

6. Can you add a supplementary table for all the variants the passes the genome-wide significance threshold at each of the six loci?

R26. This has now been added as a supplementary table 14, which is a separate excel sheet containing all the variants.

7. Are the risk rations presented in figure 3 statistically different?

R27. The difference between risk ratios is formally tested in the interaction tests presented in the results section. The interaction was borderline significant in the overall discovery analysis, (OR = 1.08 (95% CI = 0.99-1.17), P = 0.06), and significant in the COPSACsevere study with a more severe and specific phenotype definition (interaction OR = 1.27 (95% CI = 1.11-1.45), P = 6.6 × 10-4), in the COPSACsevere study for children with 6 or more hospitalizations (interaction OR = 1.51 (95% CI = 1.21-1.88), P = 2.6 × 10-4), and in the COPSAC birth cohorts (interaction OR = 1.50 (95% CI = 1.05-2.17), P = 0.03) These p-values can be added to the figure if preferred by the reviewers.

Reviewer #2

The two discovery cohorts. Phenotype definition from the two discovery cohorts (COPSAC severe and I Psych) was done based on registry data. However, the phenotype defined was different: 2 or more exacerbations in COPSAC severe, and One or more exacerbations in I Psych. Inconsistencies between the cohorts are interpreted due two these different definitions, but is unclear why the phenotype definition was different?

R28. The phenotype in the $COPSAC_{severe}$ study (more than 2 hospitalizations due to asthma from 2-6 years of age) is rare, and in order to obtain sufficient number of cases in COPSACsevere, we screened app 1.5 million children. Therefore, using similar case criteria in the iPSYCH study of app. 65,000 individuals would results in too few cases for meaningful analyses. We hypothesized that using a related but less strict/severe case definition in iPSYCH would result in similar biological signals with smaller effect sizes but still meaningful power due to large numbers. Indeed, this was also what we found, as also illustrated by the individual Manhattan plots for iPSYCH and COPSACsevere (shown below) showing very similar association patterns.

COPSACsevere

IPSYCH

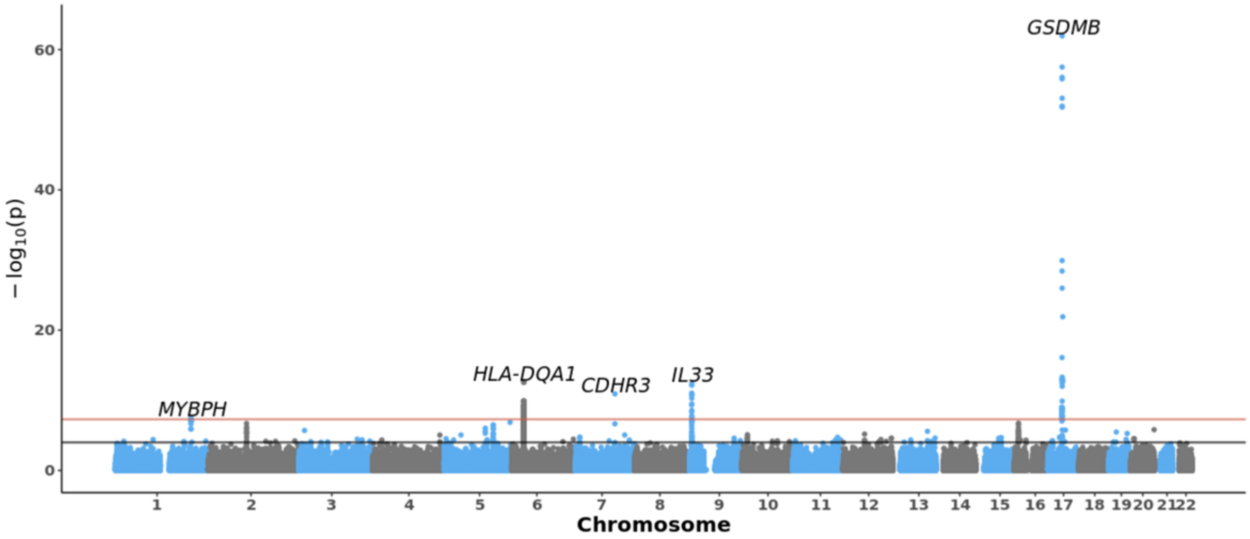

Both these Manhattan plots have now been added to the online supplement and is referred to in the section of the main manuscript addressing the phenotype differences:

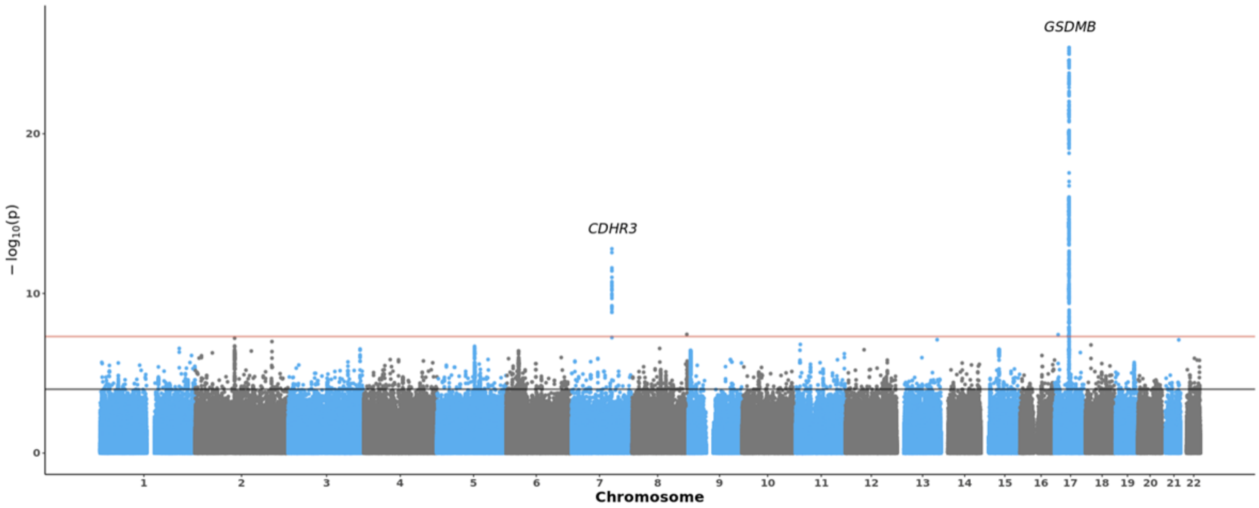

"The association patterns between the two discovery cohorts were similar as illustrated by the individual Manhattan plots **(Supplementary Figure 3)**."

a. Please present the I Psych data stratified by the number of exacerbations (these data are available, according to the supplement).

R29. The table below summarises the number of cases in iPSYCH stratified according to the number of hospitalizations.

**Number of hospitalizations**	**1**	**2**	**3**	**4-5**	**≥ 6**
**Number of cases**	1236	267	83	45	31

For comparison, the table below summarises the number of cases in different disease strata in the COPSAC severe data.

**Number of hospitalizations**	**1**	**2**	**3**	**4-5**	**≥ 6**
**Number of cases**	0	276	240	293	379

b. In table 1, please present cohort specific results, as well the meta analysis, using both fixed and random effects.

R30. These have been added to Table 1 as suggested.

c. For the control selection in I Psych: were subjects with asthma or exacerbations > 6 years included as controls?

R31. In order to achieve the maximum signal-to-noise ratio between the cases and controls, we only retained those controls without any hospitalizations due to asthma exacerbations. We have now performed an additional analysis restricting controls to those not having asthma before age 7 years (including individuals with asthma after age 7 years as controls), which did not change the results:

Locus	Not individuals with asthma after age 7 years as controls		including individuals with asthma after age 7 years as controls	
	OR (95% CI)	P-value	OR (95% CI)	P-value
GSDMB	1.44 (1.35-1.55)	3.5×10^{-25}	1.44 (1.34-1.55)	1.2×10^{-24}
CDHR3	1.36 (1.25-1.48)	2.7×10^{-13}	1.36 (1.26-1.48)	3.3×10^{-13}
HLA-DQA1	1.19 (1.11-1.28)	2.5×10^{-6}	1.18 (1.10-1.27)	4.1×10^{-6}
IL33 (rs340933)	1.30 (1.17-1.44)	1.2×10^{-6}	1.15 (1.06-1.25)	6.2×10^{-4}
IL33 (rs1342326)	1.24 (1.13-1.35)	3.9×10^{-6}	1.23 (1.12-1.35)	5.6×10^{-6}
IL1RL1	1.36 (1.20-1.53)	5.7×10^{-7}	1.35 (1.20-1.53)	6.8×10^{-7}
WDR36	1.17 (1.09-1.26)	1.3×10^{-5}	1.17 (1.09-1.25)	1.7×10^{-5}
IL13	1.14 (1.05-1.24)	1.3×10^{-3}	1.14 (1.05-1.24)	2.0×10^{-3}
FUT2	1.18 (1.10-1.26)	3.2×10^{-6}	1.17(1.09-1.26)	6.9×10^{-6}

2. SNP selection for the exome wide study of COPSACsevere. The number of SNPs in the discovery cohort is drastically reduced due to the study design (Study 1: 263 K SNPs, Study 2: 951 K SNPs, overlap only 32 K SNPs.

a. Could a flow chart be provided how that is possible?

R32. A flow chart has now been added showing the reasons for SNP exclusion.

The previous reduction in SNPs was mainly due to the focus on the exome chip, which has an excess of very low frequency variants. The exome-chip used for the Inter99 cohort includes 247.248 SNPs, of which 234.013 are also available for COPSACsevere. The final number of SNPs are, however, greatly reduced due to the abundance of low-frequency variants on the exome chip. The plot below illustrates the MAF spectrum for the SNPs on the exome chip

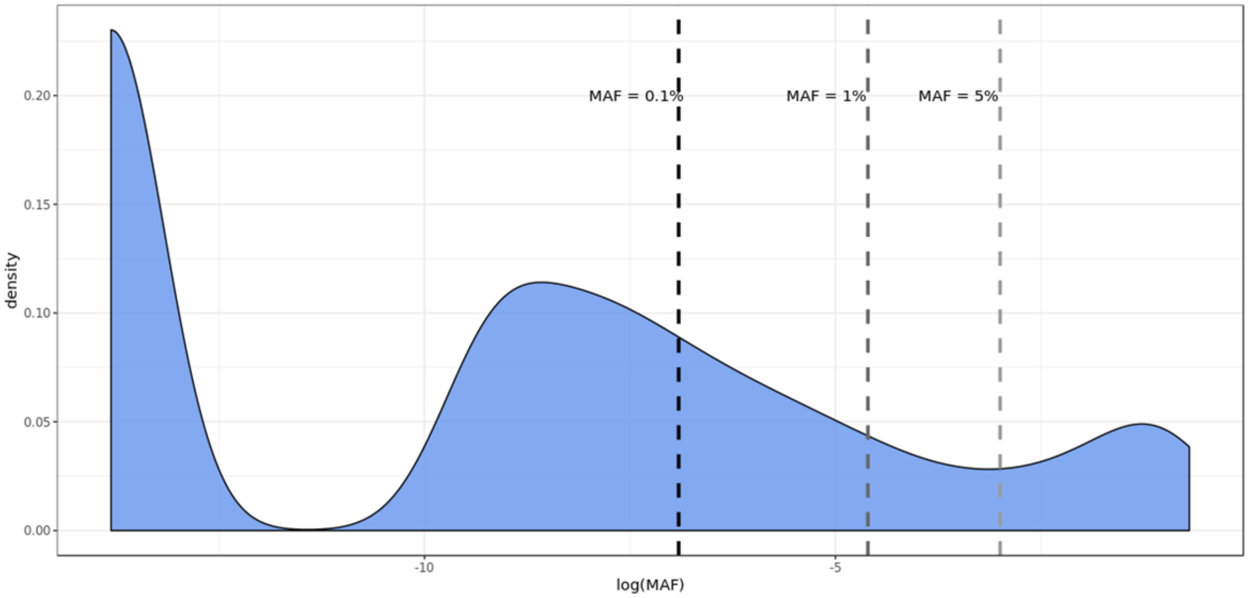

The large peak to the left represents monomorphic SNPs, with 91.551 SNPs being monomorphic. Of the total number of variants on the chip, 40.246 have a MAF > 1%, which is the MAF threshold used in this study due to lack of statistical power to detect rare variants.

In order to increase the genomic resolution of the data, we have now added additional genotyping data from the inter99 cohort.

Furthermore, in order to have a better resolution of the human leukocyte antigen (HLA) genes located on chromosome 6, we performed an imputation of HLA SNPs and HLA amino acid variants using the software the SNP2HLA. We applied the HapMap-CEPH reference panel to impute HLA variants. The reference panel consists of 5.986 biallelic markers, of which 321 are amino acid variants and 109 are classical HLA-alleles at a 4-digit resolution. We used the resulting PLINK files which consists of best-guess genotypes based on posterior probabilities for

downstream analysis. The HLA-imputation was performed for both the COPSACsevere data and iPSYC. This resulted in 6700 additional variants for the HLA-region in both datasets.

The final overlap between iPSYCH and COPSACsevere for the meta-analysis was 414.730 SNPs. This was a significant increase from app. 32.000 SNPs in our first analysis and resulted in additional genome-wide significant loci.

The flowchart illustrates the QC pipeline for the updated genotype data.

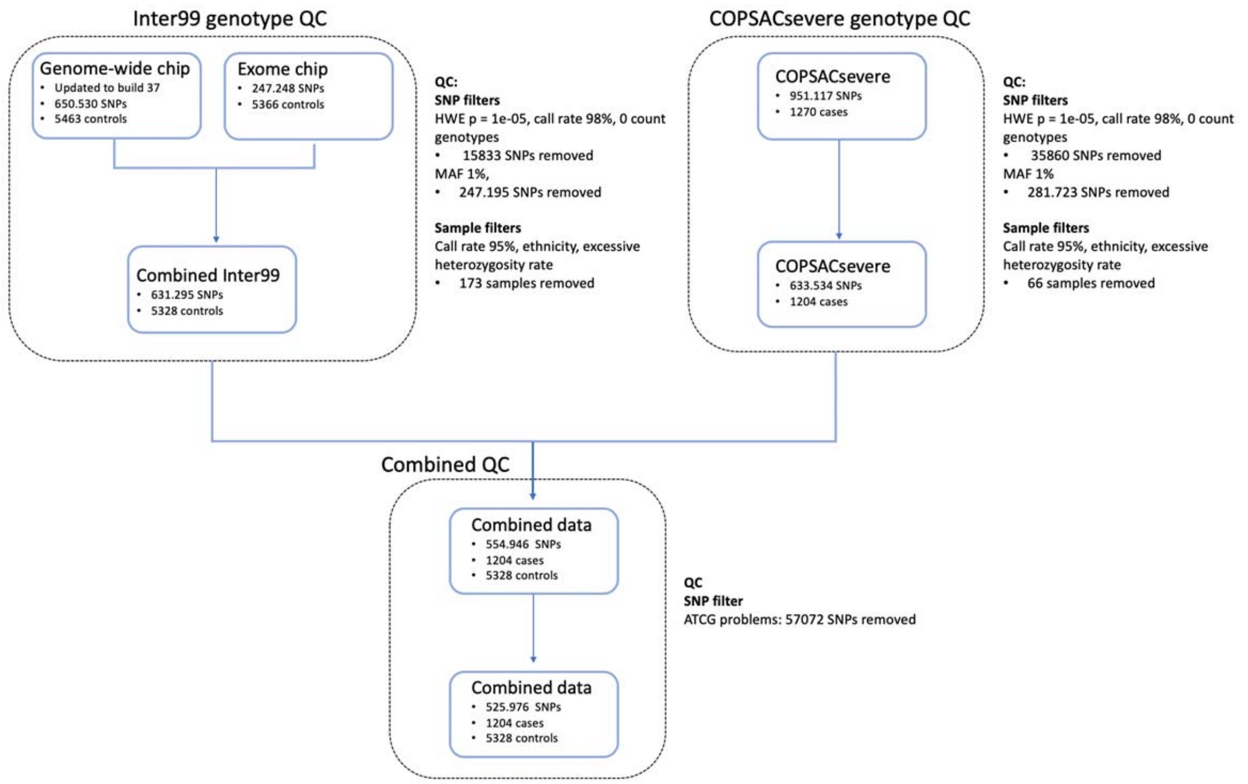

b. Could the authors comment how well this remaining SNPs set covers the exome?

R33. The Illumina exome arrays were designed to focus on the more frequent coding variants seen several times in existing sequence datasets including based on app. 12,000 sequenced genomes and exomes. The arrays include 260.054 non-coding variants fulfilled these criteria, and where included on the chip, which is estimated to include 97-98% on the nonsynonymous

variants detected through sequencing. Similarly, 12662 splice-variants were included on the SNP, which includes 94-95% of the splice altering variants found in exome sequencing. Furthermore, 7137 stop altering variants passed the criteria and were comprised on the chip, which is 94-95% of the stop altering variants are normally detected on the average genome through exome sequencing.

Applying exome sequencing would yield more functional variants, however, many of these would be extremely rare, and unexplorable based on the sample size in the current study. Even though the chip includes be the most "common" functional variants, the vast majority of these are still rare (91.556 monomorphic SNPs in our data), and we apply a MAF filter of 1% to focus on the functional variant which we have the statistical power to examine.

3. Reference of this locus as an (childhood) asthma locus. While this reviewer has no doubt that the findings done in the discovery phase are done for a highly relevant phenotype (hospitalizations labeled as asthma exacerbations), describing a group of children that have severe episodes of dyspnea and bronchoconstriction that necessitates a hospitalization, the further work on this locus needs careful description of the phenotype. Replication was shown for common cold symptoms as well

a. Please provide a detailed description of the asthma definition in COPSAC. Which symptoms were used for this definition? " certain burden of symptoms" is unclear.

R34. Asthma in the COPSAC birth cohorts was diagnosed by doctors in the research clinic based on a previously detailed quantitative symptom algorithm (as previously described XX) requiring all of the following criteria: (1) verified diary recordings of 5 episodes of troublesome lung symptoms (cough, breathlessness or wheeze) within the preceding 6 months, each lasting at least 3 consecutive days; (2) symptomatology typical of asthma including exercise induced symptoms, prolonged nocturnal cough, and persistent cough outside common cold; (3) the rescue use of inhaled β2-agonist; and (4) response to a 3-month course of inhaled corticosteroids followed by relapse after the end of treatment. Remission was defined as a period of 12 months without relapse.

The more detailed description of the asthma definition has now been added to the methods description:

"In both studies, asthma was diagnosed by doctors in the research clinic based on a previously detailed quantitative symptom algorithm[54,55] requiring all of the following criteria: (1) verified diary recordings of 5 episodes of troublesome lung symptoms (cough, breathlessness or wheeze) within the preceding 6 months, each lasting at least 3 consecutive days; (2) symptomatology typical of asthma including exercise induced symptoms, prolonged nocturnal cough, and persistent cough outside common cold; (3) the rescue use of inhaled β2-agonist; and (4) response to a 3-month course of inhaled corticosteroids followed by relapse after the end of treatment. Remission was defined as a period of 12 months without relapse."

b. Perhaps, describing the 2 – 6 year definition as asthma symptoms is a better way forward (as was previously done in COPSAC as troublesome respiratory symptoms).

R35. We agree that the definition of asthma/asthmatic symptoms/wheeze in this age group is difficult with no defined gold standard of diagnosis and large differences in diagnostic tradition between doctors and countries. With respect to the registry diagnosis, we rely on the doctors giving the diagnosis considering this to be asthma (rather than other diagnoses available in the system, such as bronchitis). We also find some "genetic support" for this by the strong signals seen for known asthma genes. In the COPSAC birth cohorts, the recommendation from clinical journals, such as the New England Journal of Medicine, was to call it "persistent wheeze" in the first 3 years of life but ok to call it asthma when following the children to age 5 or 6 years, particularly given the relatively stringent diagnostic criteria. We have now expanded on this in the limitation section of the manuscript:

"The diagnosis of asthma is difficult in early life where there is no diagnostic gold standard and many children outgrow their symptoms, and some physicians prefer the term "wheezing" for asthmatic symptoms before age 6 years. However, the specificity of the diagnosis is probably increased in the present study due to the severity of symptoms for the severe asthma cases and the stringent diagnostic criteria applied in the birth cohorts."

c. In the RhinGen study, was this locus associated with asthma using their definitions? This may help understand the reader a bit more what the phenotypic correlates of this locus are.

R36. No, this locus was not associated with asthma status in the school aged children in Rhinogen (p= 0.74 for the top SNP rs281379). This suggests that this locus is mainly associated with preschool asthma (in line with the results from UK Biobank – see **R5**). But apparently it still confers increased susceptibility to s pneumoniae infections in school age.

MINOR CONCERNS

4. Ascertainment of the cohorts. Are I Psych and Inter99 truly randomly ascertained population based cohorts? Or is any case selection present? This was well presented for I Psych, but is unclear for Inter99

R37. Inter99 is a population-based study recruiting randomly ascertained participants aged 30 to 60 years as part of randomized, non-pharmacological intervention study for the prevention of ischaemic heart disease, conducted on at the Research Centre for Prevention and Health in Glostrup, Denmark (ClinicalTrials.gov: NCT00289237). LXX.

5. R155. A functional SNP is proposed for the FUT2 locus.

a. Is this the only functional SNP or eQTL for the FUT2 top SNP? In the discussion, it is proposed that the FUT2 signal could be due to nearby genes, but why?

R38. The rs601338 is the only functional variant at this locus in high LD (R2>0.7) with the top SNP from the locus. Furthermore, as discussed in reply R15, the top SNP in the region is a strong eQTL for *FUT2*, but also for other genes in the region. We therefore cannot exclude that, at least part of, the association signal could be due to other genes in the region. There are multiple other examples of association signals seemingly involving several nearby genes, including the strongest asthma locus at chromosome 17q (PMID: 29307657). However, given the known biological role of the functional *FUT2* SNP, we believe this is a strong candidate, and particularly we believe that our data strongly suggest this based on the interaction seen between *FUT2* and *ABO* which is very well documented at the biological/biochemical level.

b. Please present exact r2.

R39. The exact r2 between rs601338 and rs281379 is 0.81 in the European population (CEU), this has now been added to the manuscript.

6. The interaction of the FUT2 locus and the ABO was not significant in the discovery dataset. Please provide full data on population specific interactions for the 2 cohorts in the supplement , not only for the COPSACsevere but also for the I Psych, stratified on the number of exacerbations.

R40. It is correct that the interaction was only borderline significant in the meta-analysis in the discovery stage (OR = 1.08 (95% CI = 0.99-1.17), P = 0.06), and this was driven by the COPSACsevere study, and specifically those children with many hospitalizations (>=6) where the interaction was strong (interaction OR = 1.51 (95% CI = 1.21-1.88), $P = 2.6 \times 10^{-4}$).
There were very few children with several hospitalizations in iPSYCH, and particularly in the stratum with highest numbers (>=6). Stratified analyses are shown below. Interestingly there is a high interaction estimate in the right direction in the group pf children with >= 6 hospitalizations in line with that seen in COPSACsevere. This is in line with a strong main effect for *FUT2* (OR = 1.66 (95%CI = 0.99 – 2.38), P = 0.06) in iPSYCH in this stratum (Supplementary Table 2).

The table below summarises the *FUT2-ABO* interactions stratified by the number of hospitalizations in iPSYCH and in COPSACsevere for comparison. The functional *FUT2* variant is used for the analysis. This has now been added as a Supplementary Table 10).

Disease stratum	COPSACsevere			iPSYCH		
	Cases	OR [95% CI]	P	Cases	OR [95% CI]	P
**1**	-	-	-	1236	0.96 [0.85 ; 1.08]	0.48
**2**	276	1.06 [0.83 ; 1.36]	0.62	267	1.05 [0.82 ; 1.35]	0.70
**3**	240	1.24 [0.95 ; 1.62]	0.11	83	0.93 [0.60 ; 1.45]	0.76

**4-5**	293	1.26 [0.99 ; 1.62]	0.06	45	0.95 [0.52 ; 1.73]	0.87
**≥ 6**	279	1.51 [1.21 ; 1.88]	2.4e-04	31	1.56 [0.71 ; 3.47]	0.28

Importantly, there was also significant interaction in the COPSAC birth cohorts where the main effect of *FUT2* was also high. It seems that the interaction estimate, and thereby the power to detect the interaction, increases along the main effect. Our interpretation of this is that a more specific phenotype increases main gene effects and interaction effects due to a more homogenous case population and thereby more homogenous biological signal (avoiding "dilution") - and this increase in effect size is particularly important for interactions where the sample size requirement for statistical power is increased, and a low interaction effect size would require a very large sample size for sufficient statistical power to demonstrate interaction.

7. R218 " Current asthma" ; how defined?

R41. After the diagnosis was given, following the algorithm described above (R34), regular (yearly) trials of cessation of controller medication were performed. Current asthma by age 6 was defined as asthma still requiring controller medication (due to relapse after cessation) during the preceding year.

8. R239 upper respiratory symptoms: which symptoms

R42. The children scored cold symptom severity based on a 4-point scoring system (0=no symptoms, 1= "Mild stuffy or runny nose but does not affect daily activity", 2="Moderate stuffy or runny nose and reduced activity but does not affect sleep", 4="Cannot breathe through the nose and not able to sleep well because of symptoms".

It was shown in a previous study from Rhinogen (Kloepfer, K.M. *et al. J Allergy Clin Immunol* **133**, 1301-7, 1307 e1-3 (2014).) that presence of S Pneumoniae was related to cold symptom burden, and we therefore hypothesized that this would be more pronounced in children with *FUT2/ABO* risk genotypes. As hypothesized, *S. pneumoniae* was only associated with cold symptom burden in children with risk genotype, and furthermore the *FUT2-ABO* risk score was

associated with increased symptom burden only during episodes with detection of *S. pneumoniae* and not during episodes without detection of *S. pneumoniae.* (Fig 5)

9. Discussions, when discussing previous evidence from other studies on the ABO locus (line 289 – 295), please add sample size and phenotype definition of these study to the discussion.

R43. As per the reviewer's suggestions the sample size and phenotype definitions have been updated in the revised manuscript.

10. Table 3. Please specify for which phenotype the interactions are shown

R44. Table 3A is based on the $COPSAC_{severe}$ cohort, and is therefore hospitalizations due to asthma exacerbations during the first 6 years of life.

Table 3B reports the interaction in the COPSAC birth cohorts where the phenotype is defined as any asthma diagnosis during the first 6 years of life as described above (**R34**).

11. Figure 6, two different Y axis: Rate of infections and Symptoms burden are shown, please explain the differences

R45. Thanks to the reviewer for the comment. These differences are based on the different study designs in the two studies.

In the COPSAC birth cohorts, children are invited to the clinic when they have acute symptoms, and the microbial trigger at this time point is assessed, including s pneumoniae. The outcome based on the COPSAC data is therefore the number of acute reparatory illnesses with a given bacterial or viral trigger, and the statistical model used is a quasi poisson regression.

This is different from the COAST cohort, where children are seen at scheduled visits where the presence of s pneumoniae as well as concurrent symptoms are registered as a continuous outcome. This requires another statistical model testing whether symptom burden in relation to *S. pneumoniae* presence is affected by *FUT2×ABO* genotype. Thus, the statistical models used to

estimate the effect of the *FUT2-ABO* on s pneumoniae related symptoms are different which results in different y-axis when illustrating the results.

References:

Moffatt MF, Gut IG, Demenais F, Strachan DP, Bouzigon E, Heath S, et al. A large-scale, consortium-based genomewide association study of asthma. N Engl J Med. 2010; 363(13):1211–21.

Demenais F, Margaritte-Jeannin P, Barnes KC, Cookson WOC, Altmüller J, Ang W, et al. Multiancestry association study identifies new asthma risk loci that colocalize with immune-cell enhancer marks. Nat Genet. 2018; 50(1):42.

Thomsen SF, Duffy DL, Kyvik KO, Backer V. Genetic influence on the age at onset of asthma: a twin study. J Allergy Clin Immunol. 2010;126(3): 626–30.

Pividori, Milton, Nathan Schoettler, Dan L. Nicolae, Carole Ober, and Hae Kyung Im. Shared and Distinct Genetic Risk Factors for Childhood-Onset and Adult-Onset Asthma: Genome-Wide and Transcriptome-Wide Studies. The Lancet Respiratory Medicine 2019; 7 (6): 509–22.

Mahajan, Anubha, Daniel Taliun, Matthias Thurner, Neil R. Robertson, Jason M. Torres, N. William Rayner, Anthony J. Payne, et al. Fine-Mapping Type 2 Diabetes Loci to Single-Variant Resolution Using High-Density Imputation and Islet-Specific Epigenome Maps. Nat Genet 2018; 50 (11): 1505–13.

Reviewer #1 (Remarks to the Author):

My main concerns are largely addressed by the additional analyses provided. Here are a few more points to address in this round of revision.

1- In Table 1 the effect allele frequency is based on which cohort? Similar to the effect size and P-value, can you add a column for the effect allele frequency in each discovery cohort?

2- Table 1 describe the results of the COAST replication cohort these results are not presented in the main text where the COPSAC and UK biobank replication results are described.

3- The numbers in table 1 and the numbers in the main text for FUT2 effect in the replication cohort are different possibly due to rounding please check?

4- At the discovery stage the COPSACsevere and Inter99 cohorts that are independently genotyped are combined to perform the association study. Can the authors provide a PCA of this combined case-control cohort to show that after QC there is no batch effect/stratification? Similarly, COPSAC2000 and the COPSAC2010 were combined for replication analysis. Can the authors provide a PCA of the combined COPSAC cohorts to alleviate concerns regarding batch effect?

5- Please add the case-only interaction analysis and permutation analysis done to provide additional support for the significance of interaction to supplementary data and modify the manuscript's main text and method accordingly.

6- In table 2, where the authors refer to SNPs as functional and top, please also provide rsIDs to avoid confusion.

7- As authors acknowledge, the top association signal in FUT2 locus can point to causal variants in other nearby genes or a causal mechanism different from the one suggested by the authors, via interaction with ABO. The lack of experimental validation should be added to the limitations part of the paper. Additionally, please modify the discussion to mention that despite the potential clinical relevance of the findings their clinical application requires independent replication in future studies as well as functional validation.

8- Given that this is an exome-wide study and does not cover the whole genome many tag or causal variants can be missed in this study. Moreover, the identified variants may tag non-coding variants that could not be identified using fine mapping as they are missed in this study. These points should be added to the limitations section.

9- In general, the authors need to review their manuscript carefully to ensure that numbers and names are presented consistently throughout the manuscript's main text, methods, figures/tables, and supplements.

Reviewer #3 (Remarks to the Author):

This is an intersting paper that thoughtfully addresses the genetic contribution to the phenotype of severe asthma exacerbations in children.

In addition to known loci the authors report a significant association to the FUT2 locus on chromosome 19. They show palusible interactions between FUT2 and the ABO blood group, and an additional association to the presence of S. pneumoniae during exacerbations.

The paper has previously been meticulously reviewed, and the authors have been equally meticulous and careful in their responses.

My only minor observation is that the major chromosome 17 locus contains ORMDL3 as well as GASDMB, and that both genes seem under control of a single promotor. ORMDL3 has effects on sphingolipid metabolism that may interact with the actions of FUT2, so it might be wise en passant to mention it.

Otherwise, this is an excellent paper from an excellent collaboration.

Response to reviewer comments:

Reviewer #1

R1. In Table 1 the effect allele frequency is based on which cohort? Similar to the effect size and P-value, can you add a column for the effect allele frequency in each discovery cohort?

REPLY: The reported allele frequencies for the discovery meta analysis were the weighted average of iPSYCH and $\text{COPSAC}_{\text{severe}}$ participating studies. As per the reviewers request, we now instead show the study-specific effect allele frequencies (EAF) in table 1.

R2. Table 1 describe the results of the COAST replication cohort these results are not presented in the main text where the COPSAC and UK biobank replication results are described.

REPLY: We thank the reviewer for noticing this. This result has now been added to the revised manuscript: "*We found evidence of association between the rs281379 SNP and childhood asthma at the age of 6 years in the COAST cohort (OR = 1.79 (95% CI = 1.00-3.30), P = 0.05) with the combined meta-analysis (discovery and replication stages) remaining genome-wide significant (OR = 1.20 (95% CI = 1-13-1.27), $P_{combined} = 9.2 \times 10^{-10}$)*".

R3. The numbers in table 1 and the numbers in the main text for FUT2 effect in the replication cohort are different possibly due to rounding please check?

REPLY: Thanks to the reviewer for pointing this out. It has now been corrected.

R4. At the discovery stage the COPSACsevere and Inter99 cohorts that are independently genotyped are combined to perform the association study. Can the authors provide a PCA of this combined case-control cohort to show that after QC there is no batch effect/stratification? Similarly, COPSAC2000 and the COPSAC2010 were combined for replication analysis. Can the authors provide a PCA of the combined COPSAC cohorts to alleviate concerns regarding batch effect?

REPLY:
A PCA plot (PC 1 vs PC2) for the $\text{COPSAC}_{\text{severe}}$ and Inter99 cohorts is shown below (Figure 1). As seen in the plot, there is no general shift indicating batch effects or stratification although there is a smaller number of individuals positioned in the

periphery with low values for PC1, all belonging to the Inter99 cohort (N=104 individuals with PC1 value < 0.01). This is probably due to strict ethnicity filtering of children in the COPSACsevere cohort, which was done before exome chip genotyping and was stricter than the filtering used in the QC for this study. However, as we included the first 10 principal components as covariates in the logistic regression model this effect is accounted for in the reported results (Table 1, Supplementary Table 2 and Supplementary Table 15).

Figure 1. PC1 vs. PC2 for the COPSACsevere and Inter99 cohorts

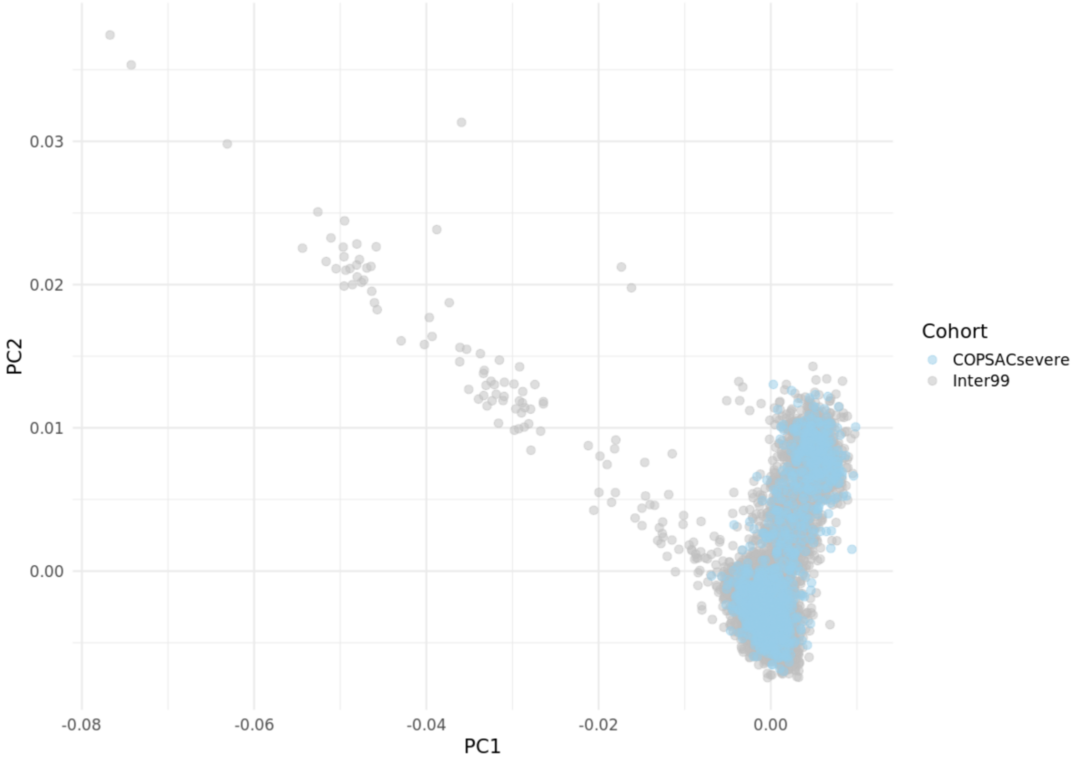

To further test that these outliers did not inflate our results, we have rerun analyses after removal of the 104 outliers (individuals with PC1 value < 0.01). As shown in the table below (Table 1), this resulted in largely unchanged results.
Importantly, this was also true for the FUT2/MAMSTR locus (rs281379), which was actually slightly more significant after removal of outliers. Also of importance, the *FUT2-ABO* interaction result was completely unchanged after outlier removal (OR=1.29 [1.12;1.48], P=3.22e-04).

These analyses confirm that batch effects/stratification did not bias our findings.

Table 1. Discovery results for COPSACsevere and the combined discovery cohorts after removal of 104 outliers from the PCA analysis. A) Before removal of outliers and B) after removal of outliers.

A)

Locus	$COPSAC_{severe}$		Combined discovery	
	OR [95% CI]	P-value	OR [95% CI]	P-value
rs7219923	2.12 (1.93-2.33)	8.6×10^{-54}	1.65 (1.56-1.75)	1.61×10^{-68}
rs6967330	1.50 (1.34-1.69)	3.9×10^{-12}	1.41 (1.32-1.51)	2.1×10^{-23}
rs1071630	1.36 (1.23-1.49)	6.9×10^{-10}	1.25 (1.18-1.32)	8.0×10^{-14}
rs340933	1.51 (1.32-1.74)	5.8×10^{-9}	1.37 (1.26-1.49)	1.6×10^{-13}
rs1342326	1.46 (1.30-1.63)	5.1×10^{-11}	1.31 (1.22-1.40)	1.7×10^{-13}
rs10189629	1.47 (1.25-1.73)	2.1×10^{-6}	1.40 (1.27-1.54)	7.7×10^{-12}
rs1043828	1.26 (1.15-1.38)	8.1×10^{-7}	1.20 (1.14-1.27)	1.0×10^{-10}
rs20541	1.34 (1.20-1.49)	1.2×10^{-7}	1.21 (1.13-1.29)	1.0×10^{-8}
rs281379	1.18 (1.08-1.30)	2.6×10^{-4}	1.18 (1.11-1.25)	2.6×10^{-9}

B)

Locus	$COPSAC_{severe}$		Combined discovery	
	OR [95% CI]	P-value	OR [95% CI]	P-value
rs7219923	2.07 [1.88 ; 2.28]	9.87e-49	1.63 [1.54 ; 1.72]	5.84e-65
rs6967330	1.50 [1.34 ; 1.69]	5.34e-12	1.40 [1.31 ; 1.51]	1.12e-22
rs1071630	1.39 [1.24 ; 1.55]	3.65e-09	1.24 [1.17 ; 1.32]	8.93e-13
rs340933	1.48 [1.29 ; 1.71]	7.18e-08	1.36 [1.25 ; 1.48]	5.13e-13
rs1342326	1.44 [1.28 ; 1.61]	1.22e-09	1.31 [1.21 ; 1.40]	1.71e-13
rs10189629	1.45 [1.23 ; 1.71]	8.08e-06	1.39 [1.26 ; 1.53]	3.67e-11
rs1043828	1.26 [1.15 ; 1.39]	1.41e-06	1.20 [1.14 ; 1.27]	3.43e-10
rs20541	1.34 [1.20 ; 1.49]	1.68e-07	1.21 [1.13 ; 1.29]	1.36e-08
rs281379	1.23 [1.12 ; 1.35]	1.28e-05	1.20 [1.13 ; 1.26]	1.37e-10

A PCA plot for the COPSAC cohorts showed no differences between the cohorts (Figure 2).

Figure 2. PC1 vs. PC2 for the COPSAC birth cohorts (2000 and 2010)

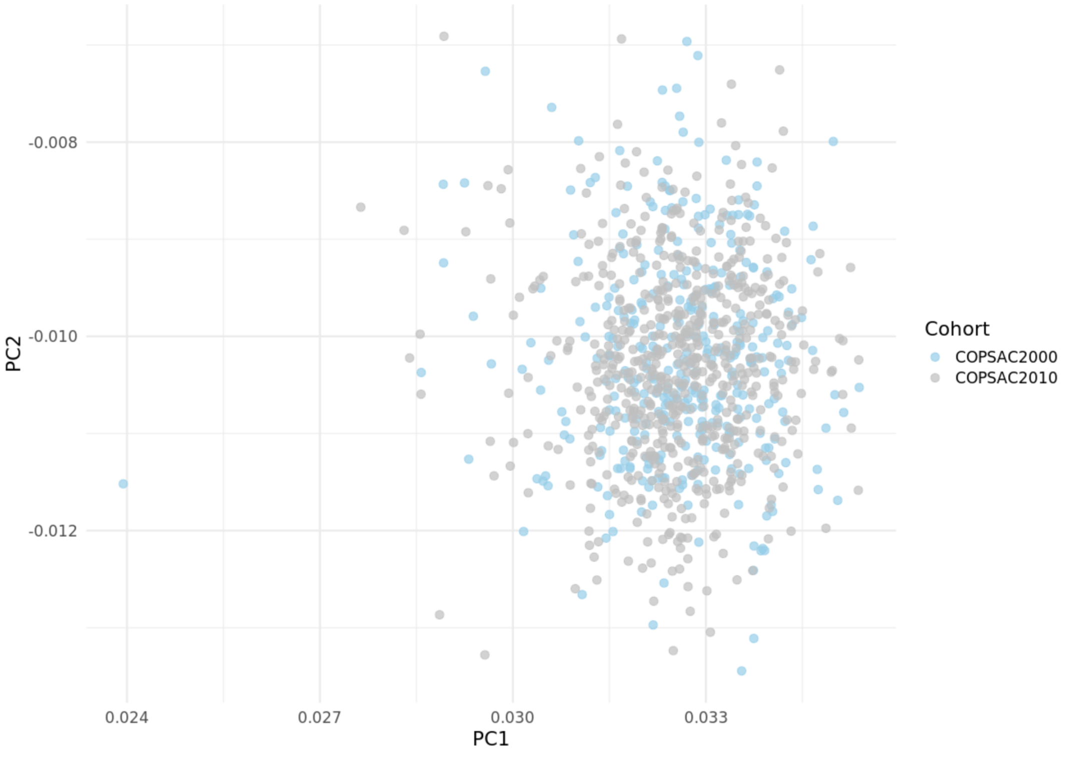

R5. Please add the case-only interaction analysis and permutation analysis done to provide additional support for the significance of interaction to supplementary data and modify the manuscript's main text and method accordingly.

REPLY: We thank the reviewer for this suggestion. The case-only analysis table has now been added to the supplementary materials (Supplementary Table 11). Furthermore, it is also described in the main text:
*"Case-only analysis also showed the same severity-based dependency, as we only observed a significant correlation between the FUT2 and ABO SNPs using the most severe cases (Correlation = 0.17 (95% CI = 0.07 ; 0.27), P = 9.2 × 10^{-4}) (**Supplementary table 11**).... To investigate the likelihood of observing the same severity-driven interaction signal for the COPSAC$_{severe}$ cohort, we performed 10.000 phenotype permutations, and observed 1 interaction which showed an interaction*

effect with similar statistical significance in both the overall analysis and most severe strata".
The permutation analysis is also described under the methods section in the revised manuscript.

R6. In table 2, where the authors refer to SNPs as functional and top, please also provide rsIDs to avoid confusion.

REPLY: This has now been added.

R7. As authors acknowledge, the top association signal in FUT2 locus can point to causal variants in other nearby genes or a causal mechanism different from the one suggested by the authors, via interaction with ABO. The lack of experimental validation should be added to the limitations part of the paper. Additionally, please modify the discussion to mention that despite the potential clinical relevance of the findings their clinical application requires independent replication in future studies as well as functional validation.

REPLY: As suggested, this limitation has now been added:
"However, any clinical application would require several additional steps, including replication in different populations, establishing causality behind our findings, and randomized trials showing improved clinical benefit according to genotype''.
and
"It is a limitation of the study that we did not provide experimental data to support the causal mechanisms underlying the observed gene-gene interaction."

R8. Given that this is an exome-wide study and does not cover the whole genome many tag or causal variants can be missed in this study. Moreover, the identified variants may tag non-coding variants that could not be identified using fine mapping as they are missed in this study. These points should be added to the limitations section.

REPLY: Through the added genotyping, the genome-wide coverage has now been markedly increased, including a large number of non-exome SNPs, but we agree that the study still has limited genomic resolution. This limitation has now been added in the revised manuscript:
"*Another limitation is the reduced genomic resolution with an inherent risk of missing important susceptibility variants, including the true causal variants at the genome-wide significant loci.*"

R9. In general, the authors need to review their manuscript carefully to ensure that numbers and names are presented consistently throughout the manuscript's main text, methods, figures/tables, and supplements.

This is an interesting paper that thoughtfully addresses the genetic contribution to the phenotype of severe asthma exacerbations in children.

REPLY: We will make sure that the numbers and names are presented consistently throughout the manuscript text, figures/tables, and supplements, and we thank the reviewer for the appreciation of our study.

Reviewer #3

R.10 My only minor observation is that the major chromosome 17 locus contains ORMDL3 as well as GASDMB, and that both genes seem under control of a single promotor. ORMDL3 has effects on sphingolipid metabolism that may interact with the actions of FUT2, so it might be wise en passant to mention it.

REPLY: We thank the reviewer for drawing our attention to this. We are aware that the 17q locus might involve both GSDMB and ORMDL3, which affects sphingolipid metabolism, and see that FUT2 has been mapped as part of the Glyco sphingolipid superpathway through the protein-protein interactions data (https://pubmed.ncbi.nlm.nih.gov/25853426/) in immune mediated diseases. However, since the 17q locus is already addressed extensively in previous studies, and we do not study interaction between the FUT2 locus and the 17q locus in the current paper, we decided not to mention this in the discussion.
